# Li, Na, K, Mg, Zn, Al, and Ca Anode Interface Chemistries Developed by Solid‐State Electrolytes

**DOI:** 10.1002/advs.202304235

**Published:** 2023-09-24

**Authors:** Sambhaji S. Shinde, Nayantara K. Wagh, Sung‐Hae Kim, Jung‐Ho Lee

**Affiliations:** ^1^ Department of Materials Science and Chemical Engineering Hanyang University Ansan Gyeonggi‐do 15588 Republic of Korea; ^2^ FLEXOLYTE Inc. Ansan 15588 Republic of Korea

**Keywords:** future perspectives, insights for nucleation deposits, interface issues, ion‐transport mechanisms, Li, Na, K, Mg, Zn, Al, and Ca anode interface chemistry, plate/strip for reversible anodes, state‐of‐the‐art SEs, thermodynamics and chemical kinetics

## Abstract

Solid‐state batteries (SSBs) have received significant attention due to their high energy density, reversible cycle life, and safe operations relative to commercial Li‐ion batteries using flammable liquid electrolytes. This review presents the fundamentals, structures, thermodynamics, chemistries, and electrochemical kinetics of desirable solid electrolyte interphase (SEI) required to meet the practical requirements of reversible anodes. Theoretical and experimental insights for metal nucleation, deposition, and stripping for the reversible cycling of metal anodes are provided. Ion transport mechanisms and state‐of‐the‐art solid‐state electrolytes (SEs) are discussed for realizing high‐performance cells. The interface challenges and strategies are also concerned with the integration of SEs, anodes, and cathodes for large‐scale SSBs in terms of physical/chemical contacts, space‐charge layer, interdiffusion, lattice‐mismatch, dendritic growth, chemical reactivity of SEI, current collectors, and thermal instability. The recent innovations for anode interface chemistries developed by SEs are highlighted with monovalent (lithium (Li^+^), sodium (Na^+^), potassium (K^+^)) and multivalent (magnesium (Mg^2+^), zinc (Zn^2+^), aluminum (Al^3+^), calcium (Ca^2+^)) cation carriers (i.e., lithium‐metal, lithium‐sulfur, sodium‐metal, potassium‐ion, magnesium‐ion, zinc‐metal, aluminum‐ion, and calcium‐ion batteries) compared to those of liquid counterparts.

## Graphical abstract

Comprehensive analysis of the recent innovations in anode interface chemistries developed by solid‐state and liquid electrolytes is highlighted with monovalent (Li^+^, Na^+^, K^+^) and multivalent (Mg^2+^, Zn^2+^, Al^3+^, Ca^2+^) cation carriers (i.e., lithium‐metal, lithium‐sulfur, sodium‐metal, potassium‐ion, magnesium‐ion, zinc‐metal, aluminum‐ion, and calcium‐ion batteries). The interface challenges and strategies for integrating solid‐state electrolytes, anodes, and cathodes for large‐scale SSBs are discussed.

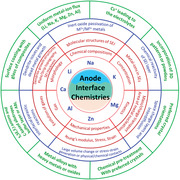

## 1 Introduction

Recent battery systems, including lithium‐ion, lead‐acid, nickel‐metal hydride, and flow batteries, have been utilized for electric vehicles, portable electronics, and grid‐scale stationary storage; however, they do not satisfy projected energy, lifetimes, cost, and safety demands.^[^ ^1^, ^2^ ^]^ Batteries with high energy density, long operational life, and safety standards with reasonable prices are critical requirements of the industry.^[^ ^3^, ^4^ ^]^ Advanced Li‐ion batteries (LIBs) suffer from their energy limits and are challenged by energy‐storage market demands of ≥500 Wh kg^−1^ at the cell level.^[^ ^5^ ^]^ However, serious safety issues, limited energies, and electrochemical/thermal instabilities due to flammable organic liquid electrolytes are the main problems of existing LIBs. Solid‐state batteries (SSBs) comprising solid‐state electrolytes (SEs) have received remarkable scientific and industrial attention for electrical energy storage with promising new battery chemistries. SSBs yield inspiring benefits such as 1) improving safety anxieties by the removal of flammable organic liquids, 2) inhibiting short‐circuit failures by delaying metal dendrites, 3) bending, punching, or piercing deprived of risky annoying safety hazards, 4) larger electrochemical windows (EWs) utilizing high voltage cathode materials.^[^ ^6^ ^]^ SSBs consist of bulk‐ and thin‐film types. Bulk‐type SSBs exhibit the compressing active materials in pellets and stacks or preparing slurries with tape casting owing to their high‐energy, low cost, and safe, which illustrates suitability for future energy‐storage systems. Thin‐film SSBs exhibit thin layered active materials loading and applicability for micro‐batteries.^[^ ^7^ ^]^ Researchers anticipated SSBs would reveal multiple advantages, including high reversibility and acceptable cycle life with broad temperature operations. Also, SEs should possess the major standard parameters of M^+^‐ion conductance (>10^−4^ S cm^−1^), sufficient mechanical strengths (shear modulus G_separator/electrolyte_ > G_anode_), and slightest defects to prevent metal‐dendrites infiltration, lower activation energies for metal M^+^‐diffusion (lower the surface diffusion barriers of M^+^‐ions with high surface energies), and scalable fabrication processes with abundant resources.^[^ ^8^, ^9^ ^]^

SEs research initiates with high modulus solid‐ion conductors that can project direct pathways for practical high‐energy batteries with metal anodes.^[^ ^1^ ^]^ Recently SEs focused on several developments involving high‐performance materials, enlarged safety issues, and different applications. SEs comprise organic (polymers) and inorganic (oxides, sulfides, hydrides) solids. Numerous SEs with different chemistries display superb ion conductivity at room‐temperature (≈1 mS cm^−1^) relative to those of flammable liquid electrolytes. Sulfide‐based inorganic SEs display higher ion‐conductivities than those of all other SEs (oxides, polymers, or composites) owing to their body‐centered‐cubic (*bcc*)‐like anions structures, which is favorable for Li‐ion or other metal‐ions (such as Na, K, Zn, Mg, Al, Ca) diffusion.^[^ ^6^ ^]^ Precisely, the inorganic SEs, Li_10_GeP_2_S_12_ (12 mS cm^−1^), Li_7_P_3_S_11_ (17 mS cm^−1^), Li_9.54_Si_1.74_P_1.44_S_11.7_Cl_0.3_ (25 mS cm^−1^), and Li_2_S‐P_2_S_5_ (17 mS cm^−1^) as intrinsic single‐ion conductors manifest high charge‐transfer capability comparable to liquids, illustrating great promise for future high‐energy batteries.^[^ ^10^, ^11^, ^12^ ^]^ However, the solid‐solid interfaces are the key scientific concern that limits the practical applications of SSBs. For instance, Li‐ions diffuse across anode/SEs and SEs/cathode interfaces for transport. Simultaneously, electrons leave one side of battery cells upon discharge through current collectors (CCs)/anode interfaces and arrive on the opposite side via cathode/CC interfaces and vice‐versa upon charge. The oxidation/reduction reactions for electrode/SEs interfaces are the primary source for interfacial reactions in SSBs. A comprehensive understanding of this interface kinetics is critically necessary for stable operations for SSBs.

Using metal anodes (Li, Na, K, Mg, Zn, Al, Ca) has been investigated to find a promising approach for high‐energy storage technologies. In principle, replacing graphite (372 mAh g^−1^) anode with Li (3860 mAh g^−1^) or other metals makes it possible to reach an energy density of ≥500 Wh kg^−1^ with suitable battery chemistries.^[^ ^5^, ^9^ ^]^ Unlike graphite, however, metal anodes are highly reactive, have poor mechanical yields, are inclined to large volume deviations upon charge‐discharge processes, and have numerous metal‐ions concentrations for electrochemical reactions. The solid‐electrolyte interphase (SEI) plays a significant role in determining metal anodes' reversible cycle life.^[^ ^13^ ^]^ SEI allows the metal‐ions diffusion to preserve reaction kinetics and electronic insulation among the electrodes and SEs (i.e., limits parasitic reactions), promoting uniform metal electrodepositions by controlling solid ion flux. Ideal properties required for the SEI layer are superb cations conductivity with larger electronic resistances, uniform thickness (10–20 nm), larger mechanical strengths that facilitates the tolerance for volumetric changes upon charging, insolvability in the electrolytes, and stable operating capabilities for high voltages and wide temperatures. The robust SEI is essential for long‐life battery operations for metal or ion‐insertion anodes. SEI growth (homogenous or heterogenous, rapid or gradual) features are the key indicator predicting dendritic growth or anode/SEs interface kinetics.^[^ ^14^, ^15^, ^16^ ^]^ Considering the different improvements of SSBs for Li, Na, K, Mg, Zn, Al, and Ca relative to those of liquid counterparts, it is essential to critically evaluate the present status and prospects for SEs and SEs/anode interfaces with distinctive battery chemistries.

This review begins with the thermodynamics, chemistries, and electrochemical kinetics for desirable SEI formation required for practical reversible metal anodes. The experimental and theoretical insights have been summarized for the nucleation of metals, electrodepositions, and stripping for reversible metal anodes. We discuss ion‐transport mechanisms, state‐of‐the‐art of SEs, and interface challenges for realizing high‐performance cells. Then we focus on recent innovations for various anode interface chemistries enabled by SEs, including monovalent (Li^+^, Na^+^, K^+^) and multivalent (Mg^2+^, Zn^2+^, Al^3+^, Ca^2+^) cation carriers (for example, lithium‐metal, lithium‐sulfur, sodium‐metal, potassium‐ion, magnesium‐ion, zinc‐metal, aluminum‐ion, and calcium‐ion systems). Finally, the challenges and future perspectives for SEs, reversible anodes, and electrode/SEs interface chemistries and technologies are summarized.

## 2 Fundamentals of Thermodynamics and Electrochemical Kinetics for Desirable SEI

**Figure 1:**
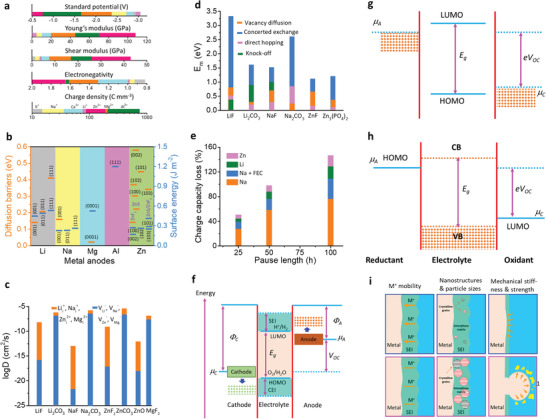
a) Intrinsic physicochemical properties of different metals.^[^ ^18^ ^]^ b) The diffusion barriers and surface energies of various metals (Li, Na, Mg, Al, Zn, ZnS, ZnF_2_) for different crystal reflections.^[^ ^9^, ^19^ ^]^ c) Self‐diffusion coefficients for Li‐, Na‐, Zn‐, and Mg‐SEI. Li_i_^+^, Na_i_^+^, Zn_i_^2+^, or Mg_i_^2+^ defines the excess interstitial Li‐, Na‐, Zn‐, and Mg‐ions. V_Li_^−^, V_Na_^−^, V_Zn_^−^, and V_Mg_^−^ denote the negatively charged Li, Na, Zn, and Mg vacancies.^[^ ^20^, ^21^, ^22^, ^23^, ^24^ ^]^ d) Migration energy barriers for various hetero‐diffusion kinetics. e) Charge capacity loss of Li, Na, and Zn metals inclined by SEI dissolution.^[^ ^25^, ^26^ ^]^ f) Schematics energy states for electrolytes and electrodes that facilitate the SEI and CEI formation. μ_C_ and µA refer to the energy levels of cathode and anode electrodes. LUMO and HOMO define the electrolytes' lowest unoccupied molecular orbital and highest occupied molecular orbital. CB and VB imply the conduction and valence bands, respectively. VOC represents the open‐circuit voltage of the cells. Reproduced with permission.^[^ ^36^ ^]^ Copyright 2009, American Chemical Society. g) Electrochemical potential window for liquid electrolytes. h) Electrochemical potential window for solid electrolytes. Reproduced with permission.^[^ ^4^ ^]^ Copyright 2013, American Chemical Society. i) Schematics represent the SEI contributions to the flat and dense metal electrodeposition (positively in the top blue boxes and negatively in the bottom purple boxes).^[^ ^49^ ^]^

The rational design of next‐generation batteries with various single/multivalent metals (Na, Li, K, Mg, Al, Zn, and Ca) is of significant attention. Since 1970,^[^ ^17^ ^]^ the SEI formation at the anode surface has been regarded as one of the key parameters for liquid/solid batteries. Several strategies have been demonstrated for the development of SEI of different metals. The physicochemical properties of Na and K are comparable with Li; however, they possess more electronegativity resulting in different modulus and solubility of SEI formulations (**Figure** **1**a).^[^ ^18^ ^]^ Given Mohs hardness and bulk/shear modulus, Na and K are softer than Li metal, indicating the suppression of dendrites is considerable. In contrast, the SEI formation for multivalent metals has yet to be explored. The high modulus of Al and Zn implies severe dendrite growth. Besides, a larger charge density of multivalent ions validates the poor mobility of ions in the SEI that limits the robust construction of SEI. Theoretical calculations clarify the lowest diffusion barriers for Mg compared to those of Li, Na, Zn, and Al (Figure 1b), demonstrating smooth structure formation. Hexagonal closest packed (HCP) structures of Mg and ZnS/ZnF_2_‐Zn(101) favor high‐coordination configurations than those of Li and Na (face‐centered/body‐centered cubic; FCC/BCC) metals.^[^ ^9^, ^19^ ^]^

Theoretical calculations also show that LiF, NaF, and ZnF have favorable ionic transport by vacancy defects (Figure 1c). Li‐ion has a faster transport behavior by LiF due to high diffusion coefficients compared to Na‐ion by NaF and Zn‐ion by ZnF.^[^ ^20^, ^21^, ^22^, ^23^, ^24^ ^]^ Both Li^+^ and Na^+^ can be migrated by Na‐SEI (NaF and Na_2_CO_3_) and Li‐SEI (LiF and Li_2_CO_3_) via direct hopping or knock‐off or vacancy mechanisms (Figure 1d).^[^ ^20^ ^]^ The low polarity of Na^+^ provides the most stable Na‐SEI compared to those of Li‐SEI, implying poorer stripping/plating efficiency for Na compared to Li and larger capacity loss, especially in carbonate electrolytes (Figure 1e).^[^ ^25^ ^]^ The inorganic NaF and Na_2_O strongly contribute to the high Coulombic efficiency (CE) of 99.9% for Na plating/stripping in the NaPF_6_ in diglyme electrolytes.^[^ ^26^ ^]^ The construction of stable Li‐SEI over the Na‐metal (heterostructural design) has been clarified as the alternative approach to shield the Na‐metal‐anode. Fluoroethylene or vinylene carbonate (FEC/VC), and di‐oxolane (DOL) for inorganic/organic SEI show similar chemical properties for both Na and Li, while NaNO_3_ has adverse effects for Na than ideal LiNO_3_ for Li because of the severe decomposition of electrolytes. In Li/Na hybrids, the more negative redox potential of Li/Li^+^ offers the shielding effect for Na^+^ that controls the dynamic interphase for dense electrodeposition.^[^ ^27^, ^28^ ^]^ The softest metal among the anodes is K which has similar properties to Na and Li; however, the high reactivity of K with solvents forms unstable SEI even for a small current density (0.2 mA cm^−2^). Highly concentrated electrolytes can stabilize SEI for K effectively.^[^ ^29^ ^]^

The high polarization occurs due to low ionic radius and large charge number. Ca and Mg sustain reversible plating/stripping with greatly destructive and lower oxidative stable electrolytes. In contrast, carbonate electrolytes tend to have poor conductivity in SEI with the degradation of plate/strip processes. Al undergoes reversible plate/strip with highly corrosive ionic liquids (AlCl_3_ + 1‐ethyl‐3‐methylimidazolium chloride). Artificial SEI enables the operations of Ca, Mg, and Al under non‐aqueous/aqueous electrolytes, which vindicates the impact of SEI on multivalent metals.^[^ ^30^, ^31^, ^32^ ^]^ Zn compatibility with water enables intrinsic safety, whereas dendrite formation and volume change limit the commercial application for larger Zn utilizations. Zn metal typically practices Zn_5_(CO_3_)_2_(OH)_6_ layer at ambient conditions that easily oxidized in mild acids and conveyed to movable interphase of Zn_4_SO_4_(OH)_6_.xH_2_O, which results in low CEs and severe dendritic‐growth.^[^ ^33^ ^]^ For alkaline media, the creation of soluble ZnO_2_^2−^ and irreversible ZnO on the anode suffers structural distortions. Cation‐selective membranes, polymer‐based additives, epitaxial electrodeposition with lower lattice mismatch, and artificial ZnS/ZnF_2_‐based SEI have been applied to protect the Zn anode.^[^ ^9^, ^34^, ^35^ ^]^

Figure 1f–h demonstrates the energy states of electrodes and the chemical stability of electrolytes. Apart from high ion conductivity or transfer number and compatible mechanical properties, the lowest unoccupied molecular orbital (LUMO) or conduction band (CB) should be higher than the chemical potential (*μ_A_*, or Fermi energy) of the anode and the highest occupied molecular orbital (HOMO) or valence band (VB) should be lower than the *μ_C_* of the cathode which are the most notable standards for designing liquid or solid electrolytes (Figure 1f–h).^[^ ^36^, ^37^, ^38^, ^39^ ^]^ The energy gap (*E_g_*) between LUMO (or CB) and HOMO (or VB) of electrolytes determines the thermodynamically stable electrochemical potential window and driving force to practice the SEI layer for solid or liquid electrolytes with high‐voltage cathodes and metal anodes, which is significant for high‐energy secondary batteries. Further, the difference in chemical potential of the cathode (*μ_C_*) and anode (*μ_A_*) corresponds to the open‐circuit potential of the cells (Equation 1).^[^ ^39^ ^]^

(1) $$eV_{\mathrm{OC}}=\mu_{A}-\mu_{C}\leq E_{g}$$

where *e* is the electron charge magnitude. If the Fermi energy of the anode is higher than the LUMO of electrolytes, then electrolyte reduction ensues at the anode interface to form an SEI. On the other hand, if the Fermi energy of the cathode is lower than the HOMO of electrolytes, electrolyte oxidation follows for the cathode interface to form a cathode‐electrolyte interphase (CEI). The electrochemical window is projected by the onset points of current–voltage curves (i.e., oxidation voltage of electrolyte) using linear sweep voltammetry.

Notably, the SEI allows the metal‐ions (Li, Na, Mg, Zn, K, Ca, Al) diffusion through a layer under a uniform electric field with reducing concentration polarization and the overpotential, which prevents the aggregation of electrochemically active species with maintaining uniform chemical compositions of electrodes. Besides, thickening the SEI layer likely increases the internal resistance of the cell by consuming metal ions from cathodes, diminishing the capacity and power. Under the carbonate‐based electrolytes, the SEI layer forms at ≈1 V versus Li/Li^+^ or Na/Na^+^ for the metals, oxides, or carbons.^[^ ^40^ ^]^ Anode materials with K‐storage voltages are ≈0–1 V*_K_*. For example, K‐metal (0 V*_K_*), graphite (≈0.1 V*_K_*), alloys (≈0.8 V*_K_*), and red phosphorus (≈0.7 V*_K_*) possess higher Fermi energy than LUMO.^[^ ^41^, ^42^, ^43^ ^]^ Mixed ionic and electronic conducting behavior of SEI/CEI deteriorates the overall cell performances. Inert materials protection for anode/cathode has shown a promising approach to alleviate chemical reactions. Interphase reactions show vital characteristics to reach the full potentials of batteries;, e.g., Li^+^ ions diffuse via grain boundaries and Schottky vacancies of bulk electrolytes, in which electronegativity, contact circumstance, and interphase structures strongly influence the overall energy delivery of cells.^[^ ^14^, ^44^ ^]^

The cation‐anion or cation‐solvent interactions strongly influence the reduction stabilities of solvents/anions as they control the solvation structures and the solvent coordination numbers nearby the cation centers. When cations coordinate with solvents or anions, their LUMO levels decline due to the donation of electron pairs to the cations. Thus, the formation of ion pairs and solvation promotes the decomposition of electrolytes. DFT calculations display the lower LUMO levels of ion‐solvent complexes (i.e., FEC, propylene carbonate (PC), diethyl carbonate (DEC), ethylene carbonate (EC), 1,2‐dimethoxyethane (DME), and 1,3‐dioxolane (DOL)) compared to pure solvents.^[^ ^29^, ^45^, ^46^ ^]^ Lower LUMO level magnitudes of carbonate solvents are in the order of Li^+^ > Na^+^ > K^+^, revealing a linear correlation with binding energy. In contrast, ether solvents demonstrate larger LUMO level changes in Na^+^ > K^+^ > Li^+^.^[^ ^45^ ^]^ SEI formation and reduction stability of electrolytes are influenced by cations with electrolytes, which requires the understanding of the dissimilarities for the SEI layer among Na, Li, K, Zn, Mg, Al, and Ca batteries.

In the perspective of electronic structures for aqueous batteries, water is the oxide having E_g_ of 8.7–8.9 eV. If the LUMO‐HOMO energy gap reflects the EW, water could be solvent for Na, K, or Zn‐ion batteries. The thermodynamic potential of water is 1.23 V, which limits the hydrogen evolution at −4.02 eV and oxygen evolution at −5.25 eV (i.e., 0 V vs SHE at pH 7) corresponding to the energy level of −4.44 eV at absolute scale (electron energy of 0 eV in vacuum). However, there is no correlation between the LUMO–HOMO energy gaps of water and Fermi levels of the electrons for the oxidation and reduction of water in the solutions.^[^ ^47^, ^48^ ^]^ The actual chemical potentials comprise the impact of the surface potentials.

Figure 1i illustrates how SEI properties can contribute the even metal electrodepositions (positively or negatively). The ideal SEI metal deposit over the electrode surface calls for three parameters: 1) high M^+^ mobility is desired, 2) small crystalline particles permit the large uniformity, 3) robust SEI (high elastic modulus and yield strength) overpowers the whisker nucleation by suppressing metal protrusions, whereas the small yield strength permits the whisker growth in the electrolytes. The diffusion through SEI for cation desolvation, exchange current density, and charge transfer influences the exchange current density subject to the rate‐limiting steps. Tuning the solvation energy of cation at the interface via artificial interphases or ionic polymers having functional groups with large affinity to the cations is the suitable approach for minor exchange current density, which enable the in‐plane electrodeposition of metals (Li, Na, Zn).^[^ ^49^, ^50^ ^]^ Insights for ion transports, chemical compositions, nanostructures, and mechanical properties of SEI are fundamental to designing long‐life operative metal electrodes. The intrinsic properties of electrode/electrolytes possess three types of interfaces: robust SEI without chemical side reactions of electrolyte decomposition, SEI with electronic insulation but offers M^+^ transport pathways, and SEI with mixed ionic and electronic conductivity. Constructing the stable artificial passivation layer between the electrodes can balance the interfacial losses with a compatible M^+^‐conducting layer, which can apply to practical applications.^[^ ^51^ ^]^

## 3 Ion Transport Mechanisms for SEs

The ion transport depends on the distribution and concentrations of defects and ion‐binding sites for crystalline solids. Increasing carrier concentration by insertion of aliovalent cations is a practical approach. High‐valence cations generate cation vacancy/anion interstitials, whereas low valence forms the cation interstitials/anion vacancies. The potential difference redistributes the mobile carriers due to lattice distortion at boundaries and interfaces. Ion diffusion kinetics enabled by Schottky and Frenkel defects comprise the vacancy and diffusion mechanisms (i.e., divacancy, interstitial, interstitial–substitution, and collective, Figure 3a).^[^ ^52^, ^53^ ^]^ Special structures offer superion conductivity without many defects, which involves crystal structures with immobile ions, two or more sublattices, and sublattices for mobile species. Basically, three major parameters are required to obtain high‐ion conductance. 1) Large number of equivalent sites for mobile ions to occupy compared to the number of mobile species; 2) lowest migration energy barriers for nearby accessible sites that can follow hopping; and 3) formation of continuous diffusion pathways.^[^ ^54^, ^55^ ^]^ Nernst‐Planck defines the current density (*j*) by relating the flux of charged species for dilute electrolytes to electrical and chemical potential gradients (∇*φ* and ∇*c_i_*, respectively) and mass conversion.^[^ ^56^ ^]^

(2) $$j=-F^{2}\nabla \varphi \sum_{i}\mu_{i}c_{i}-F\sum_{i}D_{i}\nabla c_{i}+F\bar{\mu }\sum_{i}c_{i}$$

where *F* is the Faraday constant, *µ_i_* is the charged species *i* mobility, *c_i_* is the dissociated ion pairs concentration, *D_i_* is the coefficient of diffusion, and *ū* is the convection velocity for the medium through the ion transfer.

For SE, $\bar{u}$ is very small even for potentials higher than the thermal voltage (*k_B_T/e* = *RT/F* where *k_B_* is the Boltzmann's constant, *T* is the temperature, *R* is the gas constant, and *e* is the electronic charge) and the concentration gradient is smaller for reasonable potential. According to these conditions, Equation 2 can be defined to calculate the conductivity of electrolytes.

(3) $$\sigma =-j/\nabla \varphi =F^{2}\sum_{i}\mu_{i}c_{i}$$

where diffusion coefficient and mobility are related by μ*_i_* = *D_i_*/*RT*.

High ion mobility and concentration of mobile ions required for superion conductivity, as per Equation 3, facilitate simultaneous dissociation of ion pairs and minimal resistance for ion motion. A key to enhancing battery operation capability is maximizing the cation transfer number. The diffusion coefficient is defined with a migration‐free energy (Δ*G*_mig_):

(4) $$D_{i}=\gamma a^{2}f_{o}\exp^{\left(-\Delta G_{\mathrm{mig}/\mathrm{RT}}\right)}$$

where *γ* the geometrical effects factor, *a* is hopping distance, and *f_o_* is the ion hopping frequency.^[^ ^57^ ^]^ Based on Equations 3 and 4, electrical conductivity is,

(5) $$\sigma_{i}=F^{2}/\mathrm{RT}\times \left[c_{i}\gamma a^{2}f_{o}\exp^{\left(\Delta S_{\mathrm{mig}/R}\right)}\exp^{\left(-\Delta H_{\mathrm{mig}/\mathrm{RT}}\right)}\right]$$

where Δ*S*_mig_ and Δ*H*_mig_ the entropy and enthalpy of migration, respectively. Further, thermal activation of dissociation of ion pairs relates the *c_i_* with formation enthalpy (ΔH_f_) as,^[^ ^58^ ^]^

(6) $$c_{i}=c_{o}\exp^{\left(-\Delta H_{f/\mathrm{RT}}\right)}$$

where *c_o_* is the initial concentration of ion pairs before thermal activation. Considering the different activation energies as a single barrier (*E_a_*), Equation 5 defines the Arrhenius form as,

(7) $$\sigma =\sigma_{o}/T\times \exp^{\left(-Ea/RT\right)}$$

where *σ_o_* is weak for temperature and *E_a_* = ΔH_f_ + ΔH_mig_ is the total activation energy for the formation and migration steps. Inorganic or crystalline ion conductors obey the Arrhenius Equation 7.

**Figure 2:**
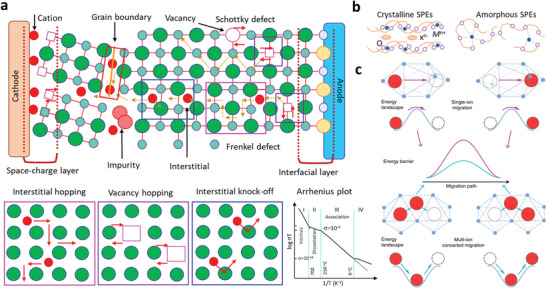
a) Ion‐migration kinetics for SIEs based on the Frenkel and Schottky defects (i.e., vacancy, interstitial and interstitial knock‐off hopping), grain boundaries. Depending on operating conditions, the interface layer formed due to different chemical potentials of electrodes in contact can boost or impede ion‐migration kinetics. b) Ion migration for amorphous and crystalline SPEs. For crystalline, conduction follows through ordered domains generated by polymer segments, whereas for amorphous, migration and hopping of M^+^‐ions are based on the motion of polymer segments. c) Migration pathways for single (upper)/multiple (lower) ions. Reproduced under the terms of a Creative Commons CC BY 4.0 license.^[^ ^62^ ^]^ Copyright 2017, Nature Publishing Group.

For glassy materials, amorphous solids possess short‐ and medium‐range ordered structures with high entropy and free volume for motion. The ion transports initiate with excited local sites ions to the adjacent sites as collective diffusions for macroscopic scale consistent with crystals.^[^ ^59^ ^]^ Ion transfer follows the Arrhenius equation below the glass transition. In contrast, above the glass transition temperature (*T*_g_), the polymeric SEs involve the microscopic ionic transfer correlated to the segmental motion of polymer chains (**Figure** **2**a).^[^ ^60^ ^]^ Segmental motion generates the free volume to hop metal (Li, Na, K, Zn, Mg) ions that are coordinated to polar groups. Continuous hopping determines the long‐distance transfer kinetics under the applied electric fields (Figure 2b,c). The dissociation ability of metal salts in the polymeric chains provides the number of mobile ions.^[^ ^59^, ^60^ ^]^ The Vogel–Fulcher–Tammann (VFT) equation defines the correlation for ionic conductivity as a function of a temperature difference as:

(8) $$\sigma =\sigma_{o}/T\times \exp\left[\left(-Ea/R\left(T-T_{0}\right)\right.\right]$$

where *T_0_* the below *T_g_* (≈50K). *RT_o_* the potential barrier for a local motion of chains.^[^ ^61^ ^]^ Most of the polymer electrolytes follow the VFT kinetics.

FCC or HCP packing of anions shows a high‐energy landscape than BCC for various anion‐host matrices. The recognized fast Li^+^ conductors (Li_10_GeP_2_S_12_ (LGPS) and Li_3_OCl, Na^+^, Mg^2+^, and Ag+ ion conductors) consist of BCC anion matrices. MD simulations disclosed that LGPS, LLZO, NASICON, and Li_1.3_Al_0.3_Ti_1.7_(PO_4_)_3_ (LATP) have multiple ions hopping to adjacent sites instead of isolated ion hopping over energy barriers.^[^ ^62^ ^]^ Further, cubic LLZO shows 3D ion diffusion pathways with increased ion conductivity due to the redistribution of Li ions for LMBs. High‐molecular‐weight polymers enable intersegmental hopping, whereas low‐molecular‐weight polymers have diffusion mechanisms.^[^ ^60^ ^]^ Batteries contain several interfaces, such as homogeneous (grain boundary) and heterogenous (electrodes/electrolytes) forms, facilitating the vital ion‐transport role. LLZO or LISICON shows the grain boundary conductivity for charge transfer. The space‐charge layer is formed at the interface for heterogeneous interfaces due to the local transfer of uncompensated charges across the boundaries. This results in the accumulation of mobile carriers on the front side of the interface and depletion to the backside.^[^ ^63^, ^64^ ^]^ For example, inserting LiNbO_3_ buffer into LiCoO_2_ and LGPS electrolytes suppresses the growth of the space‐charge layer with excess ion‐transfer pathways.^[^ ^65^ ^]^

## 4 Evaluation of Structural Amendments during Metal Plating/Stripping

Nucleation of metal deposition, high surface‐area structures, and morphological disparity in stripping are major checkpoints for durable plating and stripping.

### 4.1 Nucleation Kinetics

**Figure 3:**
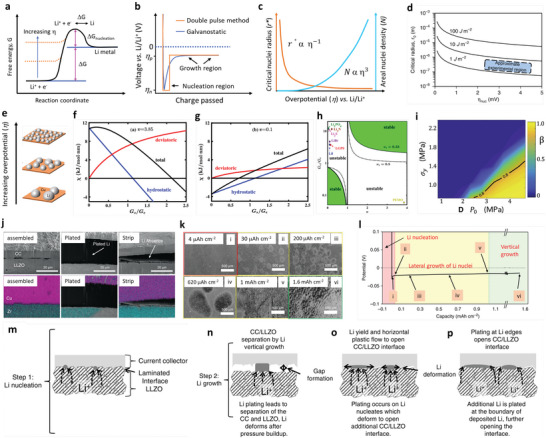
a) Free energy schematics for overpotentials versus nucleation energy barriers. b) Voltage profile schemes for galvanostatic (blue) and double phase (potentiostatic, orange) methods. c) The critical nuclei radius and areal nuclei density for the nucleation overpotentials. d) Critical radius versus nucleation overpotential. Reproduced with permission under Creative Commons CC BY 4.0 license.^[^ ^66^ ^]^ Copyright 2020, Nature Publishing Group. e) Metal nucleation schemes. Reproduced with permission.^[^ ^70^ ^]^ Copyright 2017, American Chemical Society. f,g) Stability parameters for ν >1 (f) and ν <1 (g). h) Stability phase diagram for shear modulus and volume ratios. Reproduced with permission.^[^ ^81^ ^]^ Copyright 2017, American Institute of Physics. i) Degree of plasticity (β) for Li relative to yield strength *σ_y_*. Reproduced with permission.^[^ ^67^ ^]^ Copyright 2020, Elsevier. j) SEM and EDS images of Li/LLZO interfaces for various conditions. k) LLZO SEs surface after plating for various capacities. l) Voltage profile displaying Li nucleation, lateral growth, and amalgamation of nuclei and their vertical growth. m‐p) Nucleation and growth process illustrations: Li nucleation (m), nuclei deposit leads to vertical growth and adjacent CC/LLZO interface separation (n), continuous electrodeposition over nuclei with mechanical forces at the interfaces, and lower Li yield leads the horizontal plastic flow for Li (o), deposition over nuclei edges (p).) Reproduced under the terms of a Creative Commons CC BY 4.0 license.^[^ ^66^ ^]^ Copyright 2020, Nature Publishing Group.

Nucleation kinetics critically depends on the current density, potentials, and SEI for solid‐state electrolytes. The nucleation barriers are effectively adjusted with varying electrochemical supersaturation by regulating reduction reaction overpotential at the working electrode for stable electrodeposition (**Figure** **3**a). Driving forces for electro‐crystallization processes are four types, including the reaction‐overpotential, charge‐transfer‐overpotential, crystallization‐overpotential, and diffusion‐overpotential. According to the limitation for the distinction of electrodes‐polarizations, the two typical overpotentials are considered for metal depositions: 1) nucleation‐overpotentials (*η*_n_, the value of voltage spike for the onset of M‐depositions; M = Li, Na, K, Zn, Mg, Al, Ca), and 2) plateau‐overpotentials (*η*_p_, the value after nucleation happens and M‐growth remains). During galvanostatic plating, the working electrode potential drops <0 V versus Li/Li^+^ to ‐*η*_n_ at electrochemical overpotential, which is appropriate for driving the nucleation of Li embryos (Figure 3b). After initiation, the overpotential increases to ‐*η*_p_ (negative values versus Li/Li^+^) and proceeds the growth of Li nuclei, even for lower electrode polarization for Li growth relative to those of nucleation. It ascribes to the favorable insertion of Li adatom with prevailing Li nuclei with lower energy barrier than those of evolving stable Li‐atoms cluster.^[^ ^66^, ^67^, ^68^, ^69^, ^70^ ^]^ For heterogeneous nucleation, the nuclei size is inversely proportional to the electrochemical overpotential, and areal nuclei density is proportional to the cube of the nucleation overpotential following the spherical nuclei (Figure 3c–e). The nucleation barrier and stable Li plateau enhance with a cumulative current density, consistent with the Butler‐Volmer relationship for current and voltages. Favorable conditions for Li deposit is *η*_p_<*η*_n_. Li domains are relatively large and meagerly distributed for low current densities (growth dominated), while small and dense distributions are for high current densities (nucleation dominated, Figure 3c). The excess charge is essential to complete the nucleation step for low current density due to the simultaneous deposition of Li and SEI formation.^[^ ^71^ ^]^ This feature is consistent with other metals such as Mg, Zn, Na, Al, K, and Ca.^[^ ^72^, ^73^, ^74^, ^75^, ^76^, ^77^ ^]^ Nucleation theory explains the critical radius at which growth is thermodynamically favorable and evaluated by Gibbs free energy of formed nuclei as:

(9) $$\Delta G_{\mathrm{total}}=4/3\times \pi r^{3}\Delta G_{\mathrm{Li}}+4\pi r^{2}W_{\mathrm{interface}}$$

where Δ*G_Li_* is the energy for Li depositions (or free energy change per volume), *W*_interface_ is the work for CC/Li/SEs interface (or surface energy). Upon consideration of boundary conditions and nucleation barriers, the critical radius becomes:

(10) $$r_{\mathrm{crit}}=\frac{2\gamma V_{M}}{F}\left|\eta \right|$$

where *γ* is surface energy, *V_M_* is molar volume, *F* is the Faraday constant, and *η* is nucleation overpotential. For fixed plating capacity, the nuclei density *N* times the nuclei volume should be constant. Thus, nuclei density is *N* ∼ *η* ^3^/γ^3^ (Figure 3c).

Critical radius is the boundary conditions for Li nuclei formation; below *r*_crit_, no Li metal nuclei can be generated. Figure 3d explains a few mV overpotentials (*η*) required as driving forces to generate first Li nuclei for diffusion‐bonded CC/Li/SEs interfaces. When nuclei undergo the applied pressure, it will further increase the potential driving forces obligatory for driving the nucleation and growth processes. Nucleation overpotential is defined as:

(11) $$h\approx \left(V_{Li}/F\right)\times p$$

where *p* is the pressure in the nuclei. Reported critical radius for Li, Mg, Zn, Na, and K are in 1–6 µm range. For instance, an applied pressure of 4 MPa further requires *η* of 0.5 mV.^[^ ^66^ ^]^ Motoyama et al.^[^ ^69^ ^]^ also reported that Li‐plating for CC/LiPON SEs interfaces have voltage rises of 30 and 50 mV for 90 and 30 nm CC, respectively, but <10 mV for 1 µm CC with a fixed strain of 0.2%. This theory confirms that surface and interfacial energies are critical parameters for Li plating. Biswal et al.^[^ ^78^ ^]^ verified the composition of SEI strongly influences the effective surface energy of Li metal. Morphology and distribution of Li electrodeposition concerning plated capacity propose the interfacial forces define the nucleation and flat growth of isolated Li to the uniform coalesced layer.^[^ ^66^, ^68^, ^69^ ^]^ MD simulations reported that Li reduces preferentially with homogeneous growth at the LiF SEI cracks, highlighting the surface tension and mechanical properties strongly influencing Li nucleation.^[^ ^79^ ^]^ Butler‐Volmer equation defines the change in the electrochemical potential of electrons for interfaces based on surface tension and interfacial stresses as:^[^ ^80^, ^81^, ^82^, ^83^ ^]^

(12) $$\begin{array}{ccc} \Delta \mu_{e-} & = & -1/2z\times \left(V_{M}+V_{M}^{Z+}\right)\times \left[-\gamma \kappa +e_{n}^{2}\left(\tau_{d}^{e}-\tau_{d}^{s}\right)\right]\\ & & +\;1/2z\left(V_{M}-V_{M}^{Z+}\right)\left(\Delta p^{e}+\Delta p^{s}\right) \end{array}$$

where Δ*p^e^* and Δ*p^s^* as well as *τ_d_^e^* and *τ_d_^s^* are the pressures and deviatoric stresses at electrodes and electrolytes of interfaces, *e_n_* the unit normal pointing towards SEs from metal, *κ* the mean curvature at the interface, and *V*_M_^Z+^ the molar volume of *M^Z+^* in SEs. After boundary conditions (i.e., bulk force = 0) and constitutive laws for linearly elastic isotropic materials with the shear modulus (*G*) and Poisson's ratio (ν), the Δµ*_e−_* for interface becomes:^[^ ^80^, ^81^, ^82^, ^83^ ^]^

(13) Δμe−=χ×ReAeikxwithχGe,Gs,νe,νs,γ,k,z,VM,VMZ+

where χ the stability factor includes surface tension, hydrostatic, and deviatoric stresses. If the current density and Δµ*_e−_* are out of phase with perturbation, then stable deposition is possible. Figure 3f,g shows hydrostatic contributions are initially positive and decrease monotonically with *G_s_*/*G_e,_* which results in stability for *G_s_*/*G_e_* ≥ 2.2, whereas hydrostatic stresses are initially negative and increase stability for *G_s_*/*G_e_* ≤ 0.7. For *ν* >1, the electrodeposition is stable beyond the critical shear modulus ratio (χ < 0), whereas, for *ν* < 1, stable deposition is possible below critical shear modulus (χ > 0). Figure 3h exhibits the stability phase diagram for a shear modulus and molar volume ratio. For *ν* >1, stable deposition is possible when SEs with shear modulus >> critical shear modulus. The required shear modulus enhances severely with the unity of molar volume ratio, reducing the stability window. For *ν* < 1, stable deposition is possible with soft SEs, provided Li in SEs is more densely‐packed than Li in Li‐metals (i.e., density‐driven stability). For *ν* = 1, stability requires hydrostatic stresses to dominate as stability parameters (i.e., pressure‐driven stability). The stress‐driven phase transitions at solid‐solid interfaces, the interplay among the work and elastic energy terms defines the growth and stability of interfaces.^[^ ^80^, ^81^ ^]^ Small exchange current density via SEI increases the compatibility of deposition. Further, organic fluorine with flexible polymeric elements in SEI strongly prevents the dissolution of dead Li by constructing the condensed SEI, which illustrates the energetic LiF with oligomers and polymers stabilizes the Li anode.

NASICON‐type Na_3_Zr_2_Si_2_PO_12_ (NZSP) with monoclinic and rhombohedral phases displays the 3D Na‐ion transmission channel. The Na^+^ diffusion is faster with large ion transport pathways due to the lower electronegativity and sizeable ionic radius of the S atoms in the sulfide electrolytes, which significantly decreases the grain boundary resistances. Besides, the distribution of Na^+^ nuclei is more homogeneous for lower concentrations. Overpotential from the mass transfer is higher for low Na^+^ concentrations, enabling excess nuclei formation. The excess Na^+^ ions show more pronounced nuclei merging with close‐packed nuclei.^[^ ^72^, ^73^, ^74^ ^]^ SEI formed for Zn has a similar influence on alkali metals for lower current regions. Zn crystal facet displays the high anisotropy ensuing the hexagonal close‐packed lattices for a c/a ratio of 1.85, which enforces major impacts over growth kinetics. The (002) texture accelerates the Zn nucleation rate with abundant nuclei for higher currents, which enables stable deposition and high resistance to dendrite growth and interfacial reactions. During electrolysis, the negative electrodes are surrounded by Zn^2+^ and H^+^ ions with weak Van der Waals bond that allows favorable surface diffusion with Zn^0^ deposits over the Cu plate.^[^ ^75^ ^]^ Voltage profiles with 1.6NH_3_@MgO composites display stable interfaces (low overpotentials) for low currents, while imbalanced kinetics are followed for higher currents. This high overpotential for nucleation compared to the growth indicates that Mg electrodeposition follows the instantaneous nucleation process ascribed to the Gaussian size dispersion.^[^ ^76^ ^]^

Zhu et al.^[^ ^84^ ^]^ reports the Na nucleation behavior with various group‐II metal‐foils (Be, Mg, Ba) with definite solubility in Na as substrates for Na plating compared to those of Al and Cu foils. It exhibits low overpotentials of 36.3, 35.5, and 12.1 mV for Be, Mg, and Ba relative to Al (53 mV) and Cu (44.9 mV) foils, implying smaller nucleation barriers for Na‐deposits. Flat and compact Na‐loadings over group‐II metals are shown with homogeneous nucleation in spheres and domes, whereas Al and Cu show non‐uniform heterogeneous nucleation with plenty of dendrites. Further, the infinite solubility of group‐II metals enables surface metallic atoms dissolution in the Na before producing pure Na‐phase causing solid‐solution layers. Liu et al.^[^ ^85^ ^]^ states the current density or areal capacity changes the nucleation overpotential for highly‐ordered CNTs, which is 1/3 of graphene and Cu electrodes. Homogeneous electric fields and low binding energy of highly‐ordered CNTs gather massive K‐deposition of graphene current‐collector than Cu‐electrodes. Liu et al.^[^ ^86^ ^]^ reports the epitaxial electrocrystallization of Mg over 3D magnesiophilic substrates by interactions of lattice mismatch, electrostatic confinements, and magnesiophilic interfaces. Ni(OH)_2_ substrates display strong magnesiophilic characteristics and lower lattice misfit for Mg, implying the condition for heteroepitaxial Mg nucleation. It shows periodic and hillock‐like electrostatic fields on the exposed surfaces that precisely confine the reduced Mg^0^ over the localized electron‐enriched atomic‐level sites. Ni(OH)_2_@CC undergoes uniform Mg‐deposition and electrocrystallization with locked crystal reflections and improved plate/strip kinetics. Hu et al.^[^ ^87^ ^]^ reports insertion of Mg(BH)_4_ in the magnesium bis(trifluoromethanesulfonyl)imide (Mg‐(TFSI)_2_) increases the Mh‐nucleation sites that are closely packed for electrodes at initial stages of depositions and then rapid growth of large clusters. McClary et al.^[^ ^88^ ^]^ reports heterogeneous ultrathin CaO stabilizes the Ca^2+^ plate/strip processes efficiently rather than CaH_2_ with 5–10 nm SEI thickness. The structural and compositional heterogeneities enable Ca^2+^‐conduction via CaO films similar to Li^+^‐conduction models through SEI. Boundaries among the neighboring CaO crystals and minor phases of CaO control the lower migration barriers with superior transport‐pathways.

### 4.2 Growth Morphology

Generally, the electrodeposition shows the horizontal, vertical, and randomly oriented growth structures (such as fractal‐like micron‐scaled dendrites, chemotaxis‐like, mossy‐like whiskers, and wires) for different electrolytes under applied current densities (above/below their critical limits). Dendrites (fractal‐like) with tip growth are commonly observed for all metals of Zn, Li, Mg, Al, and K due to the electrolyte transport limits.^[^ ^72^, ^73^, ^74^, ^75^, ^76^, ^77^ ^]^ The concentration of ions for anodes is depleted for above critical current density at Sand's time.^[^ ^89^ ^]^ Diffusion limitations, chemical instability, and physical orphaning describe the metal's propensity for undergoing side reactions with porous, heterogeneous, and dendrite morphology, leading to the plating/stripping efficiency of 50–90%. This non‐compact porous electrodeposition shows volume expansion over the anode surface. Zheng and Archer reported^[^ ^75^ ^]^ the heterogeneous Zn deposition exhibits a high modulus of 108 GPa compared to those of Li (5 Gpa) and Na (10 GPa), and it penetrates separators/membranes while making a physical bridge of electrode, defining as cell failure.^[^ ^35^, ^90^, ^91^ ^]^ Reversibility process influences two major parameters of geometry and assembly modes of microstructural building blocks. The geometry describes the dimension and symmetries of microstructural building blocks [1D (Φ = 1 µm), 2D (Φ = 5 µm)]; however, the assembly modes describe the orientations/alignments of building blocks relating to electrode surfaces. Plating/stripping reversibility of different morphologies as horizontally aligned (≈99%) > non‐aligned ≈ randomly oriented wires/moss‐like (80−90%) > vertically aligned dendrites (<50%).^[^ ^91^, ^92^ ^]^ In contrast to Li, poor reactivity of Zn validates a substantial amount of Coulombic inefficiency arises due to the loss of active materials via parasitic reactions with SEs, illustrating the failure for Zn is severely relates to the loss of electrochemical accesses for depositions. Figure 3j displays non‐uniform growth of the intermediate phase upon plating, whereas it disappears upon stripping with retention of the gap, increasing the impedance. Figure 3k,l exhibits the surface morphology distribution with increased plating capacities in nucleation, lateral growth of Li nuclei, and vertical growth of Li film regions. Nucleation and growth kinetics with subsequent processes, including nuclei initiation, vertical growth of Li columns, the horizontal plastic flow of Li for new LLZO/CC interface, plastic deformation under compression, or direct plating of Li over LLZO/CC interface have been shown in Figure 3m–p.

Mossy growth of metal consists of intertwined nano‐sized whiskers without specific direction following the root growth. The intrinsic electrochemical instability of Li (or others) with electrodes leads to SEI generation. Slower ion transfer via SEI facilitates the poor exchange current density with microscopic reacted‐limited growth. The buds will grow in all directions for low overpotentials, in contrast to the root‐growth‐based whiskers for high overpotentials. First, nuclei will grow in the square root of time, then be pushed away from the root with rapid growth in length; after that growth rate reduces (Figure 1i). Typically, SEI plays a vital role in the growth mechanism. SEI will grow slowly with rapid Li‐ions transfer from thin SEI for small overpotential. This metal deposition is covered by a thin SEI layer without mechanical resistance. On the other hand, SEI forms quickly for high overpotentials with thick SEI covered on the electrode surface with eventual whiskers. Favorable growth depends on operating conditions, electrolytes, and current collectors.^[^ ^93^ ^]^ Surface inhomogeneity causes the local amplification of von Mises stress (*σ_v_*) and hydrostatic pressure (*P*) for the interfaces. Ceder et al.^[^ ^67^ ^]^ reports the distribution of *P* and *σ_v_* for Li at the interface for yield strength (σ_y_ = 0.8 MPa) and various stack pressure (*P_0_*). As the P increases, the *P_0_* also increases, reaching a maximum of 5.8 MPa value. Interfacial *σ_v_* also enhances; however, it bounds with a yield strength (σ_y_ = 0.8 MPa), illustrating surface irregularity has maximum *σ_v_* with plasticity. Further, the application of large *P_0_* exhibits the growth of plastic regions from the peak area (Figure 3i). Conclusively, stress‐gradient, self‐diffusion, and limited surface diffusion describe the growth mechanism.

Theoretical calculations explained that Mg has a more considerable free energy difference for low and high dimensional phases than Li, illustrating the preferred 2D/3D growth relative to 1D Li growth. However, several reports vindicated the dendrites growth of Mg anode for all electrolytes.^[^ ^76^, ^94^ ^]^ Yu et al.^[^ ^72^ ^]^ reported Na‐dendrites with PEO/NaTFSI electrolytes; however, insertion of Na_3_SbS_4_ filler forms the NaF‐based SEI for smooth growth. The role of anions for solvation critically impacts the SEI compositions. Na/NASICON/Na shows rapid dendritic growth and diffusion of grain boundaries, consistent with Li/LLZO/Li. Further, an increase in the local electric field during charging tends to plate Na on grain boundaries; however, thinner dendrites are possible due to narrow grain boundary edges that facilitate the realization of more dendrites.^[^ ^95^, ^96^ ^]^

Kim et al.^[^ ^97^ ^]^ reports in situ NaF‐rich protective layers over Na‐metal surface as SEI that prevents the depletion of the electrolyte during cycling. Na//Na cells display lower overpotentials of 8, 50, and 70 mV for 1, 5, and 10 mA cm^−2^ current densities without dendritic growth. Smaller interface resistance is attributed to the ameliorated ion conductance and smaller diffusion barriers for Na‐ions across the interfacial layer. Wei et al.^[^ ^98^ ^]^ presents Ga_5_Mg_2_ alloys as Mg^2+^‐conductive, corrosive resistant, and magnesiophilic Mg‐anodes. The lower chemical reactivity of Ga_5_Mg_2_ than Mg‐foils displays a protective layer for interfacial corrosion over reversible Mg plate/strip. Theoretical results confirm the lower diffusion barriers of 1.91 and 2.55 eV for intra‐ and cross‐layers diffusions implying the faster Mg^2+^‐diffusion kinetics. Uniform and dendrite‐free Mg‐plating is obtained for Ga_5_Mg_2_‐Mg over long operations, whereas Mg‐foils display dendrites even after 10 cycles, which verifies the inhibition of Mg‐dendrites. Ma et al.^[^ ^99^ ^]^ reports the electroplated Zn over carbon nanofibers as anodes with large surface area and enhanced permeation of electrolytes. Liu et al.^[^ ^100^ ^]^ reports the nucleation and growth behaviors of Zn over the stainless‐steel hosts. It shows hexagonal‐like layered flakes at different applied current densities and areal capacities for various dimensions. Distribution of Zn flake size decreases with increased current densities for all areal capacities, manifesting uniform depositions even for higher current densities. Zn has two overpotentials: 1) Initial sharp voltage downhill spike corresponds to nucleation overpotentials that drive Zn embryos nucleation, 2) Plateau potentials are the growth overpotentials that show Zn‐growth energy barriers. Nucleation and growth overpotentials increased from 86 and 42 mV at 0.25 mA cm^−2^ to 396 and 220 mV at 20 mA cm^−2^, illustrating the realization of numerous nuclei over the substrates. Tian et al.^[^ ^101^ ^]^ reports 3D Zn‐Cu alloy (Zn_5_Cu) interface materials for dual‐cation electrolytes (Zn^2+^/Mg^2+^ and Zn^2+^/Na^+^). Thin ZnO surface engineering significantly enhances plate/strip kinetics for low and high current densities.

### 4.3 Formation of Dead Metals and Approaches for 2D Plating

Generally, using metal anodes for commercialization is limited by porous and mossy metal growth for long‐term cycling. Structural deficiencies in the anode show capacity loss due to the inflated surface area and evolution of inactive metal through stripping. SEI formation is a fundamental parameter for displaying severe capacity degradation at high CEs. The inactive metals (Li, Na, Zn, Mg, Al, or Ca), generally denoted as “dead metal,” are the controlling parameter around CEs <95%.^[^ ^92^ ^]^ For all metal anodes, the plating/stripping rates are the determining factors for evolving metal morphologies during cycling.^[^ ^72^, ^73^, ^74^, ^75^, ^76^, ^77^ ^]^

For different metal anodes, it is necessary to determine the compatible approaches for the realization of 2D plating with minimal structural disorders and CEs of 99.9% for realistic metal batteries. For instance, the 3D electrode design is one of the alternate approaches to mitigating the shape change and reactivation of dead Li. Pulse charging, high current densities, and lower salt concentrations are suitable for compensating diffusion kinetics. Plating/stripping current densities influence overpotentials and reversibility reaction kinetics. The stable SEI regulates the nucleation and growth kinetics by minimizing the internal stress to realize flat 2D plating.

Fang et al.^[^ ^92^ ^]^ reported total inactive Li is equivalent to capacity fade for plate/strip, illustrating linear relation with CEs. The quantity of SEI Li^+^ can be determined as Total inactive Li (known) = metallic Li (unreacted, measured) + SEI Li^+^. Notably, the metallic unreacted Li^0^ enhances significantly with reducing CEs, whereas SEI Li^+^ retains lower values for all testing conditions. The morphology of inactive Li changes from sheet‐like to clusters with increased stripping rates for highly concentrated electrolytes, in contrast to whisker‐like Li depositions with thick inactive Li under conventional electrolytes. Note that residues display a loss of electrically conductive pathways. Mohammad et al.^[^ ^77^ ^]^ argued that the electroplating of Al metal composed of Al crystallites with ionic polymer electrolytes fulfills the basic compatibility need. Guo et al.^[^ ^102^ ^]^ reported the Mg^2+^ shows stable depositions under solid electrolytes (NASICON, oxides, hydrides, and chalcogenides) by using various strategies such as doping, binary or ternary phased SEs, amorphous or single crystalline phase structures with high thermal stability, and increased anions volume, etc. Na metal and ceramic SE effectively minimize the interfacial resistances and dendrites by improving the wetting of interlayer (i.e., uniform sodium flux across interface).^[^ ^95^ ^]^ The following sections will consider macroscopic reactions restriction directed by the transport of metal ions via SEI, and the interface structures vital for stable operations of various metal anodes chemistries with practical requirements.

Landmann et al.^[^ ^74^ ^]^ reports plate/strip behavior of Na for high‐temperatures carbon‐coated ceramic Na‐β"‐alumina electrolyte. It shows a plate/strip for a high current density of 2600 mA cm^−2^ with a cumulative capacity of 10 Ah cm^−2^ at 250 °C without dendritic‐growth. Wang et al.^[^ ^103^ ^]^ reports MoO_2_ nanocrystals@N‐doped carbon nanofibers self‐supporting anode under polyvinylidene fluoride hexafluoropropylene‐based Na^+^‐electrolyte, in which reduction peaks of 1.38 and 0.9 V relates to SEI formation without nay oxidation peaks, illustrating insertion/desertion of Na^+^ over 0.01−1 V potential‐range. After 2000 cycles, MoO_2_ NCs@N‐CNFs possess 1D‐ordered networks with sodiate/desodiate MoO_2_ (−121) reflections that manifests rational Na^+^‐storage kinetics. MoO_2_ subnanoclusters encapsulated in nitrogen‐doped carbon nanofibers as anodes are also reported for Na^+^‐storage with PMMA SEs.^[^ ^104^ ^]^ Qin et al.^[^ ^105^ ^]^ reports Na‐cycling via polydopamine/multilayer graphene‐based polypropylene (mPG‐12@PP) for stable operations of 2000 h at 4 mV overpotential. Under polypropylene, Na‐dendrites with loosely moss‐like structures are observed at 1 mAh cm^−2^, whereas mPG‐12@PP provides dense and smooth Na‐depositions without dendrites even at 2 mAh cm^−2^, which confirms the key role of mPG‐12 toward control for Na^+^‐plate/strip. Cohn et al.^[^ ^106^ ^]^ reports efficient plating/stripping for Na‐metal for 4 mA cm^−2^ with Na‐loading up to 12 mAh cm^−2^, and long operations for 1000 cycles at 0.5 mA cm^−2^. The carbon layer reduces the overpotential from 19 to 12 mV for plating and improved performance attributed to the large surface area of carbon, reactive sp^3^‐carbon sites and oxygen‐comprising functional groups, and initial storage of Na^+^‐ions in C‐framework. Wu et al.^[^ ^107^ ^]^ reports potassium bis(fluoroslufonyl)imide (KFSI)‐dimethoxyethane (DME) based electrolytes generate reversible K^+^‐plating/stripping with SEI and 99% CEs. It shows ordered surface morphology preventing parasitic reactions and maintaining dendrite‐free K^+^‐plate/strip upon cycling. XPS verifies the ─SO_2_^−^ species due to FSI^−^‐anions depletion, S‐F bond breakage, and S═O/C═O species as SEI compositions. Theoretical calculations demonstrate the requirements for Mg‐metal anodes regarding mobility, electronic band gaps, and stability by analyzing 27 binary, ternary, and quaternary compounds for wider‐chemical space. MgSiN_2_, MgI_2_, MgBr_2_, MgSe, and MgS are identified as potential materials against the highly reductive Mg‐anodes, and MgAl_2_O_4_ and Mg(PO_3_)_2_ are promising for high‐voltage cathodes (≈3 V).^[^ ^108^ ^]^ Bae et al.^[^ ^109^ ^]^ reports amorphous MgO‐coated Zn‐framework as CCs for an anode‐free Mg battery to allow reversible Mg^2+^ plating/stripping in oxidatively stable electrolytes. The lattice mismatch among the MgO and Zn persuades the disorders, manifesting a defective interface of amorphous MgO + Mg_x_O_y_ + Mg with a mixed ionic–electronic conductor. Dueramae et al.^[^ ^110^ ^]^ reports the compatibility of carboxymethyl cellulose electrolytes with the Zn‐salt complex (GPE_A_15). Zn//Zn cells display larger voltage fluctuations from 0.5−10 mA cm^−2^ current densities reflecting the high internal resistance of cells that impedes charge accumulation over the boundaries and poor interfaces with electrodes. Larger overpotential implies poor Zn^2+^‐nucleation, attributed to thicker SEI and the accumulation of electronically detached/dead Li fibrils over interfaces.^[^ ^111^ ^]^

## 5 Solid‐State Electrolytes (SEs)

With the limited resources and uneven distribution of Li, there is a revival of interest in other batteries, including Na, K, Mg, Zn, and Al. Notably, ion conductivity is the critical parameter of SEs. However, commercial electrochemical conversion or storage systems require several major properties, such as super‐ionic conductivity, high ion‐selectivity, excellent chemical and electrochemical compatibility, broad electrochemical window, scalable and facile preparation processes, superior electronic area‐specific resistance, smaller ionic area‐specific resistance, excellent thermal stability and mechanical properties, cost‐effective, superior device adaptation, and environmental friendliness.^[^ ^10^, ^11^, ^12^, ^112^, ^113^, ^114^, ^115^, ^116^, ^117^, ^118^, ^119^, ^120^, ^121^, ^122^, ^123^, ^124^, ^125^, ^126^, ^127^, ^128^ ^]^ Extensive efforts have been reported for organic and inorganic SEs. **Table** **1** provides the state‐of‐the‐art multivalent SEs and their properties.^[^ ^10^, ^11^, ^12^, ^32^, ^39^, ^102^, ^112^, ^113^, ^114^, ^115^, ^116^, ^117^, ^118^, ^119^, ^120^, ^121^, ^122^, ^123^, ^124^, ^125^, ^126^, ^127^, ^128^, ^129^, ^130^, ^131^, ^132^, ^133^, ^134^, ^135^, ^136^, ^137^, ^138^, ^139^, ^140^, ^141^, ^142^, ^143^, ^144^, ^145^, ^146^, ^147^, ^148^, ^149^, ^150^, ^151^, ^152^, ^153^, ^154^, ^155^, ^156^, ^157^, ^158^, ^159^, ^160^, ^161^, ^162^, ^163^, ^164^, ^165^, ^166^, ^167^, ^168^, ^169^, ^170^, ^171^, ^172^, ^173^, ^174^, ^175^, ^176^, ^177^, ^178^, ^179^, ^180^, ^181^, ^182^, ^183^, ^184^, ^185^, ^186^, ^187^, ^188^, ^189^, ^190^, ^191^, ^192^, ^193^, ^194^, ^195^, ^196^, ^197^, ^198^, ^199^, ^200^, ^201^, ^202^, ^203^, ^204^, ^205^, ^206^, ^207^, ^208^, ^209^, ^210^, ^211^, ^212^, ^213^, ^214^, ^215^, ^216^, ^217^, ^218^, ^219^, ^220^, ^221^, ^222^, ^223^, ^224^, ^225^, ^226^, ^227^, ^228^, ^229^, ^230^, ^231^, ^232^ ^]^

**Table 1:** State‐of‐the‐art solid electrolytes.^[^ ^10^, ^11^, ^12^, ^32^, ^39^, ^102^, ^112^, ^113^, ^114^, ^115^, ^116^, ^117^, ^118^, ^119^, ^120^, ^121^, ^122^, ^123^, ^124^, ^125^, ^126^, ^127^, ^128^, ^129^, ^130^, ^131^, ^132^, ^133^, ^134^, ^135^, ^136^, ^137^, ^138^, ^139^, ^140^, ^141^, ^142^, ^143^, ^144^, ^145^, ^146^, ^147^, ^148^, ^149^, ^150^, ^151^, ^152^, ^153^, ^154^, ^155^, ^156^, ^157^, ^158^, ^159^, ^160^, ^161^, ^162^, ^163^, ^164^, ^165^, ^166^, ^167^, ^168^, ^169^, ^170^, ^171^, ^172^, ^173^, ^174^, ^175^, ^176^, ^177^, ^178^, ^179^, ^180^, ^181^, ^182^, ^183^, ^184^, ^185^, ^186^, ^187^, ^188^, ^189^, ^190^, ^191^, ^192^, ^193^, ^194^, ^195^, ^196^, ^197^, ^198^, ^199^, ^200^, ^201^, ^202^, ^203^, ^204^, ^205^, ^206^, ^207^, ^208^, ^209^, ^210^, ^211^, ^212^, ^213^, ^214^, ^215^, ^216^, ^217^, ^218^, ^219^, ^220^, ^221^, ^222^, ^223^, ^224^, ^225^, ^226^, ^227^, ^228^, ^229^, ^230^, ^231^, ^232^ ^]^

Metal ions	Materials	Ion conductivity [S cm^−1^]	Activation energy [eV]	Remarks
**Sulfides**				
Li^+^	Li_2_S‐P_2_S_5_, Li_2_S‐P_2_S_5_‐MS_x_, LGPS, Li_3_PS_4_, Li_7_P_3_S_11_, Li_3_SbS_4_ (glass), Li_9.54_Si_1.74_P_1.44_S_11.7_Cl_0.3_, Li_7_P_3_Se_11_, Li_6_PS_5_Br, Li_3.833_Sn_0.833_As_0.166_S_4_, Li_7‐x_PS_6‐x_Cl_x_, Li_6_PS_5_Cl, Li_4_SnS_4_,	10^−7^–10^−3^	0.1–0.25	Superion conductivity, Great mechanical properties, Lower grain boundaries resistances, Sensitive to moisture, Superior ion selectivity, Low chemical stability
Na^+^	Na_3_PS_4_ (cubic), Na_7_P_3_S_11_, Na_7_P_3_Se, Na_3_SbS_4_, Na_2.9375_PS_3.9375_Cl_0.0625_, Na_3_P_0.62_As_0.38_S_4_, Na_2.88_Sb_0.88_W_0.12_S_4_, Na_4−x_Sn_1−x_Sb_x_S_4_, Na_5−2x_Al_1 −x_V_x_S_4_, Na_5−2x_Al_1−x_Ta_x_S_4_, Na_5−2x_In_1−x_Sb_x_S_4_	10^−5^–10^−2^	0.2–0.3	Poor oxidation stability, High‐ion conductivity, Poor chemical, oxidation, and reduction stability
Mg^2+^	MgS‐P_2_S_5_‐MgI_2_, MgSc_2_Se_4_, MgSc_1.9_Ti_0.075_Se_4_, MgLn_2_X_4_ (Ln = Lu, Tm, Er, Ho, Dy, Tb, Sm, Pm, Nd, Pr, La, Y, Ce, Gd, Yb and X = S, Se), MgIn_2_S_4_, MgSc_2_Se_4_, MgY_2_Se_4_, MgY_2_S_4_, MgIn_2_Se_4_, MgSc_2_Te_4_, and MgY_2_Te_4_	10^−6^–10^−4^	–	Poor ion‐conductivity, High mechanical strength, Poor compatibility with cathodes, Low flexibility and high cost Chemical and structurally unstable, High electronic conductivity
Zn^2+^	ZnPS_3_, Zn_y_S_1‐x_F_x_, ZnSc_2_S_4_, ZnY_2_S_4_, ZnIn_2_S_4_, ZnY_2_Se_4_, ZnSc_2_Se_4_, ZnIn_2_Se_4_,	10^−9^–10^−3^	0.3–0.4	Poor conductivity and oxidation stability, Limited thermal stability, Poor interface compatibility, Excess grain boundaries
**Halides**				
Li^+^	Lil, Li_2_ZnI_4_, Li_3_N, Li_3_OCl, Li_3_MCl_6_ (M = Y, In, Sc, Ho), Li_0.388_Ta_0.238_La_0.475_Cl_3_, ZrO_2_(‐LiCl)‐Li_2_ZrCl_6_, ZrO_2_−2Li_2_ZrCl_5_F, Li_3_MX_6_ (X = Cl, Br)	10^−8^–10^−3^	–	Stable with Li metal, Good mechanical properties, Poor oxidation voltage and conductivity, Moisture sensitive, Poor chemical and thermal stability, Excellent reduction stability, ion selectivity, High processing cost
Na^+^	NaAlCl_4_, Na_3_MX_6_ (X = Cl, Br, I), ZrO_2_(‐NaCl)‐Na_2_ZrCl_6_, Na_3–x_Er_1–x_Zr_x_Cl_6_, Na_3–x_Y_1–x_Zr_x_Cl_6_, NaAlCl_4_	10^−6^–10^−4^	–	High processing cost, Poor ion conductivity and chemical stability, Poor thermal stability
**Oxides**				
Li^+^	Li_7_P_3_O_11_, Li_7_La_3_Zr_2_O_12_ garnet, Perovskites, Li_3.3_La_0.56_TiO_3_, Li_2_Ti(PO_4_)_3_, Li_14_Zn(GeO_4_)_4_, Li_6.5_La_3_Zr_1.75_Te_0.25_O_12_, Li_7.06_M_3_Y_0.06_Zr_1.94_O_12_ (M = La, Nb or Ta), Li_6_ALa_2_M_2_O_12_ (A = Ca, Sr or Ba; M = Nb or Ta), (Li_3_M_2_Ln_3_O_12_ (M = W or Te), Li_5.5_La_3_M_1.75_B_0.25_O_12_ (M = Nb or Ta; B = In or Zr)	10^−5^–10^−3^	0.3–0.5	High chemical and electrochemical stability, High electrochemical oxidation voltage, High oxidation and thermal stability, Low processing cost and mechanical properties
Na^+^	Na_7_P_3_O_11_, NaTi_2_(PO_4_)_3_, Na_3_Zr_2_Si_2_PO_12_, Na_2.8_Zr_2_Si_1.8_P_1.2_O_12_, Na_3.2_Zr_2_Si_2.2_P_0.8_O_12_	10^−6^–10^−4^	0.5–0.8	Good electrochemical stability, Non‐flexible, Poor device integration
Mg^2+^	Mg_0.5_Zr_2_(PO_4_)_3_, Mg_0.5_Ti_2_(PO_4_)_3_, MgHf(WO_4_)_3_, Mg_0.5_Zr_2_(AsO_4_)_3_(PO_4_)_3_, Mg_0.5_Ce_0.2_Zr_1.8_(PO_4_)_3_, MgZr_4_P_4_O_24_, Zr_2_O(PO_4_)_2_	10^−7^–10^−4^ (500–600 °C)	–	Good electrochemical oxidation voltage, High mechanical strength, Expensive for large‐scale, High thermal stability and safety
K^+^	K_5_As_3_O_10_, K_4_V_2_O_7_, K_2_Zn_3_O_4_, K_2_Sb_4_O_11_, K_3_NbAs_2_O_9_, K_3_NbP_6_, K_2_Al_2_Sb_2_O_7_, K_2_Fe_4_O_7_, K‐BASE	10^−5^–10^2^	0.2–0.6	High ion conductivity, Poor chemical compatibility, Poor device integration
Zn^2+^	Zn silicate, Bi_2_Zn_0.1_V_0.9_O_5.35_, ZIF‐8, ZnMOF‐808	10^−6^–10^−3^	0.15–0.5	Poor chemical and electrochemical stability, High cost, Poor device integration, Poor thermal stability
Ca^2+^	β″‐Al_2_O_3_	10^−4^–10^−2^ (300 °C)	–	Poor device integration, operations only for high temperatures, high interface resistance
**Polymers**				
Li^+^	PEO, PVDF, LiTFSI‐PEO, LiClO_4_‐PEO, LiClO_4_‐PEO, (10:1, 20:1, 30:1) Mw = 600k‐5000k, PVDF‐LLZO, PAN‐LiClO_4_, PEO‐LTO,PEGMEA‐LLZO, PAN‐LiTFSI, PAM‐LiTFSI, PPA‐LiTFSI, PSA‐LiTFSI	10^−8^–10^−4^	0.4–1.0	Flexible and low conductivity Low shear modulus, Scalable for large‐area, Stable with Li‐metal, High processing cost, Poor thermal, oxidation, reduction, and chemical stability
Na^+^	PEO, NaTFSI‐PEO, NaClO_4_‐PEO, NaClO_4_‐PEO with 5 wt% TiO_2_, (10, 20, 30:1) Mw = 600k‐5000k, PEO‐NaPF_6_, NaSCN, PEO‐NSS, PEO‐NASICON, PEO‐NPSO	10^−9^–10^−4^	–	Limited thermal and chemical stability, Low oxidation voltage, Poor ion conductivity
Mg^2+^	MgCl_2_‐PEO_16_, PVA‐PEG‐Mg(NO_3_)_2_, Mg(ClO_4_)_2_‐PVDF‐HFP‐TiO_2_, Mg(AlCl_2_‐EtBu)_2_‐PEO, Mg(AlCl_2_‐EtBu)_2_‐PVDF, Mg(TFSI)_2_ or Mg(Tf)_2_ with PEO, PAN or PVP	10^−7^–10^−6^	–	Poor ion conductivity and electrochemical stability, High interfacial resistance, Fine interfacial contact, Limited thermal and mechanical stability, High flexibility, Facile manufacturing process
Zn^2+^	CNF‐PAM‐Zn(CF_3_SO_3_)_2_, ZnTFSI‐PEO, ZnSO_4_‐PEO, Zn‐Alginate/PAM, Gelatine, CMC, Gelatin‐g‐PAM, Xantum, PVA, and PPA with Zn‐salts	10^−9^–10^−5^ (SPEs) 10^−6^–10^−3^ (GPEs)	0.5–1.8 (SPEs) 0.3–0.8 (GPEs)	Prohibit Zn dendrites growth and corrosion, rapid degradation, Flexible and compressible, Operate for subzero temperatures, Extremely large swelling
K^+^	PPC‐KFSA, PMMA‐KPF_6_, PMMA‐KFSA, PEO‐KFSA, PA‐KPF_6_, PEO‐KFSI, PPC‐KFSI,	10^−8^–10^−5^	–	Poor ion conductivity, Poor thermal, chemical, and oxidation stability
Al^3+^	1‐ethyl‐3‐methylimidazolium chloride–AlCl_3_, AlCl_3_‐PA‐Et_3_NHCl, IL‐AlCl_3_	10^−9^–10^−3^	–	Poor ion conductivity, Poor thermal, chemical, and oxidation stability
Ca^2+^	PEGDA‐Ca‐salts (nitrate, TFSI, FSI, BF_4_, ClO_4_), PTHF‐Epoxy‐Ca(NO_3_)_2_, PEGDA‐Ca(BF_4_)_2_, EO/Ca,	10^−9^–10^−4^	0.22–0.8	Poor chemical and thermal stability, Poor ion conductance, High interface resistance, Poor EWs
**Hydrides**				
Li^+^	LiBH_4_, LiBH_4_‐LiX (X = Cl, Br, or I), LiNH_2_, LiBH_4_‐LiNH_2_, Li_3_AlH_6_, Li_2_NH, Li_2_(BH_4_)(NH_2_)	10^−7^–10^−4^	0.3–0.7	Lower grain boundary resistance, Good mechanical strength, Stable with Li, Poor thermal, chemical, and oxidation stability, High reduction stability and ion selectivity
Na^+^	Na_2_(BH_4_)(NH_2_)	10^−4^–10^−3^	0.5–0.8	Expensive for large scale, Average mechanical properties
Mg^2+^	Mg(BH_4_)(NH_2_), Mg(BH_4_)_2_(en), Mg(BH_4_)_2_(NH_3_), Mg(BH_4_)(BH_3_NH_3_)_2_, Mg(BH_4_)_2_.xNH_3_, Mg(BH_4_)_2_.xNH_3_‐MgO	10^−7^–10^−5^, (10^−3^ at 70–100 °C)	1–1.5	Good mechanical strength, Lower grain boundary resistance, Poor compatibility with cathodes Poor cation transfer
Ca^2+^	CaB_12_H_12_, CaBH_4_	10^−9^–10^−5^ (800–1200 °C)	0.65–1.5	Low migration barriers, Poor ion conductivity, High interface resistance, Poor chemical compatibility
**NASICONs**				
Li^+^	LiZr_2_(PO_4_)_3_, LiTi_2_(PO_4_)_3_, Li_1+x_M_x_Ti_2‐x_(PO_4_)_3_ at M = Cr, Al, Fe, Ge, Sc, In, Y, Lu, La; Li_1+x_Al_x_Ge_2−x_(PO_4_)_3_	10^−11^–10^−8^, 10^−3^ (300–500 °C)	0.4–0.6	Poor ionic conductivity, Exceptional electrochemical stability window
Na^+^	Na_3_Zr_2_PSiO_12_, Na_3_Zr_2_Si_2_PO_12_,	10^−10^–10^−7^, 10^−3^ (300–500 °C)	0.3–0.6	Low ion conductivity, Large grain boundary resistance
Mg^2+^	MgZr_4_(PO_4_)_6_	10^−12^–10^−11^, 10^−4^ (800–1000 °C)	0.8–1.0	Poor ion conductivity and chemical compatibility
Zn^2+^	ZnZr_4_(PO_4_)_6_	10^−12^–10^−11^, 10^−5^–10^−4^ (800–1000 °C)	0.9–1.3	Lowest ion conductivity, Poor chemical and electrochemical stability, Operates for high temperatures
K^+^	KZr_2_(PO_4_)_3_, KTi_2_(PO_4_)_3_,	10^−12^–10^−9^	–	Poor ion conductivity and electrochemical (oxidation, reduction) stability, Expensive
Al^3+^	(Al_0.2_Zr_0.8_)_20/19_Nb(PO_4_)_3_	10^−6^–10^−4^ (300–600 °C)	>3.1	Poor ion conductivity and electrochemical stability
Ca^2+^	(Ca_x_Hf_1−x_)_4/(4−2x)_Nb(PO_4_)_3_, (Ca_0.05_Hf_0.95_)_4/3.9_Nb(PO_4_)_3_, Ca_0.5_Zr_2_(PO_4_)_3_, CaZr_4_(PO_4_)_6_, Ca_10.5–x_Pb_x_(VO_4_)_7_ for x = 1.9, 3.5, 4.9, Ca_7_MgPbBi(VO_4_)_7_, Ca_7.5_ZnPb_0.5_Bi(VO_4_)_7_, Ca_7.5_CdPb_0.5_Bi(VO_4_)_7_, Ca_8_PbBi(VO_4_)_7_, Ca_3_(VO_4_)_2_, Ca_7.5_Pb_3_(VO_4_)_7_, Ca_6.5_Pb_4_(VO_4_)_7_, Ca_6.5_Pb_4.5_(VO_4_)_7_, ACa_9_(VO_4_)_7_ (A = Gd, Ho, Lu, Er, Eu, Pr, Sm, Bi, La, Nd, Tb, Yb, Y, and Sc),	10^−8^–10^−3^ (300–900 °C)	1.3–1.5	Extremely poor ion‐conductivity for RT, Poor chemical stability and EWs, operates for high temperature only
**Borate or Phosphates**				
Li^+^	Li_2_B_4_O_7_, Li_3_PO_4_, Li_2_O‐B_2_O_3_‐P_2_S_5_, Li_3‐x_Na_x_PO_4_	10^−7^–10^−6^	–	Scalable fabrication process, Good durability, Poor conductivity
**Thin films**				
Li^+^	LiPON, Li_3.4_V_0.6_Si_0.4_O_4_, LiSiPON, LiSON, LiNbO_3_, Li_3_PO_4_/P_2_S_5_, Li_2_S‐SiS_2_‐P_2_S_5_, Li_3.25_Ge_0.25_P_0.75_S_4_, PEO/succinonitrile/LiTFSI, Li_1+x_Al_x_Ti_2−x_(PO_4_)_3_, Li_3_OCl, Li_2_O‐B_2_O_3_‐Li_2_SO_4_, Li_3_PO_4_‐Li_4_SiO_4_, Li_7_La_3_Zr_2_O_12_,	10^−9^–10^−4^	–	Stable with Li and cathode materials, Expensive for large‐scale, Excellent reduction, oxidation, and thermal stability, ion selectivity, Extremely poor mechanical properties and device integration High processing cost
Na^+^	NaPON (Na_4_PO_3_N)	10^−10^–10^−6^ (RT‐80 °C)	0.5–1.05	Poor RT ion‐conductivity, High interface resistance
Mg^2+^	MgPON, MgPO	10^−10^–10^−6^ (400–500 °C)	–	Poor ion conductivity at RT, Limited thermal and chemical stability, High cost

### 5.1 Inorganic SEs

Solid inorganic electrolytes (SIEs) consist of different types according to their crystal structures, such as garnet, sulfide (glasses, LGPS, argyrodite, thio‐LISICON), perovskites, NASICONs, halides, oxides, hydrides, borate or phosphates. Perovskites SEs (Li_3x_La_2/3‐x_TiO_3_) display exceptional Li‐ion conductivity (>10^−3^ S cm^−1^); however, severe reduction of Ti^4+^ with Li‐metal clarifies the incompatibility for Li‐batteries. NASICONs SEs have been demonstrated as the first SEs in the 1960s^[^ ^112^ ^]^ with chemical formula AM_2_(PO_4_)_3_, where A is the Li, Na, K, Mg, Zn, and M the Ge, Zr, Ti, or Si. Sn and Hf substitution improved a lower conductivity of LiZr_2_(PO_4_)_3_.^[^ ^113^ ^]^ Further, Li_1+x_M_x_Ti_2‐x_(PO_4_)_3_ with M = Fe, Sc, Ga, Cr, Al, Lu, In, La, or Y has been demonstrated in which Al is most efficient due to a wide electrochemical stability window.^[^ ^114^, ^115^ ^]^ Lower covalency of Na‐O than Li‐O, exchanging Na^+^ by Li^+^ in Na_1+x_Zr_2_Si_x_P_3−x_O_12_ NASICON degrades the conductivity. The systems were extended for Na, Mg, Zn, and K (in terms of Na_3_Zr_2_Si_2_PO_12_, MgZr_4_(PO_4_)_6_, ZnZr_4_(PO_4_)_6_) with ion conductivities in the range of 10^−7^−10^−12^ S cm^−1^ at room temperature (RT); however, it significantly enhances to 10^−3^ S cm^−1^ for 300–500 (Li, Na) and 800–1000 °C (Mg, Zn).^[^ ^116^, ^117^, ^118^, ^119^, ^120^ ^]^ Li_3_N, Li_3_PO_4_, and LiI have been reported as Li‐ion conductors.^[^ ^121^, ^122^ ^]^ Yin et al.^[^ ^123^ ^]^ reported Li_3_MCl_6_ (M = Y, Sc, In, Ho) based electrolytes, in which Li_0.388_Ta_0.238_La_0.475_Cl_3_ SE possesses 3.02 mS cm^−1^ Li‐ion conductivity at 30 °C with the generation of gradient interfacial passivation layer to stabilize Li‐metal.

Sulfide SEs based on the Li_2_S‐GeS_2_, Li_2_S‐SiS_2_, and Li_2_S‐P_2_S_5_‐Lil further displayed 10^−4^ S cm^−1^ conductivity. Kanno and Murayama^[^ ^124^ ^]^ reported the first crystalline sulfide SEs (Li_3.25_Ge_0.25_O_0.7_S_4_) of 2.2 mS cm^−1^ ionic conductivity. Partial crystalline Li_7_P_3_S_11_ with Li conductivity of 3.2 mS cm^−1^ was reported for glassy Li_2_S‐P_2_S_5_.^[^ ^12^ ^]^ Li‐argyrodite Li_6_PS_5_X (X = Cl, Br, I) was reported by Deiseroth with the highest conductivity of 4.96 mS cm^−1^ using solid‐state reactions.^[^ ^125^ ^]^ The Li_10_GeP_2_S_12_ superion SEs with 12 mS cm^−1^ at RT comparable to conventional aprotic electrolytes for LIBs were reported by Kamaya.^[^ ^10^ ^]^ Tatsumisago et al.^[^ ^126^ ^]^ (Li_7_P_3_S_11_ with 17 mS cm^−1^), Kato et al.^[^ ^11^ ^]^ (Li_9.54_Si_1.74_P_1.44_S_11.7_Cl_0.3_ with 25 mS cm^−1^) and, Iwasaki et al.^[^ ^127^ ^]^ (single crystal Li_10_GeP_2_S_12_ along with [001] orientation with 27 mS cm^−1^) reported the superion conductors. Over time, chlorine‐rich argyrodite (Li_5.5_PS_4.5_Cl_1.5_) exhibited 12 mS cm^−1^ ion conductivity.^[^ ^128^ ^]^ Several promising Li‐argyrodites, such as Li_6‐x_PS_5‐x_ClBr_x_ (0 ≤ x ≤ 0.8) or Li_6+x_M_x_Sb_1−x_S_5_I (M = Si, Ge, Sn) (24 mS cm^−1^), Li_10_Ge(Sb_0.075_P_0.925_)_2_S_12_ (17.3 mS cm^−1^), Li_10.35_Ge_1.35_P_1.65_S_12_ (14.2 mS cm^−1^), and Li_6.5_Sb_0.5_Ge_0.5_S_5_I (16.1 mS cm^−1^) reported superion conductivity surpassing to liquid counterparts.^[^ ^129^, ^130^, ^131^, ^132^, ^133^, ^134^, ^135^, ^136^, ^137^ ^]^ Li^+^ smaller radius allows the fast transfer kinetics, whereas the lower Lewis acidity of Na^+^ enables cation desolvation. Under similar structures, Na SE can have a higher ion conductivity relative to Li SE. SE of Na_3_P_1–x_As_x_S_4_ (0 ≤ x ≤ 1) exhibits 1.46 mS cm^−1^ of Na‐ion conductivity. For comparison, Na, Mg, and Zn‐based sulfide SEs (cubic Na_3_PS_4_, Na_7_P_3_S_11_, Na_2.88_Sb_0.88_W_0.12_S_4_, Na_5−2x_Al_1−x_Ta_x_S_4_, Na_5−2x_In_1−x_Sb_x_S_4_, tetragonal Na_4−x_Sn_1−x_Sb_x_S_4_; MgS‐P_2_S_5_MgI_2_, MgSc_2_Se_4_, MgSc_1.9_Ti_0.075_Se_4_; ZnPS_3_) have been reported with a maximum conductivity of 10^−2^−10^−3^ S cm^−1^.^[^ ^138^, ^139^, ^140^, ^141^, ^142^, ^143^, ^144^, ^145^, ^146^ ^]^ Specifically, mesoporous Zn_y_S_1‐x_F_x_ displays Zn^2+^ conductivity of 0.66 mS cm^−1^.^[^ ^147^ ^]^ Mg^2+^ phosphate SE with NASICON (MgZr_4_(PO_4_)_6_) exhibits 6.1 mS cm^−1^ ion conductivity for 800 °C, which is much poorer than Na^+^, Li^+^, and other SEs. The stronger electrostatic interactions of Mg^2+^ and principal counterions due to the high charge density of Mg^2+^ (205 C mm^−3^) relative to Li^+^ (87 C mm^−3^) illustrate lower mobility of Mg^2+^ at low temperatures.^[^ ^118^, ^148^ ^]^ Zn^2+^ diffusion is also slower, like Mg^2+^ in NASICON structures with 0.0013 mS cm^−1^ at 500 °C. Zn^2+^ ions are octahedrally coordinated by [P_2_S_6_]^4−^ polyanions in the distorted honeycomb of ZnPS_3_. P‐P bonds follow the stretching to improve structural distortion.^[^ ^146^ ^]^

Mg(BH_4_)(NH_2_) based Mg^2+^ SEs show the structural tunnels designed by zigzag chains for Mg ions (distance of Mg‐Mg ≈3.59 Å) and anion frameworks. The distance of Mg‐Mg ions is smaller than those of Mg(BH_4_)_2_; thus, it facilitates the Mg hopping with high ion conductivity of 10^−3^ mS cm^−1^ for 150 °C, EW of >3 V, and reversible Mg plating/stripping.^[^ ^149^ ^]^ However, this Mg^2+^ conductivity is extremely poorer than those of Li^+^ in Li_2_(BH_4_)(NH_2_) (≈0.2 mS cm^−1^ at RT).^[^ ^143^ ^]^ The high Mg^2+^ mobility for spinel selenides (MgSr_2_Se_4_) provides large conductivity of ≈0.01–0.1 mS cm^−1^ at RT.^[^ ^150^, ^151^, ^152^, ^153^ ^]^ Nanoconfined LiBH_4_/Al_2_O_3_ (orthorhombic) or LiBH_4_‐LiI/Al_2_O_3_ (hexagonal) reveals rapid local interfacial Li‐ion kinetics with small *E_a_* of 0.1 eV, which facilitates faster long‐range 2D ion transport. Li_6_PS_6_(BH)_4_ exhibits 1.8 mS cm^−1^ ion conductivity with *E_a_* of 0.015 eV.^[^ ^150^, ^154^, ^155^ ^]^ Mostly reported halide SEs contain Li_3_MCl_6_ (M = Sc, Y, In, Yb, Er) with maximum ion conductivity of ≈1 mS cm^−1^. Na^+^ halide SEs (Na_2_ZrCl_6_, Na_3–x_Er_1–x_Zr_x_Cl_6_, and Na_3–x_Y_1–x_Zr_x_Cl_6_) have limited research due to expensive central metals and poor conductivity.^[^ ^156^, ^157^, ^158^, ^159^ ^]^

Garnet SEs contain the structure of A_3_B_2_Si_3_O_12_ with A and B cations having eight‐ and sixfold coordinations. Since 1969,^[^ ^160^ ^]^ numerous garnets, including (Li_3_M_2_Ln_3_O_12_ (M = W or Te), Li_5.5_La_3_M_1.75_B_0.25_O_12_ (M = Nb or Ta; B = In or Zr), Li_6_ALa_2_M_2_O_12_ (A = Ca, Sr or Ba; M = Nb or Ta), Li_5_La_3_M_2_O_12_ (M = Nb or Ta), and the cubic Li_7_La_3_Zr_2_O_12_ and Li_7.06_M_3_Y_0.06_Zr_1.94_O_12_ (M = La, Nb or Ta) have been reported with highest ion conductivity of 1.02 mS cm^−1^ for the Li_6.5_La_3_Zr_1.75_Te_0.25_O_12_ at RT.^[^ ^66^, ^67^, ^68^, ^161^, ^162^, ^163^, ^164^, ^165^, ^166^ ^]^ β‐Alumina SE (BASE) is the Na^+^ ion conductor for Na‐S high‐temperature batteries. Similarly, K‐incorporated BASE shows K^+^ ion conductivity. K‐BASE displays 10 and 56 mS cm^−1^ conductivity for 150 and 300 °C, respectively. Besides, Eremin et al.^[^ ^167^ ^]^ reported K^+^ ion conductors (K_5_As_3_O_10_, K_4_V_2_O_7_, K_2_Zn_3_O_4_, K_2_Sb_4_O_11_, K_3_NbAs_2_O_9_, and K_3_NbP_6_) based on their diffusion pathways and *E_a_*. K_4_V_2_O_7_ and K_2_Al_2_Sb_2_O_7_ show promising 2D and 3D conducting pathways. Yuan et al.^[^ ^168^ ^]^ reported the open framework K‐ferrite (K_2_Fe_4_O_7_) as additional K^+^ SE with *c*‐ and *b*‐axes. It comprises both FeO_4_ tetrahedra and FeO_6_ octahedra units for edges and corners, offering proper K^+^ diffusion pathways. Polycrystalline K_2_Fe_4_O_7_ displays 50 mS cm^−1^ at RT (*E_a_* of 0.16 eV), while a single crystal along the *a*‐axis reveals 350 (RT) and 500 mS cm^−1^ (500 °C) with *E_a_* of 0.08 eV. MgHf(WO_4_)_3_ exhibits 0.25 mS cm^−1^ conductivity at 600 °C owing to 1D alignments of Mg^2+^ and Hf^4+^ ions interchanging for quasi‐layered WO_4_^2−^ units at Sc^3+^ sites in Sc_2_(WO_4_)_3_.^[^ ^169^, ^170^ ^]^ Mg_1+x_Zr_4_P_6_O_24+x_ + xZr_2_O(PO_4_)_2_ and Mg_0.5_Zr_2_(PO_4_)_3_ display 10^−7^–10^−9^ S cm^−1^ at 500 °C.^[^ ^169^, ^170^, ^171^ ^]^ Aubrey et al.^[^ ^172^ ^]^ reports Mg_2_(2,5‐dioxidobenzene‐1,4‐dicarboxylate) and Mg_2_(4,4′‐dioxidobiphenyl‐3,3′‐dicarboxylate), and MOFs‐based SEs with *σ_Mg_* ^2+^ ≈0.25 mS cm^−1^ at RT.

### 5.2 Organic SEs

Polymer electrolytes are classified into three categories: 1) dry solid polymer electrolytes (SPE), gel polymer electrolytes (GPE), and composite polymer electrolytes (CPE) for different types of battery chemistries. SPEs involve the metal salts (Li, Na, Al, Zn, K, Mg, Ca) solid solvents with polymer hosts deprived of any liquids. However, their ion conductivities are extremely poor at RT.^[^ ^173^ ^]^ GPE consists of metal salts and polymeric networks liquefied by water, which feature liquid‐like ion conductivity and solid‐like compatibility. However, it suffers from large interfacial resistance, poor mechanical properties with high swelling behavior, and rapid degradation with ion conductivity.^[^ ^174^ ^]^ CPEs formed by the combination of polymer hosts with inorganic ceramic fillers to improve conductivity by reducing *T_g_*. Generally, PEO, PAM, PMMA, PVC, PVDF, or PAN are utilized as polymers along with active (e.g., Li_2_N, LiAlO_2_) and inactive (e.g., MgO, SiO_2_, Al_2_O_3_, TiO_2_) fillers.^[^ ^175^, ^176^ ^]^ CPEs determine the compatibility for Li‐metal and the high‐voltage cathodes, which is a critical requirement for high‐energy SSBs. Theoretical results show thermodynamics for electrode/electrolyte interface and intrinsic EWs of CPEs with the role of inorganic fillers for leading EWs.^[^ ^39^, ^177^, ^178^ ^]^

The dual functioning PSA and poly(dimethyl siloxane)‐g‐[poly(poly(ethylene glycol) methyl ether methacrylate)‐r‐sodium poly(p‐styrene sulfonate) (PPS) as SPE and artificial SEI with 0.056 and 0.45 mS cm^−1^ conductivities following VFT mechanism have been reported.^[^ ^179^, ^180^ ^]^ In hybrid electrolytes (SIE‐SPE or SIE‐liquid) for Li and Na ions, the Na^+^ transport occurs via internal interfaces with smaller *E_a_* due to the lower Lewis acidity of Na^+^ and weak interactions for oxygen atoms in polymers or liquids. Theoretical calculations verify the desolvation energy trend as Mg^2+^ > Li^+^ > Na^+^, enabling cations suitability for new SEs.^[^ ^181^, ^182^ ^]^ Since the 1980s, Mg‐based SPEs have been reported; however, GPE composed of PVDF or PEO with Mg(AlCl_2_–EtBu)_2_ and tetraglyme plasticizer displayed reversibility of Mg under plating/stripping.^[^ ^183^ ^]^ Further, Mg(BH_4_)_2_‐PEO‐MgO CPE exhibits the Mg plate/stripping for >98% CEs at 100 °C with dissociation of Mg(BH_4_)_2_.^[^ ^184^ ^]^ PEO with Mg(TFSI)_2_, Mg(NO_3_)_2_, and Mg(Tf)_2_ displays conductivities of 0.0001, 0.013, 0.000032 mS cm^−1^ at RT, respectively.^[^ ^102^, ^185^, ^186^, ^187^ ^]^ PVDF‐HFP with 40 wt% Mg(Tf)_2_ shows maximum conductivity of 10^−3^ S cm^−1^ at RT.^[^ ^187^ ^]^

Zn^2+^ SPEs involve (PEO‐ZnCl_2_, Zn(TFSI)_2_, Zn(OTf)_2_) with a thickness of 30–60 µm.^[^ ^188^, ^189^ ^]^ For alkaline Zn‐batteries, the transfer of hydroxides is a main source compared to Zn^2+^ ions, so such GPEs use basic KOH/NaOH for oxygen solubility and conductivity. Cellulose with quaternary ammonium salts and chitosan biocellulosics membrane inhibits the crossover of cations with 21.2 and 86.7 mS cm^−1^ hydroxide conductivity.^[^ ^190^, ^191^ ^]^ Further, the ethylene glycol‐based waterborne anionic polyurethane acrylates/PAM (EG‐waPUA/PAM) GPE enables freeze‐resistance down to −20 °C.^[^ ^192^ ^]^ Al^3+^‐based SEs offer a high capacity of 8040 mAh cm^−3^ due to the removal of corrosion and moisture effect by liquids.^[^ ^32^ ^]^ For example, SPE of 1‐ethyl‐3‐methylimidazolium chloride (EMIC)–AlCl_3_ exhibits 1.46 mS cm^−1^ conductivity, Al plate/stripping, and fast charge capability (<10s) for graphite cathodes.^[^ ^32^, ^77^, ^193^, ^194^ ^]^ Potassium bis(fluorosulfonyl)amide (KFSA) with 1,2‐dimethoxyethane and 1,3,2‐dioxathiolane 2,2‐dioxide suppresses interfacial resistance and polarization with 0.02 mS cm^−1^ ion conductivity. Degrees of polarization show the tradeoff as Li^+^ > Na^+^ > K^+^.^[^ ^195^, ^196^ ^]^ Various complexes of PEO with Li, Na, K, Zn, Mg, and Al (i.e., NaSCN, NaTFSi, NaPF_6_, NaYF_6_, KYF_6_, LiSO_3_SF_3_, LiTFSI, LiFSi, ZnTFSI_2_, MgTFSI, AlCl_3_) have been explored for SPEs.^[^ ^32^, ^102^, ^179^, ^180^, ^181^, ^182^, ^183^, ^184^, ^185^, ^186^, ^187^, ^188^, ^189^, ^190^, ^191^, ^192^, ^193^, ^194^, ^195^, ^196^ ^]^

Zhou et al.^[^ ^197^ ^]^ presents in situ polymerized poly (ethylene glycol) diacrylate‐based electrolytes with σ_Na_^+^ ≈1.4 mS cm^−1^ at 25 °C. The refined solvation structure of Na^+^ restrains the random diffusion of Na^+^‐ions due to lower desolvation energy barriers with suppression of dendritic growth over >2000 h Na//Na cells. Yuan et al.^[^ ^198^ ^]^ reports polyvinylidene fluoride hexafluoropropylene (PVDF‐HFP)‐based solid electrolyte that can operate over −20 to 50 °C with high capacities of 165.6/163.4 mAh g^−1^ at 50/25 °C. Hou et al.^[^ ^199^ ^]^ reports 1,3,5‐trioxane (TO) and DME‐based electrolytes with the construction of mechanically stable SEI enriched with organic components in Li‐S batteries. High polymerization of TO displays preferential decomposition with alleviating cracks, regeneration of SEI, and reduction in consumption rate for Li‐polysulfides and active Li. Wang et al.^[^ ^200^ ^]^ fabricates the SPEs with 3‐(1‐vinyl‐3‐imidazolio) propanesulfonate (VIPS) zwitterionic monomer with *σ_Zn_* ^2+^ ≈2.6 mS cm^−1^ under 20 wt% lean‐water content, wider voltage stability window, and Zn plate/strip over 900 h with dense and dendrite‐free surfaces. Functionalized biocellulosics (FBN) and chitosan‐biocellulosics (CBCs) membrane electrolytes with *σ_Zn_* ^2+^ ≈64 and 86.7 mS cm^−1^ for highly reversible Zn‐air batteries are also reported.^[^ ^201^, ^202^, ^203^, ^204^, ^205^, ^206^, ^207^, ^208^ ^]^ Pristine and patterned Zn anodes with FBN and CBCs displayed superior compatibility under plating/stripping over 5000–6000 cycles with capacities of 1–10 mAh cm^−2^ per cycle. Further, both electrolytes exhibit chemical and thermodynamic stability for supramolecular polymer intertwined free‐standing and powder‐based oxygen cathodes with high power and energy efficiencies.

### 5.3 Thin‐Film (TF) SEs

Thin film SEs are prepared using various techniques such as chemical vapor or atomic layer or pulsed laser deposition, radio frequency sputtering, thermal/electron‐beam evaporation, printing, sol–gel/aerosol, and electrodeposition. Required properties for implementing thin film SEs are RT ion conductivity of 10^−9^–10^−6^ S cm^−1^, EW of >5 V versus Li/Li^+^, high electrochemical/thermal stability for anode/cathodes, and facile large‐scale manufacturing. Bates et al.^[^ ^209^, ^210^, ^211^ ^]^ reported amorphous LiPON TFSEs with general formulation as Li_x_PO_y_N_z_. Li_3_PO_4_ and N‐incorporated Li_3_PO_4_ exhibiting 0.00007 and 0.0033 mS cm^−1^ ion‐conductivity with EW of 5.5 V versus Li/Li^+^. Further, binary/ternary oxides like Li_2_O‐Nb_2_O_5_, Li_2_O‐SiO_2_‐P_2_O_5_, Li_2_O‐B_2_O_3_‐P_2_O_5_, and Li_2_O‐SiO_2_‐V_2_O_5_ showed 10^−7^ to 10^−5^ S cm^−1^ conductivity range. Amorphous (LLZO, LLTO, sulfides), PEO‐composites, and crystalline (Li_3_OCl, NASICON, LISICON, LLZO, LLTO) based TFSEs reported the maximum ion conductivity of 0.14 mS cm^−1^.^[^ ^212^, ^213^, ^214^, ^215^ ^]^ Lupo et al.^[^ ^216^ ^]^ reported that Li phosphorous sulfuric oxynitride (LiPSON) SEs display *σ_Li_* ^+^ ≈9.75 µS cm^−1^ slightly larger than LiPON (3.3 µS cm^−1^). *E_a_* for LiPSON, LiSiPON, and LiPON are 0.49, 0.41–0.47, and 0.49–0.68 eV, respectively.^[^ ^217^, ^218^, ^219^, ^220^, ^221^, ^222^ ^]^ Song et al.^[^ ^223^ ^]^ reports Mn‐doped LiPON thin film SEs with *σ_Li_* ^+^ ≈5 µS cm^−1^ and reduces the work function of LiPON that benefits electrochemical interface stability. Mn‐doping restricts the blocking effects of SCL for Li^+^ due to weakening Li^+^‐PO_3_N^4−^ tetrahedral bindings. The ionized N‐ions bounds with phosphate by forming double‐ligand N (N_d_, P‐N═P) and tri‐ligand N (N_t_, $P-N{<}_{P}^{P}$) during thin film formation, and the multivalent nature of Mn controls an assortment of LiPON binding types.^[^ ^224^ ^]^ Mn‐doping serves as a junction for crosslinks for phosphate groups (i.e., Mn^4+^ to N_d_ and Mn^3+^ to N_t_) conducive for isotropic Li^+^‐transport. López‐Grande et al.^[^ ^225^ ^]^ reports Li_2_PO_2_N oxynitride with nitridation of crystalline Li_4_P_2_O_7_ by thermal ammonolysis. Theoretic calculations of Li_2_O–P_2_O_5_–P_3_N_5_ equilibria predict no formation of Li_2_PO_2_N lower than 7.3 wt% N. It forms by decomposing γ‐Li_3_PO_4_ into Li_2_PO_2_N and Li_2_O with increasing N contents. Low N displays two major resonances in NMR for Li_4_P_2_O_7_ (−3.9 and −6.1 ppm) and Li_3_PO_4_ (9.45 ppm) with minor four Lorentzian profiles (4.74, 10.79, 11.94, and 14.42 ppm), which illustrates the existence of P‐atoms for one or more crystals. High N of 7.98 wt% displays similar resonances with 4.34 wt.% N, excluding Li_4_P_2_O_7_ phase.

Similar to LiPON, sodium phosphorus oxynitride (NaPON) is also reported with a conformal stoichiometry of Na_4_PO_3_N analogous to sodium polyphosphazene structures and *σ_Na_* ^+^ ≈0.1 and 2.5 µS cm^−1^ for 25 and 80 °C and *E_a_* ≈0.53 eV, two times higher magnitude than air‐exposed films.^[^ ^226^ ^]^ XPS results clarify the presence of Na^+^‐O^−^,═P−, O^−^‐Na═, doubly‐coordinated N (P‐N═P), and triply coordinated N (P‐N<P_2_). Fontecha et al.^[^ ^227^ ^]^ also reported NaPON using PEALD for 150–350 °C and obtained *σ_Na_* ^+^ ≈8.2 nS cm^−1^ at 80 °C. N content and coordination states of N (N_d_ or N_t_) in NaPON or LiPON are crucial for high‐ion mobility. Lacivita et al.^[^ ^214^ ^]^ confirms the higher N_t_ coordination states suffer poor ion conductivity, and high N_d_ offers large ion conductivity for LiPON and NaPON. Bulk Li_3.6_PO_3.4_N_0.6_ (b‐LiPON) crystalline polymorph is also prepared by ball‐milling with *σ_Li_* ^+^ ≈5 µS cm^−1^ for 70 °C and 5 V versus Li/Li^+^ window.^[^ ^228^ ^]^ b‐LiPON consists of distorted hcp arrays of O and N anions. N‐anions partially occupy two crystallographic O‐sites as O1/N1 and O2/N2, whereas P‐cations occupy PO_3.57(13)_N_0.43(13)_ coordinated tetrahedra isolated from others and consecutively indicate opposite directions. Li‐cations have six distinct places similar to Li_4_SiO_4_ in terms of Li6 for octahedral, Li5 for fivefold, and Li4 for tetrahedral coordinations.

Magnesium phosphorus oxynitride (MgPON) SEs are also reported by using ALD with double nitrogen plasma processes at a low deposition temperature of 125 °C.^[^ ^229^ ^]^ MgPON displays amorphous nature without grain boundaries and *σ_Mg_* ^2+^ ≈0.58 nS cm^−1^ at 300 °C, 1.1 times larger than magnesium phosphate (MgPO). For 400 and 500 °C, it shows 6.2 and 7.5 times larger *σ_Mg_* ^2+^. Tsuruoka et al.^[^ ^230^ ^]^ reports MgPO thin film SEs by developing alternative Mg‐O and P‐O sub‐cycles at 125–300 °C temperatures. The lower temperature synthesis persuades multi‐bonding states for the phosphate matrix with a mixture of metaphosphates and pyrophosphates as well as a disordered matrix for Mg^2+^‐ions hopping conduction. The phosphate glass (P_2_O_5_) involves PO_4_ tetrahedra with P‐O‐P linkages. PO_4_ tetrahedra link to neighboring tetrahedra by three vertices creating P–O^−^ bonds and residual vertices terminate with double‐bonded P═O.^[^ ^231^, ^232^ ^]^ Mg‐O‐P displays thermally activated hopping among these non‐bridging oxygen with *σ_Mg_* ^2+^ ≈10^−7^ to 10^−11^ S cm^−1^ from 400 to 200 °C. Su et al.^[^ ^170^ ^]^ reports the ALD MgPO displays *σ_Mg_* ^2+^ ≈0.16 µS cm^−1^ and *E_a_* ≈1.37 eV for 500 °C. RF‐sputtered Mg_3_(PO_4_)_2_ film SEs also reported; however, it shows poorer conductivity than MgPO‐ALD. Electrochemical and interfacial instability for low/high potentials, decompositions, and volume changes during short circuits or cycling due to metal dendrites are the major failure modes for SEs.

## 6 Interface Challenges

The fundamental understanding of the interfaces between SEs, anodes, and cathodes (notably ionic and electronic transport kinetics) is essential for high‐performance SSBs. The perfect interface requires minimal interfacial impedance, atomic scale conformal contacts, and electrochemical/mechanical stability. Practical cells exhibit several interfacial issues such as physical/chemical contact, lattice mismatch, ion conductivity, formation of interdiffusion layer and space‐charge layer, Li‐immobilization, microstructure, and dendrite growth, chemical reactivity of SEI, and thermal instabilities.

### 6.1 Physical and Chemical Contacts

**Figure 4:**
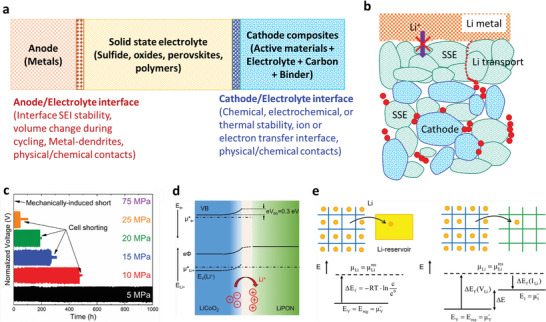
a) Compatibility issues for SSBs interfaces. b) Schematics for interfacial contact issues for Li/SSEs and cathode/SSEs. c) Li symmetric cells performances versus different stack pressures. Reproduced with permission.^[^ ^238^ ^]^ Copyright 2020, Wiley VCH. d) Structural illustration of as prepared LiCoO_2_–LiPON interface (top side) and after annealing (bottom side). e) Defect formation energies in the energy level diagram. At equilibrium, the defect formation energy (single) is recognized for variance in the energy levels (*E*_V_, standard chemical potential μ°_V_) and chemical potential μ, as denoted using the defect concentrations *c* (left). For defect pairs, the change of defect formation energies for a defect signifies the dissimilarity among the energy levels (if two materials are involved in forming the defect pair, right). Reproduced with permission.^[^ ^248^ ^]^ Copyright 2017, American Chemical Society.

Ionic and electrical contacts of SEs and electrodes are highly preferred for achieving high‐performance SSBs. The point‐to‐point contacts are frequently observed for SE/electrode interfaces due to the particle‐particle connections or pore existence even under hot pressing, which provides the concerted local electric field and high interface resistance. Surface energy mismatch induces poor wetting for SEs with metals (Li, Na, K, Zn, Al, Ca) and inadequate contacts between them, illustrating poor rate capability, high charge‐transfer polarization, and lower energy densities (**Figure** **4**a,b). Volume discrepancy of metals during cycling initiates dendritic growth. Dendrites formation strongly depends on the operating current density of cells. For high current densities, the resulting high electric field causes uneven metal plate/strip and grain boundaries of SEs, which induces the voltage penetration of SEs and vital short‐circuiting for SSBs. Thus, the rational design of the interface of SEs and metals is highly challenging. Utilization of polymer buffers, artificial SEI, and preactivation for lower current densities are several approaches reported to develop Li (or other metals)/SEs.^[^ ^233^, ^234^, ^235^ ^]^ Similarly, physical contacts among the SEs and cathodes are also crucial to recognize lower impedance SSBs. It depends on several factors, including surface morphology of SEs and active cathodes, conductive additives, and stress/volume variations for cathodes during cycle operations. Insertion of buffer layers (i.e., polymers, sulfides, oxides, or hybrids)^[^ ^236^, ^237^ ^]^ can enhance physical/chemical contacts with a considerable decrease in interfacial resistance, rate capacity, cycle life, and energy‐power performances.

Another approach is applying high pressure during cell fabrication and operations to enhance the mechanical contacts. However, optimal stack pressure is recommended as per metal‐chemistries. For Li‐metal, Doux et al.^[^ ^238^ ^]^ reported the optimal stack pressure of ≤25 MPa for Li/Li_6_PS_5_Cl/Li symmetric cells that enhance Li/SEs contact with decreasing interfacial resistance. Figure 4c displays the influence of stack pressure on the mechanical integrity of SEs versus cycling, in which no obvious shorts are observed up to 1000 h for 5 MPa pressure. This reveals that 5 MPa stack pressure enables the reasonable contact of Li and SE. Cells show failure after 474, 272, 190, and 48 h for 10, 15, 20, and 25 MPa stack pressures during cycling, which ascribes the creeping of Li in the pores of SEs wherein Li dendrites form with eventual short‐circuit. Above 75 MPa, cells are mechanically shorted before applying current. Generally, composite cathodes exhibit enhanced kinetics compared to pristine active cathodes with fast ion transport; however, they adversely decrease the electrode mass loading, degrading the operative storage capacity. Zero‐strain cathodes diminish the internal stress of SSBs, Koerver et al.^[^ ^239^ ^]^ reported that the blending of LiCoO_2_ (LCO) and LiNi_0.8_Co_0.1_Mn_0.1_O_2_ (NCM811) in 55:45 wt% displays nearly zero‐strain. LCO reveals positive stress during operations, whereas NCM811 has negative stress responses.

To obtain stable performance for SSBs, the chemical contacts related to the electrochemical and chemical stabilities of an interfacial reaction region are also critically important. For example, the sulfides, thiophosphate, titanium, and germanium are unstable in contact with thermodynamically uneven Li metal, and a solid passivation layer gradually forms at Li/SSE interface. There are three types of interphases, as explained in Section 2. The mixed conducting interface (type 3) forms Li dendrites severely. Further, the high electronic conductivity of SEs and overpotential during Li plating also demonstrate the dendrite's growth in bulk SE. Thus, incorporating reactive/nonreactive buffer layers with Li shows the electrochemically and thermodynamically stable SEI construction.^[^ ^235^, ^240^, ^241^ ^]^ Besides, SSE/cathode interface involves the flow of electrons through the cathodes and ions across the cathode/SSE interfaces. Cathode encloses active materials, carbon conductors, and polymer binders, providing a concurrent transfer of Li ions and electrons. Intrinsic resistance of one of these materials can result in poor solid interface contacts (Figure 4b). Different chemical potentials of cathodes/SEs form the larger contact resistances for metal ions from the space charge layer. For example, sulfide SEs have a weak attraction towards Li^+^ with low chemical potential relative to the oxide cathode (LiFePO_4_). In sulfide‐based SEs, Li‐ions transfer in the oxide cathodes with redistribution of Li ions while forming a depletion layer along the oxide cathode/sulfide SEs interface that offers the additional energy barrier for Li^+^ ions transport. Loading of thin layers of Li_4_Ti_5_O_12_, LiNbO_3_, LiAlO_2_, and LiTaO_3_ can effectively suppress space charge layers at SSE/cathode interface, as per previous reports. The compositions and structures of buffers significantly control the interface compatibility of SEs/cathode.^[^ ^242^, ^243^, ^244^ ^]^ According to electronic and Li‐ion conductivities, generated interlayers are classified into four groups: 1) interlayer with high ionic and electronic conductivities that unceasingly grow under cycling. 2) Interlayer with high electronic and low ionic conductivities, which causes degradation of SEs and growth of thick interlayer. 3) Interlayer with low electronic and ionic conductivities, which displays substantial interfacial resistance without interphase growth and poor performance of cells. 4) Interlayer with low electronic and high ion conductivities, which has initial growth due to parasitic reactions. The formation has been inhibited after a certain thickness of the interlayer is obtained. These interlayers will enhance the EW of SEs without enlarging interfacial resistance.^[^ ^245^ ^]^ Practical cells display several interlayers over an interface like Li‐immobilization, space charge, or interdiffusion. Conclusively, microstructures, operating conditions, and uniformity of materials realize the interface contacts as per battery chemistries (Na, K, Mg, Zn, Al, Ca, etc.).

### 6.2 Space‐Charge Layers

Interfacing two electrodes with different electrochemical potentials projects the space‐charge layers (SCL), in which simply one charge species can transfer (either electrons or ions) with a building interfacial charged region (noted as space charge layer). The impact of space charge layers on cell performances is highly debatable. Theoretical calculations based on silica or Al_2_O_3_ fillers in SEs display increased ion conductivity due to a space charge layer. In contrast, most reports stated that a space charge layer is not applicable. LiMn_2_O_4_//Li_3.25_Ge_0.25_P_0.75_S_4_ and LiCoO_2_//Li_3_PS_4_ systems vindicate the high interfacial resistance due to space charge layers.^[^ ^65^, ^246^ ^]^ Theoretical results estimated that a possible thickness of the space charge layer is  ≈1 nm; thus, the influence of such resistance will be negligible apart from Li depletion in SEs.^[^ ^247^ ^]^ Fingerle et al.^[^ ^248^ ^]^ reported the in‐depth analysis of interface equilibria and reactions required to transfer ion and electronic species through the interface or near‐interface regions for LCO and LiPON materials. The electrostatic potential gradient of 0.3 V is observed for the pristine interface, and it remained even after annealing, as verified by band bending in Figure 4d, which is ascribed to the equilibration of the electrochemical potential of Li at the interface.^[^ ^249^ ^]^ The Li‐ions losses from LCO state the construction of negatively charged Li‐vacancies (Vˈ_Li_) and Li‐ions incorporation in the LiPON SEs that recognized generation of positively charged Li‐interstitials (Li^·^_I_). Li‐interstitials and Li‐vacancies accumulate the space charge layers at the interface subject to the charge carrier concentrations (i.e., Debye length).^[^ ^250^ ^]^ Such a prolonged space charge layer (a few nm) in LCO rationalizes a low charge carrier concentration (lower number of Li‐vacancies), as denoted by the Fermi level. For LiPON, mobile Li‐ion concentration is 1.5 × 10^20^ cm^−3^, which verifies the compact space charge layer.^[^ ^251^ ^]^ Defect formation energies and energy level structure are displayed in Figure 4e. Defect formation energy of LCO and LiPON is >3 and 0.5 eV depending on the Li‐vacancy/Li‐interstitial pairs.

Wang et al.^[^ ^252^ ^]^ reported in‐situ visualization of space‐charge layers for LCO/Li_6_PS_5_Cl or BaTiO_3_‐LCO/ Li_6_PS_5_Cl interfaces, in which they verified that the built‐in electric field and chemical coupling strategies could minimize the SCL to improve Li‐transport across the interface. Gu et al.^[^ ^253^ ^]^ reported the SCL in Li_0.33_La_0.56_TiO_3_, the grain boundary cores have excess Li hosted at the 3c interstitial sites, whereas the bulk structure accommodates Li with A‐sites. 2D nuclear magnetic resonance results display the intense Li exchange across the Li_x_V_2_O_5_‐LAGP interface at x = 1 without SCL; however, x = 0.2 and 2 inhibit the Li exchange due to the presence of SCL.^[^ ^254^ ^]^ If grain boundary defects have the mobile charge density, SCL width is independent of space charge potential (i.e., Gouy‐Chapman case); if not, SCL width relies upon space charge potential (i.e., Mott‐Schottky case).^[^ ^255^ ^]^ Several approaches have been demonstrated to alleviate space charge layers; however, inserting a buffer layer at the electrodes/SEs is the most suitable approach. Other battery chemistries are not researched for SCL's influence, which might be due to poor storage capacity compared to commercial LIBs.

### 6.3 Interdiffusion

The two adjacent materials mutually diffuse to each other with the formation of interlayers, causing interdiffusion issues, including decomposition or dissolution of SEs, electrodes with high interfacial resistance, severe capacity losses, and rate performances. Elemental interdiffusion during thermal processes and electrochemical cycling is the decisive concern. Thermal methods are utilized to fabricate electrode materials, which typically facilitates cross‐over among the electrodes and SEs with an elemental exchange or interphase formations along with SEs/electrode interfaces. Miara et al.^[^ ^256^ ^]^ reported the chemical compatibility for spinel cathodes (LiCoMnO_4_, Li_2_FeMn_3_O_8_, Li_2_NiMn_3_O_8_) and SEs (Li_6.6_La_3_Zr_1.6_Ta_0.4_O_12_ and Li_1.5_Al_0.5_Ti_1.5_(PO_4_)_3_) during thermal processing, which exhibits spinel cathodes incompatible with Li_6.6_La_3_Zr_1.6_Ta_0.4_O_12_ and Li_1.5_Al_0.5_Ti_1.5_(PO_4_)_3_ over 600 °C due to the formation of ionically insulating interphase with high interfacial resistances. Xu et al.^[^ ^257^ ^]^ reported that the interface of LiMn_2_O_4_–Li_0.33_La_0.57_TiO_3_ displays an interdiffusion region of 300 µm including Mn, La, and Ti for 900 °C. Such interdiffusion is observed for spark plasma and co‐sintering processes that verify 40 times higher interfacial impedances. Zhang et al.^[^ ^258^ ^]^ reported interdiffusion of transition metals for LiNi_0.85_Co_0.1_Mn_0.05_O_2_//Li_6_PS_5_Cl configuration. Cryo‐TEM results show a 10 nm thick amorphous layer containing K, Mn, Co, Ni, and O elements during cycling. Loss in energy from 12 to 8.7 eV (bulk CE) and 10.8 eV for interface region verifies the diffusion of Mn, Co, or Ni in +4 to +3 and +3 to +2 states. Kim et al.^[^ ^259^ ^]^ reported that interparticle diffusion through the graphite electrode interface can enhance the energy density and rate capacity. Besides, anode/SEs also show interdiffusion for LCO//Li_3_PO_4_//Si cells. The initial state did not show interdiffusion elements, whereas the first lithiation of the Si anode displays the transfer of Si in SEs, which deteriorates the capability of active materials.^[^ ^260^ ^]^ The diffusion of P and S from SEs to cathode is observed for LiNi_0.8_Co_0.15_Al_0.05_O_2_//75Li_2_S–22P_2_S_5_–3Li_2_SO_4_ interface illustrating higher interface impedance. The buffer coating of LiInO_2_ and LiInO_2_–LiI over cathodes prevents interdiffusion by reducing interface resistance.^[^ ^261^ ^]^ Similarly, interdiffusion is observed for Na, Mg, K, and Zn ion battery chemistries.^[^ ^262^, ^263^, ^264^, ^265^ ^]^ Various approaches have been reported to prevent interdiffusion; however, artificial buffer layer loading is often promising. **Table** **2** demonstrates the summary of buffer layer materials for different battery chemistries.

**Table 2:** Summary of buffer materials for SSBs under different battery chemistries.

Metal ions	Buffer materials	Thickness range (nm)	Deposition method	Remarks
Li^+^	Al_2_O_3_, carbon/graphite, Li_3_N, Li_3_PO_4_, Li_3_NBO_4_, LiTaO_3_, BaTiO_3_, ZrO_2_, ZnO, Ge, Nb, Sn, Bi, Au, Mg, Al, Li_2_SiO_3_, SFC/CNT, Mg_3_N_2_, Li_x_Zn_y_	2–1000	Sputtering, sol‐gel, ALD, CVD, PLD, spray, Thermal/ Electron‐beam evaporation,	Good chemical stability, Prevent interdiffusion, Severe wetting and dendrites issues, Contact issue, Space charge layer formation
Na^+^	Mg, Ca, carbon, Al_2_O_3_, ZrO_2_, Au, SFC/CNT, Mg clusters, AlF_3_, Na‐K alloy	5–50	ALD, PLD, CVD, sputtering	Contact issue, Chemical and electrochemical stability issues, Wetting and dendrites issue, Interdiffusion issue
Mg^2+^	Carbon, Al_2_O_3_, ZnO, Au, Sn, Phytic acid, CNT,	5–100	Sputtering, sol‐gel, ALD, CVD, PLD, spray, Thermal evaporation,	Chemical and electrochemical stability issues, Wetting and dendrites issue, Interdiffusion issue,
Zn^2+^	ZnO, ZnS, ZnF_2_, MXene, CNT, polymers, graphene, carbon, graphite, NC, Ag, MOF, graphdiyne, Polyamide, PAN, PAM, PPA, PAA, Zn‐other metal alloys, glue films	5–100	ALD, Immersion, sol‐gel, Thermal evaporation	Good chemical and electrochemical stability issues, Prevent dendrites and parasitic reactions, Good surface wetting nature

### 6.4 Lattice Mismatch

Lattice mismatch typically occurs for all interfaces (SEs/anode or SEs/cathode) with deviation in the microstructures and lattice parameters, which initiates the formation of superlattice and strain/stress that illustrates the high interfacial impedance. Generally, there are two types of interfaces with lattice mismatch: 1) identical crystal structures and analogous lattice parameters that enable the fastest metal‐ion transport. 2) Strong deviation in the lattice and crystal structures shows complex metal‐ions transfer. Thus, the minor lattice differences in interface materials exhibit smaller interface impedances with superior ion conductivity than large deviated lattice structures. However, a large deviation in crystal lattices is the present scenario of SSBs. Metal‐ions (Li, Na, Mg, Zn, Al, K, Ca) transport relies on different parameters, including ion conductivity, grain boundaries diffusion characteristics, interface impedance, and lattice structures at interfaces. Poor metal‐ion transport is ascribed to lattice‐mismatched interfaces and meager bulk ion conductivity.

Interface formation energy is the difference between energy sums for two stress‐free pristine phases with the same atomic numbers and totally relaxed interface structures. Interface formation energy (*E_f_*) with supercell A and B constituents is stated as^[^ ^266^ ^]^

(14) $$E_{f}=E_{AB}-N_{A}E_{A}-N_{B}E_{B}$$

Where *E*_*A* *B*_ is the total energy for a totally relaxed interface, including supercell *N*_*A*_ and *N*_*B*_ units for A and B, respectively, *E*_*A*_ and *E*_*B*_ are the energy for pristine A and B bulk structures (without lattice mismatch). Interface formation energy can be divided into interfacial and strain energy relating to the elastic deformation of A and B in the coherent interface as

(15) $$E_{f}=2S\sigma +VE_{\mathrm{Elastic}}$$

Where *S* is the interfacial area, *E*_*elastic*_ the elastic strain energy per unit volume, *V* the volume totally relaxed cell, *σ* the interfacial energy, and the value two is related to the two interfaces in one interfacial supercell.

Further, the formed interfaces structures are totally relaxed for the external stress‐free states, Pristine A and B structures with similar atomic number and interfacial geometry are relaxed for interface normal direction (z) with static strain in‐plane lattice vectors (x and y), then interfacial energy is,

(16) $$\sigma =E_{AB\left(xyz\right)}-N_{A}E_{A\left(z\right)}-N_{B}E_{B\left(z\right)}/2S$$

where *E*_*AB(xyz)*_ is the total energy for interfacial structures. *E*_*A(z)*_ and *E*_*B(z)*_ are the energy per atomic layer of the pure A and B bulk structures, *N*_*A*_ and *N*_*B*_ are the atomic numbers of A and B interfacial supercells, respectively. The elastic strain energy is

(17) $$E_{\mathrm{Elastic}}=E_{f}/V-2S\sigma /V$$

Work adhesion for the interface is

(18) $$W_{\mathrm{Adhesion}}=\gamma_{A}+\gamma_{B}-\sigma_{AB}$$

where *γ_A_* and *γ_B_* are the surface energies and *σ_AB_* the interfacial energy of the A/B interface.

**Figure 5:**
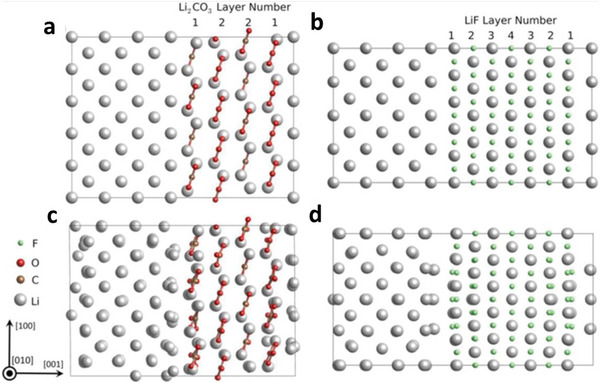
Atomic structures. a) as‐designed and (b) totally relaxed supercells of Li(001)/Li_2_CO_3_(001) interfaces, (c) as‐designed and (d) totally relaxed supercells of Li(001)/LiF(001) interface. Reproduced with permission.^[^ ^266^ ^]^ Copyright 2016, IOP publishing.

**Figure** **5** displays the interfaces of as‐formed (initial) and totally relaxed interfacial super cells (final) for Li(001)/Li_2_CO_3_(001) and Li(001)/LiF(001).^[^ ^266^ ^]^ Li_2_CO_3_/Li interface shows severe structural changes relative to LiF/Li, which illustrates the larger distortions of CO_3_ close to the interface region. The angle for the CO_3_ group and interface is denoted as A(CO_3_‐010) to evaluate the structural relaxation of the Li_2_CO_3_/Li interface. A(CO_3_‐010) displays a large disparity due to the mismatching of Li_2_CO_3_ and Li lattices. For interfacial supercell [Li(001)/Li_2_CO_3_(001)], the A(CO_3_‐010) varied from 16.8 to 22.7° for interface layer 1. Further, it converges to 20° for layer 2 closer to bulk Li_2_CO_3_ (18.6°). For interfacial supercell [Li(110)/Li_2_CO_3_(001)] the deviation of A(CO_3_‐010) is even higher, (i.e., 17.8 to 27.1° for layer 2). Under vacuum, A(CO_3_‐010) is closer to bulk values for layer 2, and the work function is united for >4 layers of Li_2_CO_3_. Large distortions for the relaxed Li(001)/Li_2_CO_3_(001) structures ascribed to prominently different lattice structures [Li metal (bcc) and Li_2_CO_3_ (monoclinic)]. For the cubic Li interface, CO_3_ tends to decrease the angle with minimizing the total energy. Notably, limited Li ions transport from SEs to anode during charging cycles, the nucleation of undesirable Li dendrites, and growth in SEI have been observed for Li_2_CO_3_/Li relative to those of LiF/Li interfaces. Yan et al.^[^ ^267^ ^]^ stated that the grain boundary resistance is formed for crystalline LLTO matrices interfaces with limiting Li‐migration due to structural and chemical deviations for random orientations of adjacent LLTO grains. Lattice mismatch in Li_0.33_La_0.56_TiO_3_ with NdGaO_3_ substrate for a‐ and b‐axes directions provides the anisotropy in the ionic conductivity.^[^ ^268^ ^]^ Lithiation‐induced volume changes will also illustrate lattice mismatch for electrode/SEs during cycling; ion mass transfer disrupts mechanical integrity for both interfaces. Interface lattice mismatch severely causes local structural distribution at the interface, decreasing the cells overall performance. The most promising approach to minimize the lattice mismatch is utilizing the high structural similarity materials in SEs and electrodes and constructing epitaxial interfaces.

### 6.5 Dendrites Growth

Metals (Li, Na, K, Zn, Mg, Al, Ca) are the ideal anode materials for battery chemistries due to their higher intrinsic capacities; however, dendrites formation severely influences the operation capability of their cells. The dominant cell failure mechanism is the penetration of dendrites along the grain boundaries. The probable mechanisms for dendrites nucleation for SPEs and SIEs are as follows. 1) Dendrites grow at the tip and penetrate through a soft part of SPEs, 2) lateral growth of dendrites and prolongs as of the electrode sites and SPEs, 3) subsurface structures triggered the formation of dendrites, 4) Redistribution of charges for metal/SPEs interface induces the dendrite formation. 5) Discontinuous interfaces contacts persuaded dendritic growth (i.e., surface microstructure, defects, voids, and density of SIEs), 6) grain boundary persuaded dendritic growth (i.e., grain boundaries create the metal propagation in the SIEs), 7) Electrons as of residual conductivities, pore surface, and oxygen framework encourages the metal clusters formation in the SIEs, and 8) Enriched electric field along the tips because of highly stable chemical interface among the SIEs and metals causes the dendritic growth (i.e., Interphase effect).

Brissot et al.^[^ ^269^ ^]^ reported the needle‐like dendrites at the tip during eventually increased electrodeposition time for symmetric Li|polymer|Li cells, which can penetrate the PEO‐based SPEs due to the high ionic concentration of electrolytes. Monroe and Newman^[^ ^270^ ^]^ explained the dendrite growth model based on the surface energy and tip‐curvature control for parallel Li|polymer|Li cells. Li deposition probably occurs for dendrite tips because of the faster accumulation of electric charges relative to smoother regions. Once activated, grown dendrites penetrate through SPEs even for low current density and enlarged distance among the electrodes. Considering the elastic deformation, all the factors, including the surface tension, deformable force across the interface, and compressive forces, contribute to the interfacial stability. The shear modulus of the separator (SPEs) is equal to the modulus of Li, which can form a stable interface. In contrast, if the shear modulus of SPE is two times higher relative to the Li, then dendritic growth will be mechanically suppressed. Dolle et al.^[^ ^271^ ^]^ reported lateral growth of dendrites for the Cu/polymer/Cu system, leading to delamination among the electrodes and SPEs. Harry et al.^[^ ^272^ ^]^ reported that the dendrite growth process for Li/SPEs reveals the reconstruction of volumes with dendrites buried under electrodes without residing in the electrolytes initially. Zhou et al.^[^ ^273^ ^]^ reported the redistribution of charges, in which lower Li transfer numbers and rapid depletion of anions in SPEs create the double‐layer electric field across the Li/SPEs interface, illustrating the decomposition of electrolytes, nucleation of dendrites, and interface instability. Cheng et al.^[^ ^274^ ^]^ reported the inter‐granular Li metal propagation via structure‐distributed grain boundaries for Li_6.25_Al_0.25_La_3_Zr_2_O_12_ SIEs owing to the larger grain boundary resistance, lower shear modulus, and stoichiometric variation than bulk grains. Yu and Siegel reported the transport properties, energetics, and composition for Li_7_La_3_Zr_2_O_12_ symmetric tilted grain boundaries. Li transfer in grain boundaries is highly challenging compared to bulk grains and highly sensitive to temperatures and boundary structures.^[^ ^275^ ^]^ Sastre et al.^[^ ^276^ ^]^ reported that the amorphous phase of Li_7_La_3_Zr_2_O_12_ creates the Li dendrite shield due to grain boundaries free microstructures. Li stoichiometry variation increases four times higher ion conductivity with minimal electronic transfer. Notably, the Li‐plating/stripping should be considered lower than those of critical current density (CCD), which is 0.5–1 mA cm^−2^. Beyond CCD exhibits dendritic growth and penetration via SEs. Insertion of buffer layers significantly improves the interfacial properties. Various types of buffer layers such as Al_2_O_3_, carbon/graphite, LiF, Li_3_N, Li_3_PO_4_, Li_3_NBO_4_, LiTaO_3_, BaTiO_3_, ZrO_2_, ZnO, Ge, Nb, Sn, Bi, Au, Mg, Al, SFC/CNT, Mg_3_N_2_, and Li_2_SiO_3_ have been reported as compatible materials for superior interfacial reactions that prevent the Li‐dendritic growth.^[^ ^277^, ^278^, ^279^, ^280^, ^281^, ^282^, ^283^, ^284^, ^285^, ^286^, ^287^, ^288^ ^]^

Hundekar et al.^[^ ^289^ ^]^ reported that the K‐metal undergoes hemispherical depositions for low current density (0.01 mA cm^−2^), a charge‐transfer controlled reaction. WI increase in current, the nuclei for dendritic growth initiates with diffusion‐controlled reactions. These dendrites are more densely packed, showing decreased diameter with current density. For 2 mA cm^−2^, the K morphology is smoother and non‐dendritic in nature. For 1.5 mA cm^−2^, partial dendritic structures are observed. Davidson et al.^[^ ^30^ ^]^ reported the formation of Mg dendrites with enriched Mg and a trace of Cl. Nano‐indentation exhibits that the Mg dendrites have an elastic modulus of 27.1 ± 2.8 GPa, which is extremely higher than Newman and Monroe criterion for shear modulus, showing penetration for polymers. Kwak et al.^[^ ^290^ ^]^ reported the operando visualization for Mg morphology evolution. Mg depositions microsized spherical particles from 50–83 µm diameter for 2 and 5 mA cm^−2^ current density. However, fatal Mg dendrites are observed for 10 mA cm^−2^. Mg dendrites are sparsely formed in micrometer‐scale branches, increasing with high current densities of 20 and 50 mA cm^−2^ with fast growth rates.

Al dendrites are typically formed due to inhomogeneous Al electrodeposition resulting from numerous nucleation sites that originated from disruptions in Al_2_O_3_ passivation under different electrolytes.^[^ ^291^, ^292^ ^]^ Unlike Li‐cells SEI, the Al_2_O_3_ is an inorganic phase, which inclines anisotropic cracks with lower Al redox potentials. AlCl_4_^−^ anions dominate the charge transport instead of Al^3+^, which unsurprisingly drops the ion transport efficiency in the electrolytes and cathodes.^[^ ^293^ ^]^ Al dendrites are topologically branched for 2D structures. The sluggish ion diffusion mechanism of anion‐type charge carriers with lower ion supply is challenging to address the moss‐like dendrites.^[^ ^294^, ^295^ ^]^ For Zn depositions, Zn ions closer to the Zn electrode surface transfer to nucleation sites with the action of the electric field. Obtained electrons from the Zn anode are deposited on the nucleation sites by overpowering the Zn nucleation energy barrier (i.e., overpotential > nucleation energy barrier). Zn ions will diffuse to energy‐favorable sites with forming Zn cores at lower current densities. For high currents, Zn ions display more considerable concentration gradients with uneven nucleation of Zn. In addition, the concentration of zinc ions near the zinc anode is related to the current density. When the current is high, the concentration gradient of zinc ions between the reaction zone and the bulk solution will occur, which is more conducive to the uneven nucleation of zinc. After nucleation, Zn ions are continuously reduced and plated for nucleation sites; Zn will accumulate on the electrode surface during cycling, which has random deposition due to concentration gradient and electric field. Such polarization shows the deviation in the potential for electrodes from equilibrium potential with reduction and accumulation of Zn for the tips of electrodes with higher electric field strength. Due to the abundance of charges over the tips, the uneven Zn depositions will be formed with boundaries, impurities, and dislocations defined as dendrites. Yufit et al.^[^ ^296^ ^]^ reported formation and dissolution kinetics for Zn dendrites, in which dendrites are initiated due to high local current density. Dendrites morphology strongly depends on the electrolyte kinetics (liquid: acid, alkaline, neutral, organic, solid, gel, etc.). Introducing artificial buffer layers among the electrode/electrolytes is a versatile strategy to diminish the interfacial issues for different metal chemistries from Mg, Na, Al, Zn, K, Ca, and Li, as understood from the above discussion (Table 2).^[^ ^297^, ^298^, ^299^, ^300^, ^301^, ^302^, ^303^, ^304^, ^305^, ^306^ ^]^

### 6.6 Li‐Immobilization Interlayers

Interlayer construction immobilizes transferable Li‐ions from the electrodes, which illustrates a loss in storage capacity. Chen et al.^[^ ^260^ ^]^ reported the formation of a Li‐immobilization interlayer for the Si/Li_3_PO_4_ interface by operando neutron depth profiling, in which Si transfers in the Li_3_PO_4_ to generate Li‐Si‐P‐O SEI. However, such an interlayer includes immobilized mobile Li‐ions unavailable for cycle operations. These interlayers typically occur under the lithiation process only. In principle, the Li‐immobilization interlayer will not influence transport kinetics if enough Li ions are available in SEs and the conduction of Li_3_PO_4_ by hopping kinetics. The Si/LiPON/LiCO batteries show the disordered interlayer at the interface between the LiCO and LiPON. LiPON deposits influence the disordered interlayer under N_2_‐supported Ar unfavorably compared to the Li_3_PO_4_ deposition on the LCO surface under Ar. Disordered interlayers containing Li_2_O and Li_2_O_2_ are poor conductive relative to LiPON, which degrades the SSBs' performances.^[^ ^307^, ^308^ ^]^ In summary Li‐immobilization interlayer is formulated due to electrochemical reactions at the interfaces that strongly influences by synthesis parameters and materials chemistry.

### 6.7 Current Collectors/Electrode Interfaces

Current collectors (CC)/electrodes interface (external circuit's electrical conductance) strongly influences the metal‐ion transport kinetics, including Li, Na, Zn, Al, K, Mg, Ca, etc. Primary requirements of current collectors: 1) Electrochemical stability‐ CC should be stable for reduction ad oxidation electrochemical reactions during discharge/charge processes. High voltage is typically favorable for high‐energy cells that need high/low electrochemical potential for cathodes/anodes. 2) Electrical conductivity‐ All batteries proceed by the sizeable electronic conductivity of CC. During operations, generated electrons transfer via CC to external circuits. It reveals the electrical conductivity of CC and CC/electrode interface illustrates the lower transfer of chemical/electrical energy in thermal runaway during cell charge/discharge steps, which displays high energy and capacity efficiencies. 3) Mechanical strength‐ typically, electrodes are prepared using a slurry coating, which means CC are mechanical supports. Under cell operations, the volume change of electrodes causes delamination or pulverization; thus, CC with high mechanical strength can retain the integrity of active materials. 4) Density‐ Generally, CC are electrochemically inactive materials, and utilized cell weight by CC is 20%. Thus low‐density CC is highly favorable to enhancing energy density. 5) Sustainability and cost.

**Figure 6:**
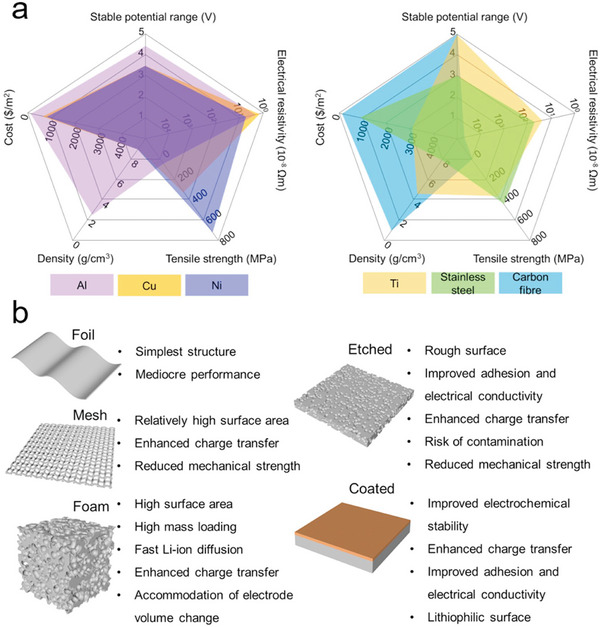
a) State‐of‐the‐art for various current collectors. b) Pros and cons for current collectors. Reproduced with permission.^[^ ^311^ ^]^ Copyright 2021, Elsevier.

There are different types of CCs, including Al (foil, mesh, foam, etched, carbon/graphene oxide‐coated, Mn and Al oxide composite coated, chromate‐coated, graphene‐coated for various cathodes (LCO, LFP, LNMO, NMC111, LMO, TiO_2_, NMC811, NMC622, NCA), **Figure** **6**). Kanamura et al.^[^ ^309^ ^]^ showed a comparison of LNMO for Al mesh and foil CC; however, both displayed 130 mAh g^−1^ capacity. Al foam allows excess mass loading of 42 mg cm^−2^ with an areal capacity of 7 mAh cm^−2^, three times higher relative to Al foil 12 mg cm^−2^ (2 mAh cm^−2^).^[^ ^310^ ^]^ Al CC thickness is also varied from 25 to 10 µm to obtain high energy, in which Sony LIBs reported 12 µm Al thickness shows gravimetric and volumetric energy of 246 Wh kg^−1^ and 665 Wh L^−1^, whereas 15 µm thick Al results in 196 Wh kg^−1^ and 552 Wh L^−1^. Low Al thickness severely affects the power density due to decreased heat transfer property and electrical conductivity.^[^ ^311^, ^312^ ^]^

Yoon et al.^[^ ^313^ ^]^ reported Al surface etching for mixture of NaOH, Na_2_CO_3_, C_6_H_11_NaO_7_ and NaOH, NaNO_3_, and C_6_H_11_NaO_7_ for 10 and 70s. It shows four times higher discharge capacity for high C‐rates than those of pristine Al, ascribed to increased adhesion among the CC and electrodes, decreasing the charge transfer resistance due to the high rough surface. Such strong adhesion avoids the peeling of cathodes and increases surface hydrophilicity. Crystal orientations and surface properties of CC will significantly modify the cell cycle operations. Wu et al.^[^ ^314^ ^]^ reported three types of Al current collectors, such as unetched Al, etched Al with carbon coating, and etched Al without carbon, in which they observed that etched Al with carbon layer has lower resistance and superior cycle capacity relative to those of other current collectors. Further, Cu and Cu with carbon coating reveal interfacial resistance and capacity of Li_4_Ti_5_O_12_ cathodes among the CC, and Li_4_Ti_5_O_12_ has the tradeoff as Cu‐C < etched‐Al‐C‐Cu < etched‐Al. Besides, Cu with conformal graphene facilitates better adhesion with electrodes.^[^ ^315^ ^]^ Nara et al.^[^ ^316^ ^]^ reported interface between the LCO and Al CC with/without carbon coating, in which pristine Al CC with LCO showed high resistance with poor contacts, and carbon coating with pressure had a superior interface with lower resistance. Shinde et al.^[^ ^191^ ^]^ reported carbon‐coated stainless steel (SS) mesh has superior interfacial performances and adhesion with cathode materials showing high cell capacity for Zn‐batteries (1–5 Ah scale).

Samsung and Sony show 10 and 14 µm Cu CC thickness that can display gravimetric and volumetric energy of 245, 196 Wh kg^−1^ and 657, 552 Wh L^−1^, respectively. Cu CCs are utilized in various forms such as foil, mesh, foam, etched, carbon‐, graphene‐, CuO‐, Ag‐, Ni‐, ZnO‐, and PVDF‐coated, in which mesh displays high performance due to fast charge transfer kinetics, reduced electrolyte/electrode interface impedance, accommodation of volume change, high surface area lowers the areal current density, and thickness of anode remains unchanged verifying mechanical stability.^[^ ^312^, ^317^ ^]^

Ni, Ti, SS, and carbonaceous materials are also reported as CC. Regarding electrochemical stability, Ti and carbon fibers have the largest voltage window (0–5 V versus Li/Li^+^), which can be utilized for both anode/cathode CCs. Al ranges from 0.5–5 V versus Li/Li^+^; however, alloying reaction with Li gets 0 V versus Li/Li^+^. Cu and Ni have stable windows for 3.5 V versus Li/Li, and SS has the smallest 0–3 V versus Li/Li^+^ window.^[^ ^311^ ^]^ Therefore, Al or carbon fibers and Cu, Ni, or SS are used for cathodes and anodes CCs, respectively, and can be utilized for different battery chemistries (Li, Na, Mg, K, Zn, Al, and Ca).

### 6.8 Thermal Instability

Thermal runaway induces exothermic thermochemical and electrochemical reactions due to increased battery temperature. The major parameters for thermal runaway are; 1) Electrical exploitation (over‐charge or discharge), 2) Thermal explosion, 3) Mechanical exploitation, and 4) Internal short circuit. Spotnitz and Franklin reported a 1‐D model to evaluate the thermal runaway exothermic reactions as; SEI layer decomposition occurs for 90–120 °C, intercalation reactions with electrolytes >120 °C or fluorinated binders, decomposition of electrolytes (>200 °C), decomposition of cathode active materials, overcharge provides the reaction of Li metal to electrolyte, binder reactions with Li, and discharge of cells releases heat owing to ohmic hesitance, overpotential, and entropy change.^[^ ^318^ ^]^ Utilizing thermally responsive materials such as separators, electrolytes, and temperature‐sensitive electrodes for retaining safety issues is the most promising approach. PM‐PP, PSS, PBI/PE/PBI, PE‐coated PET, EVA‐coated PP, PS‐co‐PBA@SiO_2_ coated PP, PE coated anode, paraffin coated electrodes, SEPs/SEs, carbon‐black‐polymer, or Ni‐PVDF composites, P3OT coated CC with ion‐blocking, phase transition, and PTC effect kinetics are reported materials to resolve thermal‐responses up to 100–140 °C.^[^ ^319^, ^320^, ^321^, ^322^ ^]^

### 6.9 Chemical Reactivity of SEI

SEI is mainly derived by metal and electrolytes electrochemical reactions, which composition depends on the utilized types of electrolytes. Typically, SEI is a mixture of inorganic metal salts (Li, Mg, Na, K, Zn, or others) and organic species; however, their precise distribution and stoichiometries remain a mystery. For example, Li_2_O, LiF, Li_2_CO_3_, Li_2_S, and Li_3_N are the Li‐salts, in which LiF is magical salt to realize stable anodes.^[^ ^323^ ^]^ Analysis of SEI is highly challenging due to the thickness (10–100 nm) and sensitivity of SEI for high‐energy radiations.^[^ ^324^ ^]^ Li_2_O crystal of SEI acts as nucleophilic agents for the initiation of decomposition of ester electrolytes solvents that explains the mosaic structure of SEI. In contrast, alkyl carbonate Li salts decompose to Li_2_CO_3,_ influencing SEI chemical stability. Ideal SEI criteria in terms of kinetics and thermodynamics are as follows. SEI should retain superior metal‐ion conductivity for kinetics prospects to enable fast metal‐ions transfer kinetics and redox reactions (dissolution or deposition). Homogeneous large ion conductivity is required for uniform chemical distribution and thickness. It can result in spherical or columnar structures commanding minimal stress for SEI with breakage of SEI and formation of dead metals. SEI should possess thermal and chemical stability during cycle operations for thermodynamics prospects. It should have considerable corrosion resistance against electrolytes and high temperatures and be thermodynamically stable against decomposition to maintain cell capability for extended operations.

The interface between the SEs and metals was predicted to be more stable than those of liquid electrolytes. For example, beta‐alumina is utilized SEs for high‐temperature Na‐S and ZEBRA cells, which has stable SEI.^[^ ^325^ ^]^ Thus, using beta‐alumina SEs against molten Na for low temperatures is interesting. The chemical stability windows of SEs are quite limited, so they can also decompose in contact with Li. In general, Li and SEs include the generation of Li_2_S, Li_3_P, or Li_2_O binary compounds with the reduction of metal cations. For example, Ti or Ge in NASICON, Li_2_S, Li_3_P, Ge/Ge_4_Li_15_ in LGPS, Li_2_S in Li_3_PS_4_ or Li_6_PS_5_Cl, and In, Y, LiCl in Li_3_InCl_6_ or Li_3_YCl_6_.^[^ ^10^, ^326^, ^327^, ^328^, ^329^, ^330^, ^331^ ^]^ Comparatively, oxide SEs have larger EW.^[^ ^332^ ^]^ Theoretically, three types of interfaces for Li and SEs are prominent: 1) thermodynamically stable interface (satisfied with binary compounds Li_2_S, Li_3_P, and Li_3_N only; complex electrodes readily reacts with Li owing to high reducing power). 2) Unstable interfaces (continuous degradation due to mixed ion and electron transfer kinetics). 3) Unstable interfaces that are kinetically stabilized but Li ions conductive. For type 3, the interface requires mechanical and chemical compatibility among the Li and SEs. Favorable types form direct SEI in an ideal scenario (e.g., cubic‐Li_7−3_ *_x_* Al*_x_* La_3_Zr_2_O_12_). The c‐LLZO displays limited reactions with Li regarding developing a tetragonal‐LLZO interface, which prolongs merely over 5 unit cells.^[^ ^38^, ^333^ ^]^ Interface engineering with ex‐situ or in‐situ coatings is a significant approach to enhancing the compatibility of SEs to cathode materials.

## 7 Anode Interface Chemistry

In contrast to commercial LIBs, the battery chemistries with promising high power, energy, and sustainability with low cost are significantly challenging. The alternate working ions are Na, K, Mg, Al, Zn, and Ca. A fundamental understanding of interface chemistries with suitable electrolytes is critical to delve into various anode reactions.

### 7.1 Monovalent‐Ions Battery Technologies

#### 7.1.1 Li‐Metal Batteries (LMBs)

All‐solid‐state LMBs are considered one of the most prominent next‐generation energy storage technology owing to high energy densities relative to LIBs. LMBs with SEs have two types, inorganic and organic (polymer), in which inorganic SEs are fireproof, stable, and have relatively high EW compared to polymer or liquids. Electrode's volume change, a low mass ratio of active materials, poor cycle life, and large interface impedance are the fundamental obstacles for SSBs. For higher ion‐conductivity of electrode/SEs interface, the diffusion behavior, and ion transport across the interface, mechanical and structural parameters of SEI and SEs with atomic‐level understanding are essential. Typically, the elasticity for SEs and electrodes has a prominent influence over physical/chemical contacts for their interfaces. In practice, the complex SEI at the metal surface influences the ion‐conduction characteristics of the SEs/anode interface; thus, proper strategies for the protection of anodes are critically necessary to enhance cell performance. After the Li–TiS_2_ system, Li‐metal has been considered one of the ultimate anodes due to its theoretical capacity (3860 mAh g^−1^) and minimal redox potential.^[^ ^334^, ^335^, ^336^ ^]^ However, the severe dendritic growth causes poor interface stability and safety concerns. In the 2000s,^[^ ^337^ ^]^ it was concluded that Li metal is not suitable anode material for rechargeable batteries; however, recently, the research community has revival interest in high‐energy Li‐metal batteries. Typically, preventing dendritic growth is extremely problematic since it initiates from the nanoscale roughness of surfaces, in which the entered Li ions deposit preferentially over sensible piercing tips and protrude the morphology for intrinsic Li‐metal surfaces. Several reports stated that the formation of elastic SEI has a great extent for blocking the open spaces and exerting pressures to suppress interfacial parasitic reactions.

**Figure 7:**
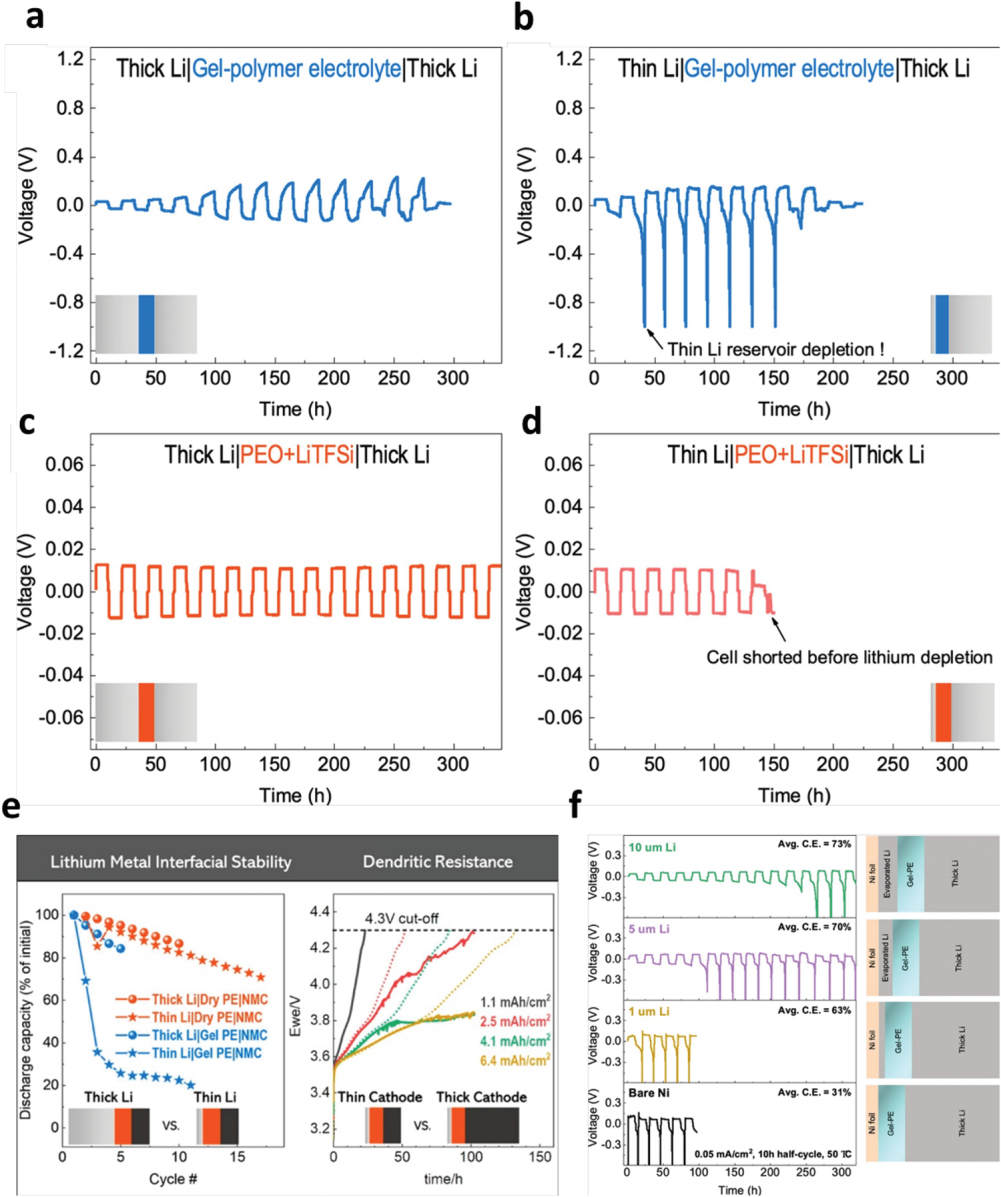
a, c) Voltage profile for Li plate/strip under GPE and SPEs (PEO+LiTFSi) electrolytes for thick ≈600 µm Li. (b, d) Voltage profile for Li plate/strip under GPE and SPEs (PEO+LiTFSi) electrolytes for thin ≈13 µm Li. Current density 0.05 mA cm^−2^. e) Discharge capacity with cycle numbers (left) and charge voltage profiles of Li/PEO+LiTFSi/NMC811 (right). f) Quantification of Li losses. Reproduced with permission.^[^ ^338^ ^]^ Copyright 2021, American Chemical Society.

LMBs with SPEs have benefits for ease of fabrication with flexibility and safety, in which instability for electrodes/SEs interface and poor electrochemical working conditions are major restrictions. Polymers displayed in Table 1 are the several options; however, their poor electrochemical stability with high voltage cathodes such as LCO, LMNO, and NMC cannot show the proper pairing. Mixed SPEs and SIEs (polymer/inorganic/polymer or polymer/inorganic) offer improvement for ASS LMBs due to adjusting the double‐layer electric field at the interface with blocking anions transport. Sahore et al.^[^ ^338^ ^]^ evaluated Li metal stability under PEO+LiTFSI SPEs and PEO‐PEGDME‐LiTFSI‐LiAlTiPO_4_ GPEs (**Figure** **7**a–d). Thick Li//Li (600 µm) cells display an increased overpotential of 0.03–0.2 V without exceeding the cutoff limit of ±1 V. Upon replacing thin Li (10 µm), the 2^nd^ strip cycle itself reaches the cutoff limit of ‐1 V with depletion of entire thin Li for SEI formation owing to reductive reactions of GPE with creating dead Li, which illustrates the incompatibility of GPE for realistic LMBs. For SPEs, thick and thin Li displays minimal impedance growth without increasing overpotentials. Li//PEO‐LiTFSI//NMC811 cells display an increase in areal capacity from 1.1–6.4 mAh cm^−2^ among the 3–4.3 V versus Li/Li^+^ illustrating low capacity (1.1 mAh cm^−2^) reaches cutoff of 4.3 V for 1^st^ charge without soft/hard shorts. For > 4 mAh cm^−2^ capacity shows severe shorts without a higher state of charge due to poor rate of de‐/intercalation of Li (Figure 7e). For pristine Ni, overpotential reached for cutoff at 1^st^ cycle, 1, 5, and 10 µm thick Li reservoir hits 1^st^, 5^th^, and 12^th^ strip cycles with CEs of 63, 70 and 73%, which is extremely poor for practical cells (Figure 7f).

Wang et al.^[^ ^339^ ^]^ proposed high‐strength and ultrathin (10 µm) poly(methyl methacrylate)–polystyrene based m‐PPL SPEs with ion‐conductance of 34.84 mS cm^−1^ and 103 MPa shear modulus. Li//Li cells demonstrate stable operations for 1500 h for 60 °C verifying better interface compatibility with Li metal. Theoretical calculations clarify the coordination numbers of Li^+^‐ions nearby (trifluoromethanesulfonyl)imide (TFSI^−^) pairs and ethylene‐oxide chains declines the Li/polymer interface facilitating the Li^+^ transfer via polymeric host for PEO‐black‐phosphorus (BP) CPEs. Adsorption of LiTFSI molecules at BP surface indicates the fading of Li and N atomic bindings with increasing dissociation of Li^+^ ions.^[^ ^340^ ^]^

**Figure 8:**
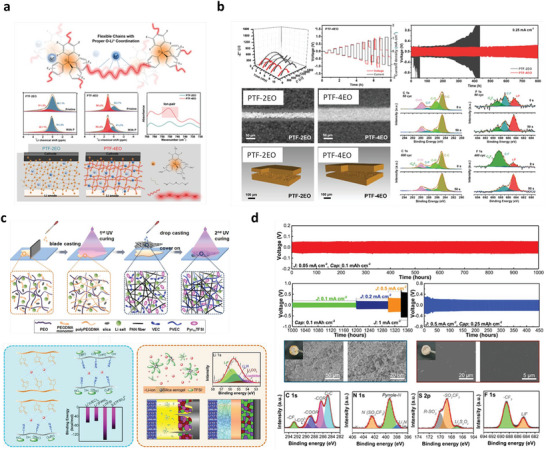
a) Structures characterizations for PTF‐4EO and PTF‐2EO. The blue or light blue balls show mobile Li^+^ ions in SPEs. b) Electrochemical and structural performances under PTF‐4EO and PTF‐2EO SPEs and XPS spectra of Li‐metal under PTF‐4EO with/without 50 s Ar etching. Reproduced with permission.^[^ ^341^ ^]^ Copyright 2022, Wiley VCH. c) Synthesis processes and schematic illustration for three possible polymers. Li^+^ interaction modes, solvation structures in PSPE, and SEI compositions DLPE and PEO/LiTFSI. The mosaic segments specify SEI constituents for various cells. Green block – LiF; purple block – Li_3_N; wine block – Li_2_CO_3_; pink – Li carbides. d) Electrochemical performances Li//Li cells under DLPE or PEO/LiTFSI and XPS results of Li metal surface with DLPEs. Reproduced with permission.^[^ ^342^ ^]^ Copyright 2020, Elsevier.

**Figure** **8**a reports the fabrication of single‐ion SPEs by crosslinking lithium tetrakis(4‐(chloromethyl)−2,3,5,6‐tetrafluorophenyl)borate salt with tetraethylene glycol (PTF‐4EO). It has *σ_Li_^+^* 0.35 mS cm^−1^, EW >4.8 V, and Li‐ion transfer number (t_Li_^+^ ≈0.92).^[^ ^341^ ^]^ The ssMAS NMR demonstrates the 4EO enables diamondoid networks with stable coordination of Li^+^‐O for Li^+^‐transfer kinetics. Li//PTF‐4EO//Li cells show stable interface resistance (760 Ω) relative to those of Li//PTF‐2EO//Li (Figure 8b). Mechanical strength and dimension stability verify the inhibition of Li dendrites from 0.1–1.5 mA cm^−2^. Nondestructive synchrotron X‐ray tomographs display a uniform interface without dendrites for PTF‐4EO, whereas rough with granular dead Li (dendrites) for PTF‐2EO verifies no effective media for Li deposition in PTF‐2EO. XPS spectra of C 1s and F 1s display the C‐O (286.3 eV), C‐F (286.3 and 687.1 eV), and LiF (684.6 eV) due to chemical reactions among the [B(C_6_F_4_)_4_]^−^ and Li. The ‐C_6_F_4_ (688.9 eV) specifies the direct incorporation of [B(C_6_F_4_)_4_]^−^ anions for the SEI layer, illustrating faster Li^+^ transfer for Li/PTF‐4EO interfaces. Enhanced intensity of LiF and decreased intensity of C═O and –C_6_F_6_ after Ar etching implies the inhibition decomposition of PTF‐4EO with LiF formation. Such robust LiF‐rich Janus SEI renders long‐operations for Li‐metal anodes.

**Figure 9:**
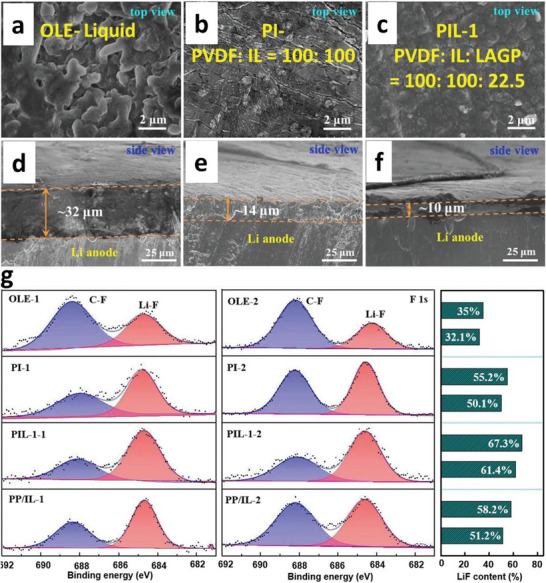
SEM images of cycled Li anodes (a, d) OLE (liquid electrolyte), (b, e) PI (PVDF+IL), and (c, f) PIL‐1 (PVDF+IL+LAGP) electrolytes, respectively. g) F 1s XPS spectra for Li‐metal for diverse electrolytes. Reproduced with permission.^[^ ^344^ ^]^ Copyright 2021, Elsevier.

Figure 8c explains the fabrication of double–layer polymer electrolyte (DLPE), including poly (ethylene oxide)–silica aerogel polymer electrolyte (PSPE) and poly(vinylethylene carbonate)–ionic liquid polymer electrolyte (PIPE).^[^ ^342^ ^]^ The weaker interactions among the PAN/PVEC and Li‐ions promote faster Li‐ion transfer due to the release of high free Li‐ions from [Li(TFSI)_4_]^3−^ complex. Such coupling/decoupling balances for Li^+^…TFSI^−^ and Li^+^…PAN/PVEC facilitating the superior Li‐ion‐conductance for DLPE. PSPE with Li realizes regular distributions for immobilized anions for the uniform flux of Li‐ions. Li 1s XPS displays enriched cubic‐LiF and layered‐Li_3_N SEI phases, which vindicates reversible and stable Li‐metal anode compared to those of crack states of PEO or other SPEs. Li//DLPEs//Li cells demonstrate stable polarizations from 0.1 to 1 mA cm^−2^ owing to regulation of SEI species with SCL and Li nucleation/deposition (Figure 8d). DLPE displays high mechanical properties such as stress/strain of 5.70 MPa/34.3% relative to those of PEO/LiTFSI (1.28 MPa, 379%) and pristine PAN fibers (4.25 MPa, 30%). SEM images of Li anodes exhibit dense and smoother metal deposits (450 µm) in which silica aerogel and pyrrolidinium contribute synergistically. Li||PEO/LiTFSI||Li displays severe needles/tree‐like dendrites and bulky voids influencing parasitic chemical reactions. XPS for Li//DLPE interface demonstrates Li_3_N, LiF, LixSyOz, and Li–alkylide peaks for 397.8, 684.8, 166.8, and 288.9 eV. The dominance of Li_3_N and LiF obtains more stable SEI from DLPE‐based LMBs than those of Li_2_CO_3_ and Li‐carbides‐based PEO‐based SPEs. Overall, SPEs have several limitations in terms of Li‐transport kinetics, poor conductivity, EW, rate capacity, physical‐chemical properties for stable Li‐electrodepositions, and poor interface for high current density; therefore, the SEs with superior properties are critically required beyond SPEs. He et al.^[^ ^343^ ^]^ presented ultrathin 4.2 µm bilayer SPE (UFF/PEO/PAN/LiTFSI SSE) with porous ceramic frameworks and double‐layer Li^+^‐conducting polymer with 0.068 mS cm^−1^ ion‐conductance and *t_Li_^+^*  ≈0.5. UFF defines the ultrathin, fire‐proof framework of ceramics. UFF improves mechanical properties (shear modulus of 175 GPa) and inhibits Li dendrites penetration during operations. Li//Li cells display 4 mAh cm^−2^ capacity with 99.3% CEs at 1 mA cm^−2^. Li anode SEM shows a rough surface with shorter grains composed of Li_2_CO_3_ and lithium oxides and dead Li with a 32 µm thickness under organic liquid electrolytes (OLE). But, Li anodes with PI and PIL‐1 (PVDF+IL+LAGP (Li_1.5_Al_0.5_Ge_1.5_(PO_4_)_3_):: 100:100:22.5) electrolytes exhibit relatively compact surfaces, whereas cracks and bumps are seen for PI electrolytes (**Figure** **9**a–f).^[^ ^344^ ^]^ PIL‐1 displayed 10 µm Li deposits implying a significant role of PIL‐1 in restraining Li dendrites. PIL‐1 demonstrates higher LIF content (61.4% and 67.3%) relative to those of OLE (32.1% and 35%) and PI (50.1% and 55.2%) in accordance with F 1s signal to LiF (Figure 9g). It is well‐known that LiF phase is more favorable for SEI and inhibition of Li dendritic growth. LiF shows superiority for regulation of Li^+^ transport due to lower diffusion energy barriers and high surface energies of Li^+^. Besides, the insulating character of LiF effectively obstructs the tunneling of electrons through SEI. Thus, LiF‐enriched SEI anticipates the optimal Li electrodepositions and prolonged operational life.

**Figure 10:**
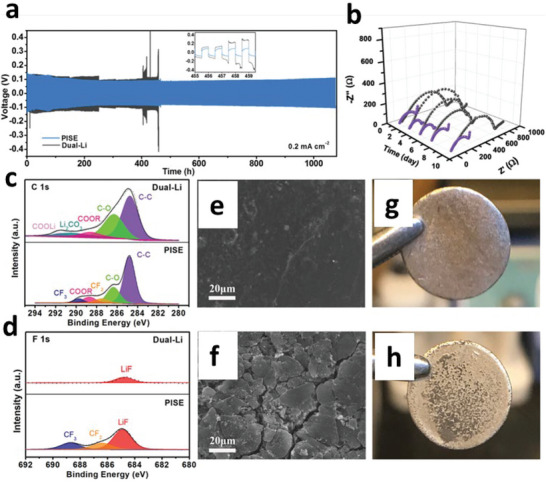
a) Voltage profiles for Li//PISE//Li and Li//Dual‐Li//Li symmetric cells. b) EIS spectra for Li//PISE//Li and Li//Dual‐Li//Li. XPS spectra of c) C 1s and d) F1s for cycled Li metal for Li//PISE//Li and Li//Dual‐Li//Li. SEM image for Li after plate/strip e) 1100 h for Li//PISE//Li and f) 460 h in Li//Dual‐Li//Li cells. Photograph of Li metal for g) Li//PISE//Li and h) Li//Dual‐Li//Li cells. Reproduced with permission.^[^ ^345^ ^]^ Copyright 2021, Wiley VCH.

**Figure** **10** demonstrates interface compatibility for Li/PISE,^[^ ^345^ ^]^ which is prepared using poly(methyl vinyl ether‐alt‐maleic anhydride) (PME) and single‐ion lithiated polyvinyl formal (LiPVFM)/lithium bis(trifluoromethylsulfonyl)imide (LiTFSI) composite salts (defined as polymer‐in‐salt solid electrolyte, PISE). PISE exhibits *σ_Li_^+^* ≈0.357 mS cm^−1^ and t_Li_^+^ ≈0.62. PISE demonstrates the uniform Li plating/stripping over 1100 h with stable polarization voltage. In contrast, dual‐ion displays voltage fluctuations after 400 h and drops for 460 h that illustrate penetration of Li dendrites (Figure 10a). Excellent mechanical strength (3.3 GPa) and *t_Li_^+^* of PISE sufficiently diminish the Li dendrites growth, which suggests good interfacial stability. EIS shows no obvious change for interface resistances even after 10 days (Figure 10b). Flexible SEI for Li/PISE interfaces with stronger LiF (684.9 eV) phase, high ion‐conductance, and flat and shiny surface confirms the uniform Li plate/stripping (Figure 10c–h). PISE SPEs are hard to peel off from cycled Li even after several washings, which implies ultra‐tight physical/chemical contacts.

**Figure 11:**
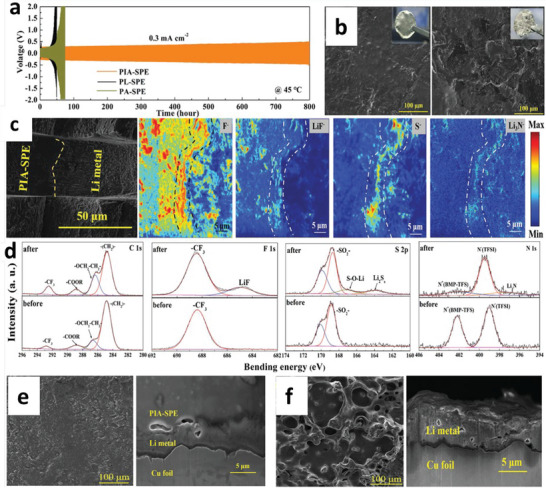
a) Symmetric Li cells performance with PIA‐SPE, PL‐SPE, and PA‐SPE. b) SEM images of cycled Li for PIA‐SPE (left) and PL‐SPE (right). Photographs are in the inset. c) SEM image and TOF‐SIMS maps of the Li/PIA‐SPE for Li/Li cell after cycling. d) XPS spectra of C 1s, F 1s, S 2p, and N 1s of PIA‐SPE interface with/without cycling. e) Surface and cross‐section SEM images of lithium deposition morphology on Cu foil of the asymmetric Li/Cu cell with PIA‐SPE after 1 h and f) commercial liquid electrolytes. Reproduced under the terms of a Creative Commons CC BY 4.0 license.^[^ ^346^ ^]^ Copyright 2022, Wiley VCH.

**Figure** **11** proposed the structural evolution of Li/SPEs interface under the polycaprolactone diol (PCL)/LiTFSI/IL/Al_2_O_3_ (10:4:4:1; PIA‐SPE; 0.089 mS cm^−1^; *T_g_* = −57.7 °C), PCL/LiTFSI/IL (10:4:4; PI‐SPE; 0.038 mS cm^−1^; *T_g_* = −58.2 °C), and PCL/LiTFSI (10:4; PL‐SPE; 0.001 mS cm^−1^, *T_g_* = −51.7 °C) electrolytes.^[^ ^346^ ^]^ Li with PIA‐SPE displays stable polarization voltage for 800 h, whereas PA‐SPE and PL‐SPE degrades for 50 h, illustrating the effect of IL for interface stability. SEM and TOF‐SIMS images display the localization of F^−^, LiF^−^, S^−^, and Li_3_N^−^ phases over a dense and smooth interface (Figure 11a–c). Li metal surface with PIA‐SPE shows no noticeable difference in color and flat and smooth morphology even after 100 cycles (Figure 11a). In contrast, PL‐SPE show dimmed coloration and mossy Li with sharp particles ascribed to dead Li or dendrites that responses the inferior Li plate/strip processes (Figure 11b). TOF‐SIMS (Figure 11c) maps display localization of F^−^, LiF^−^, S^−^, and Li_3_N^−^ over the interface. The IL reaction with Li metal forms an artificial SEI layer. The chemical states of F 1s, S 2p, and N 1s exhibit significant distribution. F 1s spectra show the 684.9 and 688.4 eV peaks of LiF and –CF_3_ of TFSI^−^. LiF promotes the ionic carrier concentration and limits electrical transport, contributing to a stable interface (Figure 11d). S 2p exhibits peaks for 167.3 and 163.5 for S═O after lithiation with in situ formed Li_2_S_x_ and 168.7 and 170.1 eV for S═O of pristine TFSI. The 399.1 and 402.2 eV peaks in N 1s XPS are ascribed to the N^−^ in TFSI^−^ and N^+^ in IL, respectively. IL surface has the formation of Li_3_N phase at 397.2 eV. Asymmetric Li/Cu cells with PIA‐SPE display uniform deposits without dendrites in contrast to liquid electrolytes (Figure 11e,f). Li with EMIM+‐PMMA (PIL) based SPEs explains the charge‐discharge voltage gap of 0.12 V, with plate/stripping over 1600 h with micro‐short circuits. PIL shows close contact with Li with a uniform thickness of ≈27 µm deposits.^[^ ^347^ ^]^ Yuan et al.^[^ ^348^ ^]^ reported flexible thin 16 µm high‐strength CPEs with *σ_Li_^+^* ≈0.1 mS cm^−1^, *t_Li_^+^* ≈0.71) obtaining stable Li/CPEs interface for 1000 h. LCO/Li SSBs display 76.1% capacity retention from 145.3 to 110.6 mAh g^−1^. LiNO_3_ was mixed in CPE as Li metal is incompatible with aliphatic succinonitrile to improve interface stability.

**Figure 12:**
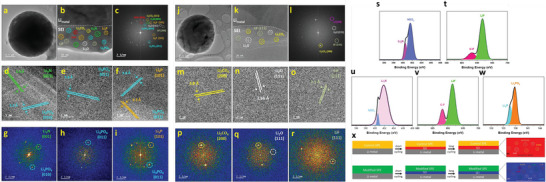
Li interface with P‐SPE. a) Cryo‐TEM image. b) HRTEM of the SEI and c) their FFT pattern. HRTEM images and FFT patterns of Li_3_N (d, g), Li_3_PO_4_ (e, h), and Li_3_P (f, i) for the SEI layer. Li interface with C‐SPE. j) Cryo‐TEM image. k) HRTEM of the SEI and l) the FFT pattern. HRTEM and FFT pattern of Li_2_CO_3_ (m, p), Li_2_O (n, q), and LiF (o, r) for SEI layer. XPS analysis of Li//LFP with C‐SPE and P‐SPE after C‐D 100 cycles. (s,t) C‐SPE shows Li_3_N and LiF phases in the SEI, and (u,v,w) P‐SPE shows Li_3_N, LiF, Li_3_P, and Li_3_PO_4_ phases in the SEI layer. (x) Illustrations for Li platting with SEI formation under C‐SPE and P‐SPE. Reproduced with permission.^[^ ^349^ ^]^ Copyright 2023, Elsevier.

**Figure** **12** displays the SEI analysis using cryo‐TEM under PEO‐LiTFSI SPE (C‐SPE) and phosphazene‐modified PEO‐LiTFSI SPE (P‐SPE) with Li anodes. SEI for Li/P‐SPE interface demonstrates mosaic structure with Li_2_O, LiOH, Li_2_CO_3_, LiF, Li_3_N, Li_3_P, and Li_3_PO_4_ phases, which are known for high Li conductance and superior interface compatibility. FFT patterns display that Li_3_N, Li_3_P, and Li_3_PO_4_ have an interplanar spacing of 3.8 Å, 3.3 Å, 3.6 Å, and 5.2 Å with crystal reflections of (001), (101), (010) and (011), respectively (Figure 12a–i).^[^ ^349^ ^]^ P‐SPE consists of abundant crystalline phases relative to those of C‐SPE. The superior stability of Li with modified SEI components can boost the operational life of LMBs. SEI with C‐SPE displays mosaic nanostructures where inorganic Li crystals are implanted inside amorphous organic/polymeric phases. HRTEM and FFT comprise the presence of Li_2_O, LiF, and Li_2_CO_3_ by matching the corresponding lattice spacings of 2.3 Å, 3.8 Å, and 2.66 Å, consistent well with (111), (111), and (200) planes, respectively (Figure 12j–r). XPS displays more enriched LiF and Li_3_N for P‐SPE than C‐SPE, like TEM, illustrating fast interfacial kinetics (Figure 12s–w). LiF‐rich and Li_3_N‐rich interfaces have slow energy barriers for diffusion with promoting homogenous Li‐ion flux and transports (Figure 12x). Further, the bidirectional functional polymer electrolytes (BDFPE) with *σ_Li_^+^* ≈0.58 mS cm^−1^, *t_Li_^+^* ≈0.69, and 1800 h dendrite‐free stable depositions for 1 mAh cm^−2^ and 1 mA cm^−2^ have also reported by UV solidification processes. Li//BDFPE//NMC622 demonstrates the formation of stable CEI and F‐enriched SEI with favorable features for interfacial protection.^[^ ^350^ ^]^

**Figure 13:**
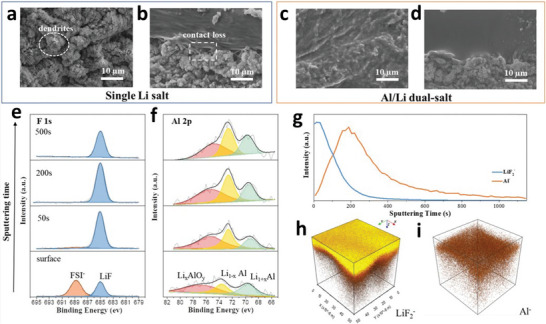
Li metal surface SEM images for (a) single Li salt and (c) Al/Li dual‐salt. Cross‐view SEM images for the Li/electrolyte interface for (b) single Li salt and (d) Al/Li dual‐salt. XPS spectra for the sputtering time of (e) F 1 s and (f) Al 2p of Li metal surface for dual‐salt. (g) TOF‐SIMS depth profiles for SEI layer. (h, i) 3D TOF‐SIMS depth profiles. Reproduced with permission.^[^ ^351^ ^]^ Copyright 2022, Elsevier.

Li metal deposits under PVDF‐PEO/LiTFSI (FPEO) with/without dual‐salt of Al/Li have been reported (**Figure** **13**).^[^ ^351^ ^]^ Li metal for single Li‐salt displays dendrites with rough (voids/cracks) topography, whereas dual‐salts have smooth dendrites‐free Li deposits. FPEO firmly adheres to Li after cycling, providing a favorable adhesive interface that alleviates Li dendritic growth. The residual Li‐salt peak (688.9 eV) disappears after sputtering, whereas 685.1 eV peak of LiF increases with sputtering time up to 200 s and then decreases for 500 s, which indicates a LiF‐rich SEI layer. Al^3+^ and Al_2_O_3_ peaks also disappear upon sputtering. The 75.8, 72.9, and 70.0 eV peaks of Li_x_AlO_y_, Li_1−x_Al, and Li_1+x_Al species initiate after Al reduction during lithiation reactions. Increased intensity of Li‐Al alloy signals after sputtering indicates an alloy‐rich layer at the bottom of SEI. TOF‐SIMS profiles show a transition of LiF‐rich layer to Li–Al‐rich layer, verifying the 3D renderings with lithiophilic–lithiophobic gradient SEI that can drastically enhance the Li/SPE interface. Overall, the capacities, rate performances, operating temperature, and cycle life for CPEs are inferior, ranging from 0.01 to 0.2 C, which cannot meet the requirements of high‐energy applications.

Obtaining high‐energy/power and long cycle life compared to commercial LIBs with lower cost and high safety is the future target of LMBs; however, it is a highly formidable undertaking. Fundamental requirements to achieve these conditions are good solid/solid interfaces with exceptional ion transport across interfaces, solid/solid interface stability, and wetting properties. Liu et al.^[^ ^352^ ^]^ proposed Janus interface stability for NASICON‐type Li_1.5_Al_0.5_Ge_1.5_P_3_O_12_‐based SEs with the application of IL to anode sites and adiponitrile to cathode sides (Li/LAGP/Li and Li/ASHE/LAGP/ASHE/Li). Janus interface rebuilds the cracks obtained by volume changes with retaining combined interfacial contacts and eliminating side reactions for Li and LAGP during operations. XPS reveals the LAGP consists of inorganic components (i.e., Li_2_O, Li_2_O_2_, and Li_2_CO_3_) as SEI elements; however, AGPE or ASHE with LAGP has LiF and LiCOOR phase arises in SEI with 1363–2549 MPa modulus, which illustrates robust SEI. Stabilizing Cl‐enriched SEs (Li_5.5_PS_4.5_Cl_1.5_, Li_6_PS_5_Cl, Li_6.5_PS_5.5_Cl_0.5_, and Li_7_P_3_S_11_) due to higher reaction strains is the severe limitation explained by theoretical calculations. Li_5.5_PS_4.5_Cl_1.5_ confirms the Li‐S‐P bonds of Li‐argyrodites (161.3 eV) and polysulfide doublet (163 eV) phases with bridging (‐S‐) and terminal sulfur bonds (P‐S_x_‐P). Besides, the presence of SO_3_^2−^ relates to the oxidization of Li_2_S phase. Low voltage shows Li_2_S, Li_x_P_y,_ and LiCl phases after decomposition.^[^ ^353^ ^]^

Yin et al.^[^ ^123^ ^]^ displays Li/Li_0.388_Ta_0.238_La_0.475_Cl_3_ prolonged interface compatibility with dense surface and homogeneous Li^+^ flux, in which the top surface of SEs has two states of Ta (Ta^5+^ and Ta^0^). The partial reduction of Ta causes different charging states that imply the electrochemical reduction of Ta° from 13.4 to 2.3% for 3 nm depth of the SE interphase layer. Such gradient reduction ascribes the passivation by electrically insulating the LiCl phase due to cation reduction in halide SEs, efficiently relieving interface strain and shielding SE at Li metal. Oxide SEs restrain the capacity and cycling life due to Li dendrite's growth along grain boundaries. In contrast, intimate interface contact of sulfide SEs promotes a better cycle life with low areal capacity. Most of chloride and halide SEs suffer for poor interface stability for Li with high overpotential and short cycle life [Li_2_ZrCl_6_ (LZC), Li_3_ScCl_6_ (LSC), G‐LPSC‐LGPS, graphite‐Li_5.5_PS_4.5_Cl_1.5_‐LGPS; Li_7_P_2_S_8_I (LPSI), Li_7_P_2.88_Nb_0.12_S_10.7_O_0.3_ (LPNSO), Li_5.5_PS_4.5_Cl_1.5_ (LPSC), Al_2_O_3_‐coated Li_7_La_2.75_Ca_0.25_Zr_1.75_Nb_0.25_O_12_ (A‐LLCZNO), Si‐coated Li_6.85_La_2.9_Ca_0.1_Zr_1.75_Nb_0.25_O_12_ (S‐LLCZNO), Ge‐coated Li_6.85_La_2.9_Ca_0.1_Zr_1.75_Nb_0.25_O_12_ (G‐LLCZNO)].^[^ ^123^, ^283^, ^354^, ^355^, ^356^, ^357^ ^]^

**Figure 14:**
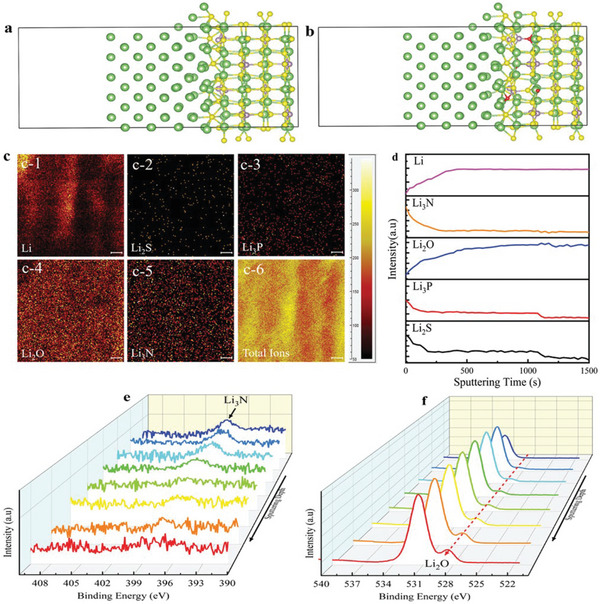
DFT interface structures for a) Li/Li_3_PS_4_ and b) Li/Li_2.96_P_0.98_S_3.92_O_0.06_–Li_3_N. c) Li‐anode surface analysis under Li/Li_2.96_P_0.98_S_3.92_O_0.06_–Li_3_N/Li cells. d) ToF‐SIMS depth profiles. XPS depth profiles of Li/Li_2.96_P_0.98_S_3.92_O_0.06_–Li_3_N interface e) N 1s and f) O 1s spectra with sputtering depth. Reproduced with permission.^[^ ^358^ ^]^ Copyright 2022, Wiley VCH.

Ahmad et al.^[^ ^358^ ^]^ proposed Li_2.96_P_0.98_S_3.92_O_0.06_‐Li_3_N SIEs, in which N and O replacement produce functional units of Li_3_N and POS_3_^3−^ that enable superior *σ_Li_^+^* ≈1.58 mS cm^−1^ and POS_3_^3−^ units in Li_2.96_P_0.98_S_3.92_O_0.06_‐Li_3_N prevents structural degradation under the moisture of 45–50% (**Figure** **14**a–c). Theoretical and experimental results state the Li_3_PS_4_ SEs follow the reaction of Li_3_PS_4_ + 8Li → 4Li_2_S + Li_3_P + Li*_x_* P) to formulate in situ SEI among the Li and Li_3_PS_4_. The reduced phases of Li_2_S, Li_3_P, and other intermediates Li*_x_* P are incompetent for active SEI owing to the higher electronic conductivity of Li_3_P/Li*_x_* P, which implies uneven Li electrodeposits with higher Li‐dendrites.^[^ ^359^, ^360^ ^]^ Li//LiPS(100) interface displays S‐atoms for 1^st^ layer of LiPS shifts to Li‐metal with forming Li_2_S at the interface, which illustrates the reformation of the top 3 layers of Li. Besides, three Li and LiPS interface layers did not observe lattice distortion. In contrast, O‐LiPS/Li interface implies Li‐O with a bond length of 2.1 Å (i.e., 0.3 Å smaller for Li‐S), which reveals strong interactions for Li‐O than those of Li‐S (similar to pristine Li_3_PS_4_). ToF‐SIMS and XPS profiles (Figure 14d–f) reveal the formation of pre‐SEI enriched with thermodynamically stable Li_2_O and Li_3_N species (minor phases Li, Li_2_S, Li_3_P) at Li/Li_2.96_P_0.98_S_3.92_O_0.06_–Li_3_N interface, which suppresses the interface reactions and dendritic growth of Li inside Li_2.96_P_0.98_S_3.92_O_0.06_–Li_3_N. The peripheral Li_2.96_P_0.98_S_3.92_O_0.06_–Li_3_N SIEs are stable for Li‐anode, revealed by no shifts in the binding energy. Li et al.^[^ ^361^ ^]^ reported the influence of 0.5 wt% Li_2_S loading for LPS SEs. During cycling, the Li_3_P, P_2_S_7_^4−^ and PS_4_^3−^ phases have been observed that indicate a reaction with Li in LPS. Li_2_S loading reduces the diffusion energy barrier of LPS by promoting stable depositions.

Incorporation of piezo‐/ferroelectric BaTiO_3_ in CSEs (Li_6.7_La_3_Zr_2_Al_0.1_O_12_ (LLZAO) + PEO + LiTFSI) is alternates strategy to improve CESs ion‐transport‐kinetics. BaTiO_3_ effectively reduces the driving forces for Li dendrites for high curvatures, whereas ferroelectricity decreases overpotential, illustrating good Li^+^ flux and deposits. Theoretical calculations show that the driving force manifests a bulging area rather than a planar interface. Activation overpotential significantly influences the magnitude and evolution of the driving force, illustrating lower the activation overpotentials – the smaller the driving forces. Piezo‐/ferroelectric fields regularize the activation overpotentials for guiding planar Li depositions.^[^ ^362^, ^363^ ^]^

**Figure 15:**
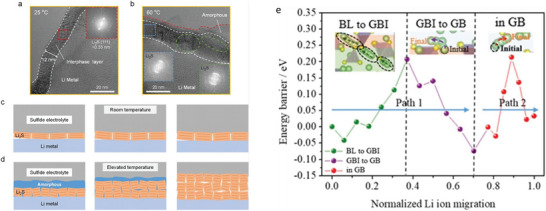
TEM images and FFT patterns (a) 25 °C and (b) 60 °C for Li/sulfide SEs. Bright diffraction spots are from the (111) plane of Li_2_S. Schematic illustration for interphase evolutions at (c) RT and (d) 60 °C. Reproduced with permission.^[^ ^364^ ^]^ Copyright 2022, American Chemical Society. e) Energy barriers for Li migration as variations with different regions. Reproduced with permission.^[^ ^374^ ^]^ Copyright 2023, American Chemical Society.

TEM images of Li//LPSCl verify the highly crystalline Li_2_S(111) phase with an interplanar spacing of 0.33 nm with 12 nm SEI thickness at RT, whereas SEI possesses a 3 nm amorphous layer as core‐shell of Li_2_S at 60 °C (**Figure** **15**a,b).^[^ ^364^ ^]^ For RT, the rate‐controlling step is Li diffusion with highly‐crystalline Li_2_S. The reaction rate gradually decreases with time, ultimately constructing a stable passivation layer. But, at 60 °C, with accelerated diffusion of Li and reaction kinetics, SEs undergo severe decomposition in the disordered Li‐P‐S‐Cl structure with reduction Li and numerous nuclei, which reveals the polycrystalline phases. Such order‐disordered Li_2_S phase transitions show parasitic reactions for high temperatures (reaction‐controlled kinetics). Further, the amorphous layer enlarges with time, increasing interphase thickness with diffusion‐controlled kinetics and high interface resistance (Figure 15c,d). PDADMATFSI:LiTFSI (1:1.5) has high conductivity compared to PDADMATFSI and without LiTFSI, illustrating that polyIL facilitates Li‐ion conduction. LATP and LAGP NASICON type SEs combined with various artificial SEI such as Li_3_PO_4_, LiF, MgF_2_, B_2_O_3_, PEO, Al_2_O_3_, AZO, ZnO, LiPON, IL‐LAGP, and SiO_2_ resolves the reduction of SEs by Li. Promisingly ZnO exhibits prolonged cycle life for Li/Li cells of 2000 h.^[^ ^365^, ^366^, ^367^ ^]^ Shi et al.^[^ ^368^ ^]^ presented PVDF‐BaTiO_3_‐Li_0.33_La_0.56_TiO_3–x_ CSEs (PVBL), in which polarized dielectric BaTiO_3_ improves the Li‐salt dissociation with forming numerous mobile Li^+^ that transfer across the interface with Li_0.33_La_0.56_TiO_3–x_. Besides, BaTiO_3_‐Li_0.33_La_0.56_TiO_3–x_ confines the SCL formation with PVDF, and these CSEs homogenize the interface electric field. The Li/PVL (without BaTiO_3_) has a lower overpotential relative to PVBL that ascribes the partial reduction of LLTO from Ti^4+^ to Ti^3+^ for PVL by Li with the formation of a mixed conductor interface.^[^ ^369^, ^370^ ^]^

The Li/poly(lithium 4‐styrenesulfonate)(PLSS)‐Li_6.5_La_3_Zr_1.5_Ta_0.5_O_12_ (LLZTO) CSEs cells show stronger coordination that built the bridge for Li^+^ transfer, lower energy barrier, and higher diffusion coefficient. It introduces two different interfaces such as LLZTO/PLSS and PLSS/Li; the former is constructed by coordination chemistry of Li^+^ migration due to the coordination of ‐SO_3_Li with LLZTO surface atoms. The latter interface stems from the superior lithiophilicity of PLSS, and it prevents electrons from attacking electrolytes due to the feature of electron blocking.^[^ ^371^ ^]^ Li with triphenyl antimony (p‐TPA)@LLZTO enables the mixed ionic/electronic interface with Li_3_Sb, Li_2_C_2_, and LiSbO_3_ domains, improving the mass and charge transport for Li and LLZTO. The phenyl groups in the a‐TPA interlayer imply an even distribution and restrained growth of lithiophilic Sb^0^ sites that favor the homogenization of electric‐field distribution, Li alloying, and nucleation processes.^[^ ^372^, ^373^ ^]^ Li_5.4+x_P_1−x_Si_x_S_4.4_Cl_1.6_ (x = 0, 0.05, 0.1, 0.2, 0.3, 0.4) argyrodite explains the Si doping, in which the LiCl‐dominated interphase layer as buffer formed with a homogeneous distribution of Si, P, S, and Cl in the grains. Si doping undergoes P sites with forming metastable argyrodite phases. A large ionic radius of Si^4+^ than P^5+^ exhibits tetrahedral coordination, whereas Cl^−^ has a smaller ionic radius than S^2−^.^[^ ^135^ ^]^ The Si enlarges the volume of (Si,P)S_4_ polyhedrons; therefore, the 4a/4d sites (Wyckoff positions) for argyrodites occupy the preferential S that results extrusion of Cl atoms from the lattice. Si‐doped Cl‐rich argyrodites suppress Li dendritic depositions even for high current densities.

Typically, LGPS have a 1D major transport pathway along the c‐axis in bulk LGPS. Evaluation of the total Li‐ion diffusion pathway from bulk to grain boundaries (GB) is shown in Figure 15e. Two energy barriers are involved for Li‐ion conduction for LGPS. Path 1 shows Li‐ion transfer from bulk‐like (BL) to GB through the grain‐boundaries interface (GBI), whereas path 2 involves Li‐ion diffusion across GB.^[^ ^374^ ^]^ For Li‐ion migration, path 1 and 2 has energy barriers of 0.248 and 0.286 eV, respectively, which is higher than calculated energy barriers for bulk and comparable to experimental values. The improved energy barrier for Li‐ion near GB is relative to complex inhomogeneous atomic structures at the GB, which results in Li‐ion exits from BL to the c‐axis for 1D pathways to cross the intersection facing numerous further Li ions exits from the BL. The diffusion for GB is the key factor in determining the overall diffusion properties for LGPS during operations. Significant reduction in diffusion rates for Li‐ions at GB influences severe parasitic reactions owing to sluggish kinetics; thus, structural contact interfaces are critical for improving the electrochemical performances of SEs. Interface resistances are severely influenced by volume change with stress/strain that declines the physical and chemical contact. A comprehensive understanding of SEs/electrode interfaces is necessary to alleviate the interface stress/strain. Previous reports propose that the rate capability of cathodes is strongly subjective to lattice stain due to Li‐ion conductance and minimal blocking defects.

These findings suggest interfacial impedance strongly influences the interface compatibility of Li and rate capabilities. Besides, it provides information for the fabrication of superior interface characteristics for prolonged operational life with Li‐metal for the rising attention of all‐solid‐state Li‐metal batteries (ASSLMBs). The inorganic LiF and Li_3_N phases based SEI is more favorable for stable interfacial reactions than other inorganic/organic phases, which can withstand high stress/strain and strong electric field. Poor chemical compatibility, volume change, and low ion conductivity of SPEs exhibit a passive layer with high resistance during cycling, even for extremely poor current densities with accelerating chemical diffusion kinetics. SIEs with sulfides, oxides, and composites display more compatible interface properties with wide electrochemical windows. The commercial realization for Li‐metal anodes will not be assured because no present results meet the manufacturing criteria relative to electrochemistry and scalable processing.

#### 7.1.2 Li‐Sulfur (Li‐S) Batteries

**Figure 16:**
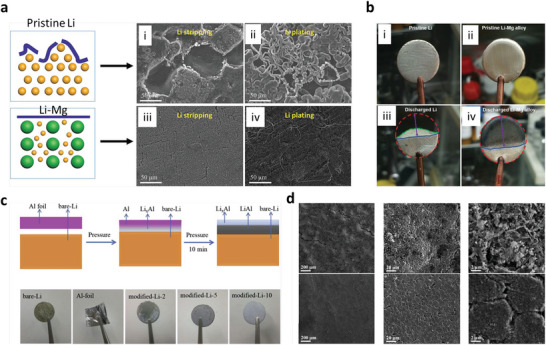
a) SEM images of Li and Li–Mg alloy after i, iii) Li stripping and ii, iv) following Li plating. b) Photographs of i, iii) Li foil, and ii, iv) Li–Mg alloy foil before and after Li stripping. Reproduced with permission.^[^ ^393^ ^]^ Copyright 2019, Wiley VCH. c) Illustration of Li‐B anode modified by in situ reaction with aluminum foil (top), and typical photograph of bare‐Li, aluminum foil, modified‐Li‐anodes (bottom). Reproduced with permission.^[^ ^392^ ^]^ Copyright 2018, Elsevier. d) SEM images for pristine Li (top row) and Li/C/Sn (bottom row) electrodes after 250 cycles. Reproduced with permission.^[^ ^391^ ^]^ Copyright 2019, Elsevier.

Li‐S chemistry has been acknowledged overwhelming consideration due to the high cathode and anode capacities of ≈1675 and ≈3800 mAg^−1^, respectively, which yields a maximum energy density of 2600 Wh kg^−1^ with active sulfur and Li for 2–2.5 V operating voltage.^[^ ^375^, ^376^, ^377^ ^]^ However, the polysulfide shuttle and cell failure by Li‐dendritic growth are critical challenges for Li–S batteries that rigorously decreases the feasibility for active S‐electrodes, lowers cycle life, and capacity fading. Li–S batteries with SEs can resolve polysulfide diffusion and block dendrite growth. Two main phases for SEs in Li–S have been considered for research. Integration of SEs for Li–S obtained cell performance which is disappointing with cycle life, S‐cathode utilization, and rate‐capability due to poor kinetics for S‐cathodes and electrode/electrolyte interfaces. Liquid electrolytes for cathodes provide ion media for sulfur–polysulfide–sulfide redox reactions and sustain simple Li‐ion pathways for cathode or SEI. Several approaches, such as artificial buffer layers at Li/SEI, mechanical pressing of Li in SEs, liquid‐soaked polymer addition among the Li and SEs, and hybrid electrolytes, have been reported; however, they could not provide satisfactory solutions.^[^ ^375^, ^376^, ^377^, ^378^ ^]^ NASICON‐type Li_1+x_Al_x_Ti_2−x_(PO_4_)_3_‐based Li–S batteries demonstrate both the chemical and electrochemical compatibilities influencing the Li‐S chemistries by reduction of Ti^4+^ to Ti^3+^ for 2.4 V versus Li/Li^+^ by polysulfide species.^[^ ^379^, ^380^ ^]^ Further, replacing different SEs such as Li_1+x_Al_x_Ge_2−x_(PO_4_)_3_, garnet, LGPS, Li_2_S‐P_2_S_5_‐P_2_O_5_, Li_x_La_2/3‐x/3_TiO_3_, Li_1+x_Y_x_Zr_2−x_(PO_4_)_3_, LiPON, Li_7_La_3_Zr_2_O_12_–PEO–LiClO_4_, Li_6_PS_5_Cl, PEO–LiTFSI, PEO–LiCF_3_SO_3_, and PEO–LiClO_4_, and LLZO SEs could improve the compatibility concerns. In contrast, operational cycle life and capacity fading are not reasonable.^[^ ^379^, ^380^, ^381^, ^382^, ^383^, ^384^, ^385^, ^386^, ^387^, ^388^, ^389^ ^]^ To improve Li interface structures with SEs, different Li‐alloys (**Figure** **16**) have been reported, such as Li–In, Li–Sn, Li–Al, Li–Mg, etc.^[^ ^390^, ^391^, ^392^, ^393^ ^]^

Li/LGPS with LiI displays prolonged cycle life for 800 h; however, pristine Li/LGPS exhibits an increased overpotential of 2 V with cell failure.^[^ ^394^ ^]^ EIS displays a severe increase in interface resistance from 72 to 6000 Ω, illustrating the electrochemical decomposition of LGPS and physical deterioration with cracks and large Li volume change. XPS shows reduced P, Li_3_P, and PS_4_ for LGPS; however, with LiI, Li/LGPS did not show a reduction of Ge^4+^ or P^5+^, which indicates an excellent interface with dendrites. Incorporating LiI enhances the mechanical strength, chemical stability, and excellent toughness that offers suitable chemistry with sulfide electrolytes.

**Figure 17:**
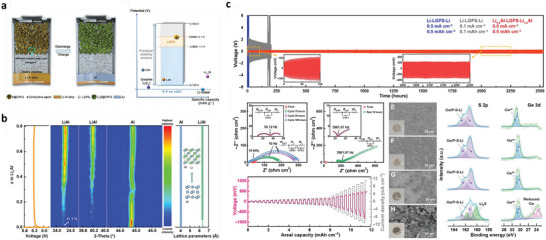
a) Schematic of a rechargeable all‐solid‐state LSB with Li‐Al alloy anode and its reaction mechanism. b) In situ XRD patterns for lithiation processes for Al and their lattice parameters of Al and LiAl. c) Compatibility tests for Li_0.8_Al anodes under LGPS SSEs. Reproduced with permission.^[^ ^395^ ^]^ Copyright 2022, American Association for the Advancement of Science.

**Figure** **17** proposes the carbon‐ and binder‐free Li–Al alloy anode and their compatibility with LGPS. LGPS has an EW of 1.7–2.1 V versus Li/Li^+^, Li‐In alloy, or Li_4_Ti_5_O_12_ of 1.55 V versus Li/Li^+^ with an operable potential of <1.7 V (Figure 17a).^[^ ^395^, ^396^ ^]^ Oxidative limit of LGPS is 2.8 V versus Li/Li^+^. During lithiation, the LiAl peaks increase gradually with the Al peak. It indicates Li_0.8_Al is a mixture of Al and LiAl with Al:LiAl = 1:4 with biphasic reactions at contestant potential. Lattice parameters of Al (Fm3m) and LiAl (Fd3m) verify the volume change of Li_0.8_Al (74.95%), which is lower than those of other Li_4.4_Si and Li_4.4_Ge alloy anodes (Figure 17b).^[^ ^397^ ^]^ The (111) crystal plane of LiAl initiates to appear with the lithiation step. Li_0.8_Al/LGPS/Li_0.8_Al cells exhibit a low overpotential of 100–150 mV over 2500 h, whereas Li/LGPS/Li exceeds the detecting limits with overpotential >3–6 V. Alloy concentrations for Al, Si, Sn, In, and Mg significantly affect the cycle stability. Li_0.8_Al has mixed ionic‐electronic conduction kinetics and displays Li‐ion conducting SEI. Li_0.8_Al/LGPS has smooth morphology without dendrites; however, Li/LGPS has rough and uneven growth. XPS suggests LGPS strongly reduced by Li forming Li_2_S and reduction of Ge^4+^ (Figure 17c).

**Figure 18:**
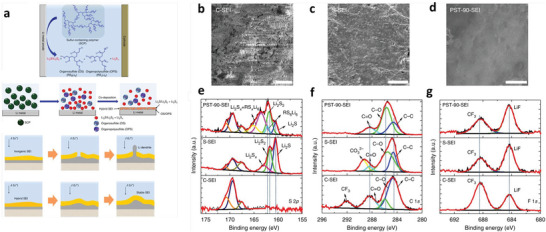
a) Schematics illustration. b‐d) SEM image of C‐SEI layer (b), S‐SEI layer (c), and PST‐90‐SEI layer (d). e‐g) XPS spectra of S 2p (e), C 1s (f), and F 1s (g) for the SEI layer. Reproduced under the terms of a Creative Commons CC BY 4.0 license.^[^ ^398^ ^]^ Copyright 2017, Nature Publishing Group.

PST was reported by copolymerization of sulfur and vinylic monomer triallylamine with forming inorganic/organic hybrid SEI by organosulfides/organopolysulfides for Li‐metal surface (**Figure** **18**).^[^ ^398^ ^]^ Organosulfide/organopolysulfide acts as a plasticizer that creates more flexible and stable Li depositions without Li dendrites. SEM images display porous and loose structure for C‐SEI, smooth with few cracks for S‐SEI, and uniform and planar morphology without cracks for PST‐90‐SEI, which illustrates C‐SEI has continuous breaks, S‐SEI cannot withstand volume change, and PST‐90‐SEI suppress dendrites with enhanced CEs. XPS spectra verify the presence of Li_2_S_x_ and Li_2_S_2_ in S‐SEI corresponds to the Li polysulfide phases. PST‐90‐SEI confirms the organosulfides (RS_x_Li_6_) and Li_2_S_x_ phases, and C‐SEI shows –CF_3_ functional groups. F 1s spectra display the presence of more substantial LiF peaks for PST‐90‐SEI than those of C‐SEI and S‐SEI, enabling a stable SEI. Several Li‐alloy anodes have been reported because they effectively reduce the Li nucleation overpotential and interfacial resistance by controlling Li electrodepositions (**Table** **3**).^[^ ^390^, ^391^, ^392^, ^393^, ^394^, ^395^, ^396^, ^397^, ^398^, ^399^, ^400^, ^401^, ^402^, ^403^, ^404^ ^]^ Binary Li‐alloys have several critical issues, such as great volume change (i.e., Li‐Si, Li‐Sn), high cost (i.e., Li‐Ag, Li‐Cu), high reaction activity (i.e., Li‐Na), and low energy density (i.e., Li‐Bi, Li‐Zn), thus ternary/multiple Li‐alloys have typical advantages, but specific energy is reduced because additional metals did not involve for electrochemistry. Substantial alteration for specific volume during charge/discharge leads to a loss in electrical contacts with capacity fading. Further, complex fabrication processes and prices are also critical to be considered. Li‐alloy with graphene, CNTs, polymers, and anodic aluminum oxide (AAO) membrane effectively limits the volume change during electrochemistry.^[^ ^405^, ^406^ ^]^ Sun et al.^[^ ^407^ ^]^ reported LAGP)/PVDF composite layer over the Li metal prevents the polysulfides by Li reactions. Li‐S battery shows great cycle stability for LiNO_3_‐free electrolytes with a discharge capacity of 832.1 mAh g^−1^ after 100 cycles at 0.5C.

**Table 3:** State‐of‐the‐art for Li‐alloys‐based anodes.

Metal alloys	Methods	Remarks
Li‐Sn	Fusion reaction, Electrochemical lithiation	Small interface impedance, a strong affinity for Li, poor reversibility
Li‐Si		Large volume expansion, poor reversibility, high overpotentials
Li‐Ge	Electrochemical lithiation	Faster Li diffusion, poor reversibility
Li‐B	Fusion reaction	Porous structures accommodate Li, and structure collapse
Li‐Al	Sputtering, Fusion reaction, Electrochemical lithiation	Small volume change, phase transfer,
Li‐Bi	Electrochemical lithiation	High volumetric capacity, working temperature >380 °C
Li‐In		High electro‐positivity of Li, high cost
Li‐Sb		Working temperature >350 °C, large volume expansion
Li‐Mg		High Li diffusion, poor Li kinetics
Li‐Na		Large volume expansion, high reactivity
Li‐Au		Less structure change, high cost, eliminating the nucleation barriers
Li‐Ag	Fusion reaction, Electrochemical lithiation	
Li_x_CuP	Fusion reaction	Good cycling stability
Li_4.4_Ge_x_Si_1‐x_	Ball milling	Increase Li Ion accommodation, good reversibility
Li‐Cu‐Sb	Ball milling, Electrochemical lithiation	Good cycling stability and electronic conductivity
Li_2_MgSi	Ball milling + annealing	Prevent dissociation of Li‐Mg, complex synthesis
Li_x_Cu_6_Sn_5_	Electrochemical lithiation	Small irreversible capacity
Li‐B‐Mg	Fusion reaction	Complex synthesis, porous structure, good strength
Li‐In‐Sb	Ball milling + Electrochemical lithiation	Good reversibility, small volume change

The comprehensive analysis confirms that Li‐S chemistry has several technical limitations to reaching commercial standards like LIBs or other batteries. Furthermore, Li or Li‐alloys anodes have higher reduction potentials to polysulfide shuttle effect and dendrites growth; thus, Li metal protection with Li‐S chemistry is more complex than commercial LIBs. Modifying the chemical properties of in situ or ex‐situ SEI with regulating current distributions for Li depositions is the future scope for research. Artificial buffer layers for anodes make uniform Li‐ion flux and balanced mechanical yield; hybrid layers offer advantages of organic and inorganic components that can form a stable interface.

#### 7.1.3 Sodium (Na)–Metal Batteries (SMBs)

Na metal has many interests due to its natural abundance, low cost, high theoretical capacity of 1166 mAh g^−1^, low redox potential of −2.71 V versus SHE, and equivalent physicochemical properties with Li.^[^ ^408^, ^409^ ^]^ However, the high reactivity of Na endorses the inevitable side reactions and dendritic growth, which illustrates the severe deterioration of electrochemical performances with unstable SEI, consumption of electrolytes, and serious safety or thermal concerns limiting their practical applications. Much effort has been considered to overcome these intrinsic shortcomings with four major approaches 1) optimization for electrolytes in terms of solvents, additives, and salts for liquid or gel electrolytes as well as finding electrolytes that are compatible for high voltage cathodes using ionic liquids or high‐concentration electrolytes; 2) constructing artificial SEI layer with a superior interface by Na anode; 3) develop SEs with high ion conductivity and exceptional interface compatibility that functions physical barriers to block parasitic reactions and mechanical barriers to suppress Na‐dendrites; 4) constructing 3D confined frameworks with high sodiophilicity.^[^ ^408^, ^409^, ^410^, ^411^, ^412^ ^]^ Such a 3D framework modifies the nucleation and growth kinetics by providing a porous host that can accommodate Na‐anode volume change and controls the Na^+^ nucleation process for stable electrodeposition. The high surface of 3D frameworks regulates local current density and Na^+^ flux distribution with inhibition of mossy Na‐dendrites. Na‐infused carbon felt, wood, oxygenated CNTs, Fe_2_O_3_‐coated carbon textiles, and Sn^2+^ pillared Ti_3_C_2_ MXene are the most effective frameworks reported.^[^ ^408^, ^413^, ^414^, ^415^, ^416^ ^]^ Thus, it is critically necessary to develop solid‐state Na‐batteries. Similar to Li‐batteries SEs, the SEs for Na‐batteries are also devolved in terms of SPEs, oxide, halides, NASICON, β‐Al_2_O_3_, sulfides, complex hydrides, etc.^[^ ^413^ ^]^ A widespread understanding of nucleation behavior and initial stages of Na‐growth to obtain stable metallic electrodepositions is essential. Electrodeposition and thermal infusion are basic approaches for storing Na‐metal; however, electrodeposition is highly vulnerable to uneven nucleation and growth, having high localized current and overpotentials. In the case of thermal infusion, poor wettability among the molten Na and host causes intermittent Na distributions.

**Figure 19:**
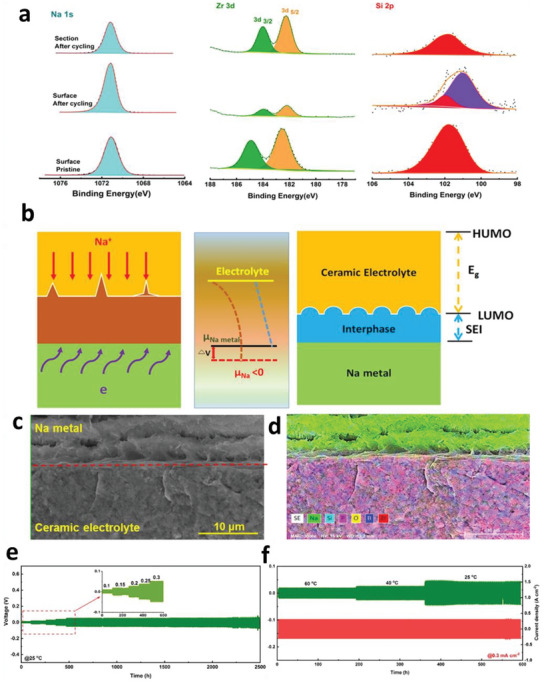
a) Na 1s, Zr 3d, and Si 2p XPS spectra for pristine, surface, and section after cycling with NZSP‐10NBO. b) Illustrations for potential distribution among the Na metal and SSEs for various interphases, Na/NZSP (left) and Na/NZSP‐10NBO (right). c,d) Cross‐SEM and EDS map for NZSP‐10NBO//Na interface after 2500 h. e,f) Na plating/stripping for NZSP‐10NBO at RT (e) and different temperatures (f). Reproduced with permission.^[^ ^417^ ^]^ Copyright 2021, Elsevier.

The grain‐boundaries engineering approach for stabilization of the Na//Na_3_Zr_2_Si_2_PO_12_ (NASICON) interface and Na‐ion transport across the interface has been reported (**Figure** **19**).^[^ ^417^ ^]^ XPS analysis verifies the stronger intensity for Na 1s ascribes to enhanced coverage of interface products. Zn 3d and Si 2p for NASICON electrolytes suggest a reduction to sub‐oxides (Figure 19a). Theoretical calculations also justify the reduction in Zn^4+^ and Si^4+^ for NASICON by Na‐metal.^[^ ^418^ ^]^ Zhang et al. verified the Zr 3d, Na 1s, Si 2p, P 2p, and O 1s spectra for the pristine NASICON and after Na‐depositions, in which they found an increase of Na‐metal fractions after two‐deposition steps without interphase. Zr and Si undergo reductions to ZrO_x_ or Si_x_O_y_ from varying +4 to +3 and +2 to +1 oxidation states, respectively. P 2p did not show any noticeable reduction in NASICON. Figure 19b shows the desired low resistance stable SEI interphase for Na//NZSP‐10NBO//Na cells. NASICON‐NBO shows the formation of Na^+^‐rich SEI with a reaction of Na‐blocking electron transport that guarantees stable Na‐migration. Cycled Na‐anode did not show obvious dendrites with stable and uniform Na‐electrodepositions (Figure 19c,d). High ion conductivity (1.72 mS cm^−1^) and better compatibility with Na‐metal exhibits homogeneous Na‐plating/stripping cycles of 2500 h for Na//Na_3_Zr_2_Si_2_PO_12_‐10wt% Na_2_B_4_O_7_//Na cells for RT and elevated temperatures (Figure 19e,f). Wang et al.^[^ ^419^ ^]^ reported the discrete Na metal islands for NASICON at 1250 °C with Na dendrites of 10 mm, whereas NASICON (at 1300 °C‐15 min/1200 °C) shows the uniform distribution, suggesting Na‐enriched SEI by using TOF‐SIMS/depth‐profile maps. Insertion of plasticizer in SEs accelerates the Na^+^ transfer kinetics in the cathodes.^[^ ^419^ ^]^

**Figure 20:**
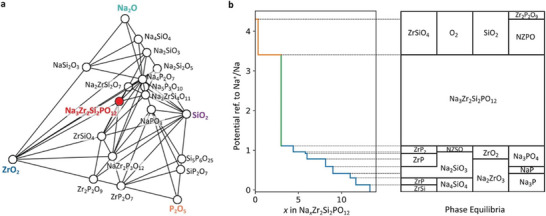
a) Pseudoquaternary phase diagram for Na_2_O–ZrO_2_–SiO_2_–P_2_O_5_ system. b) Voltage profile of Na_3_Zr_2_Si_2_PO_12_ and the phase equilibria of all the reaction stages. The calculated EW for Na_3_Zr_2_Si_2_PO_12_ is from 1.11–3.41 V versus Na^+^/Na). [NaZr_2_(PO_4_)_3_ – NZPO, Na_2_ZrSi_2_O_7_ –NZSO]. Reproduced with permission.^[^ ^418^ ^]^ Copyright 2020, American Chemical Society.

**Figure** **20** shows the pseudo‐quaternary phase diagram of Na_3_Zr_2_Si_2_PO_12_ using open quantum materials database (OQMD). The ground‐state structure of Na_3_Zr_2_Si_2_PO_12_ drops on the energy convex hull, illustrating the stable phase formation. Na_3_Zr_2_Si_2_PO_12_ has EW of 1.11–3.41 V versus Na/Na^+^. Phase equilibria and decomposition reaction energies of Na_3_Zr_2_Si_2_PO_12_ reveal reduction initiates from 1.11 V, demonstrating instability against Na. Reduction for Na_3_Zr_2_Si_2_PO_12_ at 0 V forms Na_3_P, Na_2_ZrO_3_, Na_4_SiO_4_, and ZrSi with small reaction energy of −0.27 eV/atom. The LGPS, Li_7_P_3_S_11_, and LiPON decomposition energies at thermodynamic equilibria with Li are −1.25, −1.67, and −0.66 eV/atom, respectively.^[^ ^420^ ^]^ This feature confirms the small thermodynamic driving force and slower kinetics for reduction reaction than those of thiophosphates and LiPON owing to the lowest decomposition energy (−0.27 eV/atom) of Na_3_Zr_2_Si_2_PO_12_ in equilibria. This reaction energy involves the parabolic rate constants for diffusion‐controlled SEI growth.^[^ ^421^ ^]^

**Figure 21:**
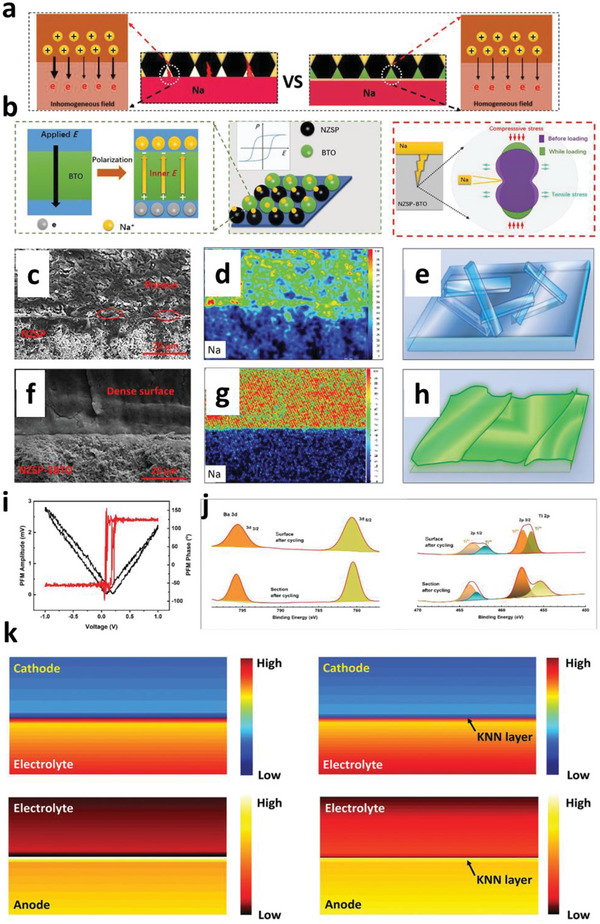
a) Na metal dendrite suppression kinetics for different SIEs. b) Ferroelectricity influence for Na^+^ distribution. c,f) SEM images and d,g) EDS maps for Na anode after cycling with Na/NZSP/Na and Na/NZSP‐3BTO/Na cells. e,h) Schematic illustrations for surface analysis. i) Local PFM hysteresis loops of NZSP‐3BTO after cycling. j) After cycling, Ba 3d and Ti 2p XPS spectra of the surface and section of NZSP‐3BTO/Li cells. Reproduced with permission.^[^ ^422^ ^]^ Copyright 2022, Wiley VCH. k) Simulated Na‐ion concentration at cathode/SEs and anode/SEs interfaces with/without the K_0.5_Na_0.5_NbO_3_ layer. Reproduced under the terms of a Creative Commons CC BY 4.0 license.^[^ ^424^ ^]^ Copyright 2022, Wiley VCH.

Brissot et al. propose the classical model for the progression of ions transfer and concentrations through the Li plating process and their impact on dendritic nucleation and growth. When ion depletion occurs for inhomogeneous SCL among the metal electrodes and SIEs, the dendrites initiate nucleation and aggressively grow upright to metal surfaces (Li, Na, K, **Figure** **21**a).^[^ ^269^, ^422^ ^]^ Unbalanced ions and electron transports with an inhomogeneous electric field for Na//SIEs interface are two major kinetics features. Removal of these features plays a significant role in realizing thermodynamically favored Na‐electrodepositions. The ferroelectric phase stems from spontaneous polarizations to create the internal electric field and macroscopic charges for ferroelectric surfaces. External polar species are absorbed for the ferroelectric surface to screen surface charges.^[^ ^423^ ^]^ Producing different electric fields to realize homogenous distributions of space charge locally conflicting with the original field among the metal and SIEs, defined as the ferroelectric effect, would be a feasible approach. The ferroelectric phase in ceramics lattice shows the bridging or deflection for the cracks and devours the driving force of cracks propagation Figure 21b. This mechanical energy transferred to electrical energy by piezoelectric effect or concurrently disbursed by the stress‐persuaded ferroelectric phase transformations results in the improvement in the fracture toughness for ceramic electrolytes. Incorporation of ferroelectric phases such as (BaTiO_3_, K_0.5_Na_0.5_NbO_3,_ and others) to NASICON SIEs and A_3‐2x_Ba_x_ClO (A = Li, Na, K, x = 0.005–0.01) accelerates the Na^+^(others)‐migrations and uniform distributions of charges for the Na//NASICON or Na//A_3‐2x_Ba_x_ClO interfaces during cycling illustrating the dense Na‐/other‐metals electrodepositions with high CCD.^[^ ^422^, ^424^, ^425^ ^]^

Na//NASICON‐3BTO shows flat and dense morphology, whereas wattle‐like Na‐metal for NASICON only (Figure 21c–h), verifying the superior interface that ascribes to the even Na‐depositions persuaded by dynamically self‐adaptive interfaces. Ferroelectric phase BTO provides the conformal and switchable electric polarization for ordered distributions of charge carriers. Meanwhile, an additional out‐of‐plane piezoelectric field initiated from plating/striping‐persuaded stress lifts the Na^+^‐transports. Local hysteresis loop displays the typical polarization switching even after cycle operations corresponding to a high PFM value and a 150^o^ shift for phase signals (Figure 21i). XPS shows notable changes for Ti^4+^ and Si^4+^ states, which indicates the existence of Ti and Si in lower valence for Na/NASICON‐3BTO interface (Figure 21j). Figure 21k displays theoretical results for Na‐ion distributions for electrode/electrolyte interfaces with/without ferroelectrics.^[^ ^424^ ^]^ For the cathode/electrolyte interface, Na^+^‐accumulated and Na^+^‐deficient layers are separately formed for interfacial cathodes and electrolytes. To keep equilibria, Na^+^‐moderately appears closer to Na^+^‐accumulated layers inside the provisional electrolytes. Similar types of ion distributions are obtained for the anode/electrolyte interface. Preferred ferroelectric polarizations efficiently diminish the SCLs for electrolyte/ferroelectric/electrode interfaces. The substitution of Sc^3+^, Yb^3+^, Zn^2+^, Mg^2+^, Al^3+^, Nd^3+^, Y^3+^, Ge^4+^, Ga^3+^, and Nb^5+^ in the NASICON has also been researched to enhance the ion conductivity (maximum *σ_Na_* ^+^ ≈2.44 mS cm^−1^) and compatibility with Na‐anodes.^[^ ^426^ ^]^ Chi et al.^[^ ^427^ ^]^ reported the stable Na//Sn/beta‐alumina SEs/Sn//Na interface with 54 Ω cm^−2^ area‐surface‐resistance (ASR) for 1000 h. Insertion of Sn interlayer for Na//beta‐alumina interface shows the vivid lowest values of interfacial and charge‐transfer resistances are 9.6  and 26.7 Ω cm^−2^, respectively, which indicates the modified interfaces are comparable to those of garnet‐type SEs interfaces.^[^ ^428^ ^]^

**Figure 22:**
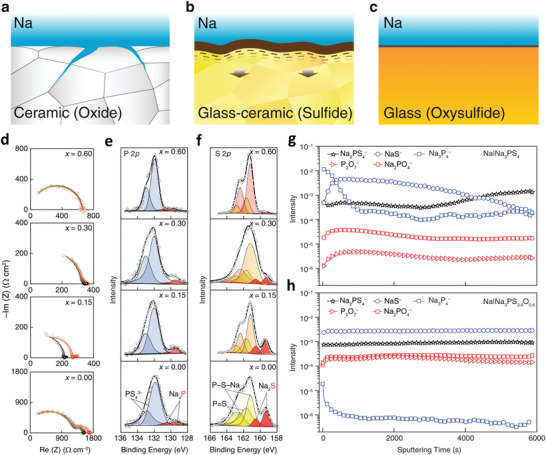
a) Na dendrites propagate along grain boundaries of oxide electrolytes, b) decomposition of sulfide‐based glass‐ceramic electrolyte contacting Na metal, c) homogeneous oxysulfide glassy electrolytes form a stable interface with Na metal. d) EIS spectra for Na//SEs//Na as prepared (black) and after 5 h (red). e,f) XPS spectra for P 2*p* (e) and S 2*p* (f) for Na/SEs interface. g,h) TOF‐SIMS depth profiles for Na//Na_3_PS_4_, and Na//Na_3_PS_3.4_O_0.6_ interfaces. Reproduced under the terms of a Creative Commons CC BY 4.0 license.^[^ ^432^ ^]^ Copyright 2022, Nature Publishing Group.

Ceramic (β‐Al_2_O_3_ and NASICON‐type oxides) SIEs display superior chemical stability for Na‐metals; however, high ion‐conductivity can be obtained when they are treated to nearby theoretical densities, which requires >1500 °C annealing temperature for long‐time‐interval and possess poor wettability with Na‐metal owing to rough and rigid surfaces.^[^ ^429^ ^]^ Previous reports confirm the Na‐metal prefers the propagation with distinct grain boundaries for SEs generating dendritic growth with eventual cell failures (**Figure** **22**a).^[^ ^430^ ^]^ This is a source of controversy for SEs because ceramic/oxide SEs have an excess modulus of 200 GPa and offer beyond adequate elastic and shear modulus to repel Na‐dendrites. Glass‐ceramic SEs (Na_3_PS_4_ or others) possess submissively soft surfaces with fewer precise grain boundaries owing to substantial amounts of glassy phases (5–50 vol%) that can fade the dendritic growth. However, interfacing with Na exhibits the unstable SEI (Figure 22b),^[^ ^431^ ^]^ thus alloy type anodes (Na‐Sn, Na‐Au, or other) are preferred that can increase the voltage of anodes but degrades the energy density. Until now, no SEs can show all the requirements for high‐energy SSBs regarding chemical, electrochemical, mechanical, and process parameters. Thus, researchers have reported mixed oxysulfide (Na_3_PS_3_O, Na_3_PS_4−x_O_x_; NSPO) based SEs//Na metal interfaces relative to pristine sulfide SEs.^[^ ^432^, ^433^ ^]^ This consists of fine grains, agglomerated powders, and oxygen‐content‐dependent structures illustrating dense and homogeneous smooth glasses even for nominal 300 MPa. Formation of homogenous bulk glassy phase is a critical character to stabilize Na‐metal in terms of mechanical, chemical, or electrochemical features (Figure 22c).

EIS (Na//NPSO//Na) displays mixed bulk and grain‐boundaries resistance with capacitance (high‐frequency); characteristic capacitance obtained from capacitance and interface resistance for SEI of Na/SEs (mid‐frequency, Figure 22d). Figure 22e,f displays two doublet pairs of P 2p and S 2p spectra for Na//Na_3_PS_4−x_O_x_, x  =  0, 0.15, and 0.30 SEs interfaces, which ascribes to the reduced sulfide and phosphide species. Theoretical and experimental results manifest that the reduced species of Na_2_S and Na_3_P have mixed ionic and electronic conducting behavior that cause decomposition of SEs with unstable SEI, well consistent to pristine Na_3_PS_4_, Na_3_SbS_4_, Na_3_PSe_4_ SEs.^[^ ^434^, ^435^ ^]^ ToF‐SIMS (Figure 22g,h) displays two strong signals of NaS^−^ and Na_3_P_4_^−^ fragments for Na//Na_3_PS_4_ interface. It is reduction of Na_3_PS_4_ to Na_2_S and Na_3_P as: 8Na + Na_3_PS_4_ → 4Na_2_S + Na_3_P. SEI thickness is ≈1000s of Cs^+^ etching. Whereas, Na_3_P_4_^−^ fragments intensity is three orders (10^−5^) lower in magnitude for Na//Na_3_PS_3.4_O_0.6_ interface, which verifies the initial reduction of SEI suppresses for a factor of 1000 and SEI thickness reduced by a factor of 10 and later interface stabilizes with Na‐metal plating. Self‐passivating nature ascribes to the formation of insulating Na_2_O. Other fragments P_2_O_7_^−^, Na_2_PO_4_^−^, and Na_2_PS_4_^−^ are from the bulk Na_3_PS_3.4_O_0.6_.

Weng et al.^[^ ^436^ ^]^ reported interface stabilities for Na//Na_3_SbS_4_//Na, Na//Na_2.95_Sb_0.95_W_0.05_S_4_//Na, and Na/Na_2.95_Sb_0.95_W_0.05_S_3.9_O_0.1_/Na cells, in which Na/Na_2.95_Sb_0.95_W_0.05_S_3.9_O_0.1_/Na interface suppresses the resistance due to the W and O co‐doping. Oxygen doping for Na_3_SbS_4_ via W and O co‐doping alleviates the degradation of Na/SEI induced by W substitution. Interface formation kinetics among the Na_3_BS_4_//Na and Na_3_PS_4_//Na systems display a stationary Na plating/stripping with few nanometers interphase passivation.^[^ ^437^ ^]^ The orthorhombic NaAlCl_4_ SEs (*σ_Na_^+^* ≈0.0039 mS cm^−1^) demonstrates the 1D‐preferred 2D Na^+^‐conduction pathways and voltage stability ≈4 V versus Na/Na^+^. NaCrO_2_‐Na_3_Sn//NaAlCl_4_//Na displays 82.9% capacity retention after 500 cycles at 1C.^[^ ^156^ ^]^ The Na_3_Sb//Na_3_PS_4_|Na_3_Sb alloy‐based anode cells display stable and lowest overpotentials of 150 mV over 500 h relative to those of Na_15_Sn_4_//Na_3_PS_4_//Na_15_Sn_4_ (1.8 V), which ascribes to the high electrochemical potential of Na_3_Sb/Na_3_PS_4_ SEs (≈0.4 V versus Na/Na^+^) and lower interface resistance.^[^ ^438^ ^]^ The composite of Na_3_SbS_4_ (NSS, 30 wt%) with oxysulfide glass (NaPSO) demonstrates significantly strengthened interface stability with a polarization potential of 0.42 V after 200 h compared to those of NaPSO and NSS (>4 V versus Na/Na^+^), which verifies the effective isolation of NSS from Na‐metal enhancing the interface reactions.

Wang et al.^[^ ^439^ ^]^ reports the interfacial kinetics for perfluoropolyether‐terminated polyethylene oxide (PEO)‐based block copolymer (EO10‐PFPE, *t_Na_^+^* of 0.46 and *σ_Na_^+^* of 0.047 mS cm^−1^) SPEs/Na for safe and stable SMBs. Block copolymer tolerates self‐assembled *bcc* nanostructures having high storage modulus for 100 °C, and PEO domains provide transport channels for high‐salt concentrations (ethylene oxide/sodium  =  8/2). Insertion of PFPE‐species in PEO significantly impedes voids and dendrites formation implying stable SEI for Na‐metal surface. This structure is highly advantageous for diminishing side reactions and restricting SEI growth, suggesting a stable plate/strip.^[^ ^440^, ^441^ ^]^ Cell resistance for Na/Na cells with EO10‐CTRL/Solupor exhibits continuous shifts to lower values with abrupt drops implying cell failures. Whereas with EO10‐PFPE/Solupor displays ultra‐stable cell resistance upon cycle operations for 1000 h, which verifies the formation of stable SEI among the EO10‐PFPE and Na‐metals.

**Figure 23:**
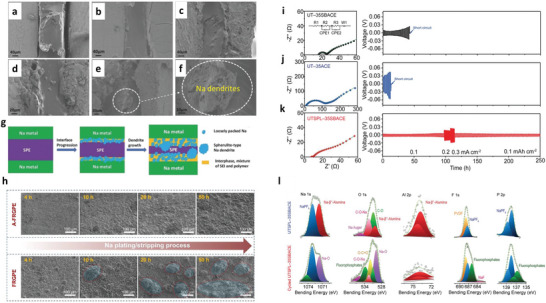
Cross SEM images for Na//SPEs//Na cells for interface kinetics: a) as‐fabricated, b) after heating at 80 °C for 12 h, c) after 10 h cycle operations and d) after 100 h cycle operations, and e) cycled to short circuit and f) enlarged dendrite SEM. g) Schematics of the development and migration for Na//SPEs interfaces. Reproduced with permission.^[^ ^446^ ^]^ Copyright 2018, Wiley VCH. h) Ex‐situ SEM images for Na anodes with Na/A‐FRGPE/Na and Na/FRGPE/Na cells. Reproduced with permission.^[^ ^443^ ^]^ Copyright 2023, Elsevier. Impedance and symmetric cycles of i) Na/UT‐35SBACE/Na, j) Na/UT‐35ACE/Na, and k) Na/UTSPL‐35SBACE/Na cells. l) XPS data of the UTSPL‐35SBACE membrane before and after cycling in a symmetric Na/Na cell. Reproduced with permission.^[^ ^444^ ^]^ Copyright 2023, Wiley VCH.

The SMBs failure kinetics compared to LMBs are still not well investigated. Wei et al. explain that Na‐metal's electrolyte depletion during operations is critical for SMBs failure besides Na‐dendrites growth based short‐circuit.^[^ ^442^ ^]^ This feature was confirmed by observing voltage diversion rather than abrupt drops related to SMBs failure, and it ascribes to the relative softness of Na‐metal (RT hardness ≈0.5 MPa and shear modulus ≈3.3 GPa) compared to Li‐metal. SEM images for as‐fabricated, thermally treated, and failed cells and after 10 or 100 h cycling without abrupt voltage drops (**Figure** **23**a–g) were analyzed to demonstrate failure kinetics. Larger gaps are obtained among the SPEs (thickness ≈160 µm) and Na‐electrodes, which ascribes to the communal influence of fewer superlative conformal loading for SPE on Na and the experimental splitting processes. Thermal treatment (80 °C for 12 h) successfully removes these gaps. After 10 h operations, the Na//SPEs interface exhibits an irregular surface with small voids nearby the interface. Notably, after 100 h operations, SPEs thickness was severely reduced from 160 to 60 µm. Further, it decreases to ≈30 µm after short‐circuits with larger Na‐dendrites on the SPEs surface. Specifically, there is no sign of Na dendrites for the early stages of cycle operations (<100 h cycling). Such substantial reduction for SPEs is well relative to the reported SMBs.^[^ ^442^ ^]^ Na metal//asymmetric flame‐retardant GPE (A‐FRGPE) have homogeneous dense topography relative to those of FRGPE (rough and dendrites with uncontrolled Na^+^ flux, Figure 23h). Insertion of g‐C_3_N_4_ (pyridinic‐N) in FRGPE delivers numerous lone‐pairs to seize metal‐ions,

promotes Na‐salt dissociation, and decreases Na^+^ nucleation overpotential, and molecular structure with porous channels suggest the enhanced Na^+^‐transport.^[^ ^443^ ^]^ EIS of Na//Na cells for UT−35SBACE, UT−35SBACE, and UTSPL‐35SBACE ultrathin CSEs displays total interface resistances of 66.8, 200.2, and 43.1 Ω, respectively (Figure 23i–k).^[^ ^444^, ^445^, ^446^ ^]^ UTSPL‐35SBACE//Na interface exhibits lower polarization potential with stable cycling relative to others. Na 1s, O 1s, and Al 2p show peaks for 1071.8, 530.2, and 74 eV, corresponding to sodium‐beta‐alumina, which illustrates the stability of SBA particles with PVDF (Figure 23l).^[^ ^446^ ^]^ Extensive efforts have been performed to develop different types of polymer SPEs and hybrids with ceramics or sulfides such as biopolymers or celluloses or PEO‐ or PVDF‐NaTFSI or NaClO_4_, PEO‐NaClO_4_‐TiO_2_, PEO‐NSS, PEO‐NPSO, or PEO‐NASICON and Na‐alloys anodes such as (Na‐Mg, Na‐Sn, Na‐Sb, Na‐Au, Na‐Ag, etc.) to stabilize the Na‐metal/SPEs or CSEs interfaces to obtain overall high performances for SMBs or SIBs.^[^ ^439^, ^440^, ^441^, ^442^, ^443^, ^444^, ^445^, ^446^, ^447^, ^448^, ^449^ ^]^ However, poor ion conductivity, oxidation/reduction potential, and limited thermal/electrochemical stabilities inhibit their practical applications.

The comprehensive analysis clarifies that the primary goal for next‐generation SMBs or SIBs is obtaining long‐time operations with retaining high capacity or energy density and safety performances at affordable cost. Poor ion conductivity of SEs and deprived wettability for Na‐metals demonstrate unsatisfactory performances relative to those of liquid counterparts. Considerable interfacial resistance for Na//SEs interfaces limits the homogeneous Na‐electrodepositions and deteriorates chemical instability with high voltage hysteresis. Solid/solid interface or inside SEs have several physical/chemical contacts among the Na//SEs with multiple grain boundaries or defects, which results in uncontrolled dendritic growth. Thus, the controlled Na‐nucleation with uniform depositions for multiple solid interphases is crucial for all solid‐state SMBs future.

#### 7.1.4 Potassium‐Ion Batteries (KIBs)

Potassium batteries offer prospective alternatives for LIBs or SIBs for grid‐scale energy storages owing to the low cost, earth abundance, and low K/K^+^ electrode potential (−2.93 V versus SHE, comparable to LIBs −3.04 V versus SHE) relative to those of Na, which implies K‐batteries has a higher voltage, power or energy densities.^[^ ^450^, ^451^ ^]^ However, two fundamental challenges, such as poor K//SEs interfacial contacts and limited areal capacity caused by the intrinsic low melting point or poor mechanical strength of K, dendrites growth, and small K self‐diffusion coefficient impedes their practical applications.^[^ ^283^ ^]^ The low affinity of K with SEs provides the random distribution of micropores for interfaces. Thus charge flux localizes anomalously upon application of current at the interface with significant voltage polarization, dendritic growth, and lower areal capacity. Previous efforts have been carried out to improve K metal wettability by inserting sodiophilic or lithiophilic interlayers or composites with reduced surface energy for low interface resistance and conformal interface.^[^ ^301^, ^452^ ^]^ However, the delamination of the K//SEs interface occurs for repetitive deposition/dissolution to the initial conformal interface with unsatisfactory CCD and areal capacities, which ascribes to sluggish self‐diffusion kinetics of K.^[^ ^452^ ^]^ Metal atoms at the interface convert to metal ions under applied current and transfer in the SEs with the formation of interface vacancies. This illustrates the growth of larger micropores due to poor self‐diffusion coefficients of metals that cannot fill the vacancies from transferred metal ions. Several approaches have been reported for limiting interface delamination, such as increased pressure or temperature, composite SEs, 3D‐interconnected interfaces, etc. Carbonaceous materials such as carbon allotropes, graphene, polymers, or CNTs show the feasibility of improving K‐diffusion kinetics and sustainability of de‐/intercalation chemistries.^[^ ^452^, ^453^, ^454^ ^]^

**Figure 24:**
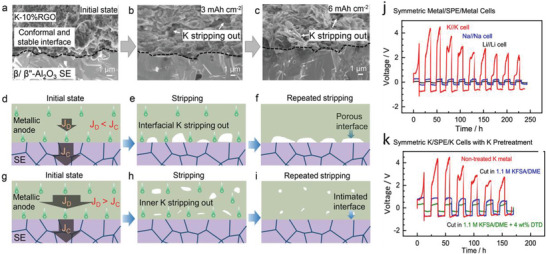
SEM images of K–10% RGO/SEs interface for a) initial states, b) K strips for 30 h, and c) for 60 h. Schematics of interfacial structure evolution after strips under two different conditions, d–f) *J*_D_ < *J*_C_ and g–i) *J*_D_ > *J*_C_. Reproduced with permission.^[^ ^450^ ^]^ Copyright 2023, Wiley VCH. Voltage profiles of symmetric (j) Li/SPE/Li, Na/SPE/Na, and K/SPE/K cells (non‐treated) and (k) K/SPE/K cells with pretreated K metal. Reproduced under the terms of a Creative Commons CC BY 4.0 license.^[^ ^195^ ^]^ Copyright 2022, American Chemical Society.

Wu et al.^[^ ^450^ ^]^ reports the 3D interconnected and conductive network for K‐10% reduced graphene oxide (RGO) composite ultrathin metal anodes (50 µm) with favorable K^+^‐diffusion coefficient (2.38 × 10^−8^ cm^−3^ s^−1^) toward high‐temperature stability (200 °C), which illustrates the fast/versatile K‐migration pathways, alleviates the leakage of fused K, and suppress structure deformations enabling homogeneous distribution and thermo‐stability of K. K–10% RGO symmetric cells with β/β′‐Al_2_O_3_ SEs confirms the stable K‐plate/stripping with lower interface resistance of 1.3 Ω cm^2^, high areal capacity (11.86 mAh cm^−2^), and CCD (2.8 mA cm^−2^). **Figure** **24**a–c exhibits conformal K–10% RGO//SE interface for the initial state without micropores; even for 3 mAh cm^−2^ K‐stripping interface is well intact, and for 6 mAh cm^−2^ interface is still well retained with the formation of several micropores. Interface inhomogeneity manifests uneven current distribution due to voids at the interface upon cycling and self‐diffusion kinetics that are unable to replenish transported K‐ions from interphase to SEs.^[^ ^455^ ^]^ Like Li//SEs,^[^ ^456^ ^]^ J_D_ defines the K^+^‐flux based on K self‐diffusion in metal foil and J_C_ the K^+^‐flux in SEs due to applied current density. For J_D_<J_C_, metal ions from nearby surfaces distribute faster relative to the bulk with the formation of voids and strong delamination of metal anodes from SEs after continuous cycling, which illustrates cell failure for lower current densities (Figure 24d–f). For J_D_>J_C_, the metal ions firmly migrate from the bulk by replenishing the adjacent ions that can disperse in SEs. This results in the simultaneous preservation of intact interfaces and voids creation for the bulk of anodes (Figure 24g–i). K‐metal provides high energy relative to other reported anodes (carbon, Sn‐Sb, metal‐carbon, oxides, MoSSe). Monoclinic‐KFeHCF displays flat potential plateaus for galvanostatic K^+^ extraction/insertion through two‐phase reactions. In contrast, cubic‐KFeHCF has tangential curves via a single‐phase regime verifying superior potential stabilities for K.^[^ ^457^ ^]^

Theoretical calculations verify crystal structures, cation‐anion interactions, and defects influence the interface transport for borohydride SEs//K.^[^ ^458^ ^]^ Neutral NH_3_B_3_H_7_ includes the H^+^ (in NH_3_) and B_3_H_8_^−^ anions with hydritic H that can combine across dihydrogen bonding. The B_3_H_8_^−^·NH_3_B_3_H_7_ complex has more positive electrostatic potentials (−4.48 to −1.55 eV) relative to the B_3_H_8_^−^ anions (−5.37 to −4.05 eV), which illustrates weak interactions for K^+^ than B_3_H_8_^−^.^[^ ^459^ ^]^ Thus coordination of NH_3_B_3_H_7_ exhibits reduced binding interactions among the K^+^ and B_3_H_8_^−^ facilitating superior mobility for K^+^ ions (*t_K_^+^* ≈0.93). EWs for KB_3_H_8_·NH_3_B_3_H_7_, KB_3_H_8_·0.5NH_3_B_3_H_7_, and KB_3_H_8_·1.5NH_3_B_3_H_7_ are of 1.2–3.5, 1.4–3.3, and 0.9–3.2 V, respectively. For increasing EWs, incorporating oxides such as SiO_2_ or Al_2_O_3_ is the promising approach owing to strong interfacial reactions among the KB_3_H_8_·NH_3_B_3_H_7_ and oxides. Notably, long operations for K‐ion SEs are very scarce due to the high reactivity of K‐metals. Zheng et al.^[^ ^460^ ^]^ presented antiperovskite K_3_OI and K_2.9_Ba_0.05_OI (*σ_K_^+^* ≈3.5 mS cm^−1^) SEs for interface compatibility analysis. For K_3_OI, K‐ions migrate through K‐vacancies and larger vacancies nearby the disordered I–O sites, whereas K_2.9_Ba_0.05_OI greatly reduces *E_a_* of 0.36 eV with K‐ion migration from K vacancies and the possible anion disorder activated for higher temperatures. Compared to LGPS, LPS, NPS, and LLZO for Li or Na, for K_3_OI, O, and I have oxidation states of −2 and −1 that cannot reduce further by K‐metal or K‐alloy anodes.^[^ ^461^ ^]^ K//K_2.9_Ba_0.05_OI//K symmetric cell shows 50 mV overpotential for 0.5 mA cm^−2^. Further, K_3_Sb_4_O_10_(BO_3_) SEs show displacement of K^+^ via 1D interconnected channels with good interface stability (low *E_a_* and interface resistance).^[^ ^462^ ^]^ The K_2_Fe_4_O_7_ SEs with 3D open frameworks for K^+^‐ion transfer containing FeO_6_ octahedral (oct) and FeO_4_ tetrahedral (tet) sites along vertices and edges enables rapid transport for K‐ions with the stable interface over EW of 5 V versus K/K^+^.^[^ ^463^ ^]^ Besides, K_2_M_2_TeO_6_ (M = Ni, Mg) and KC_8_//KNH_2_‐based SIEs were also reported for K^+^‐ion transport stabilities.^[^ ^464^, ^465^ ^]^

Li//SPE and Na//SPE cells exhibit 50 and 280 mV polarization for respective depositions/dissolutions, whereas K//SPE cells display unstable voltage polarizations with larger polarization >1 V, which illustrates larger polarizations are from higher K//SPEs interfacial resistance (Figure 24j).^[^ ^195^ ^]^ Interface resistances for Li, Na, and K are 10^3^, 10^4^, and 10^5^ Ω cm^2^, respectively, which verifies the degree of polarization.^[^ ^466^ ^]^ Further, reduced plate/strip polarizations ascribe the FSA^−^ decomposition and K//K cells high polarization even after 200 h decreases reliability.^[^ ^467^, ^468^ ^]^ The optimal molar ratio for [EO]/[K^+^] is 10 for PEO‐KFSI SPEs with *σ_K_^+^* ≈0.27 mS cm^−1^ for 60 °C.^[^ ^467^ ^]^ Pre‐treatment for K‐metal exhibits reduced interfacial resistance with efficient passivation of the surface by reductive decomposition of FSA^−^ ions, which obtains polarization of 300 mV with stable K‐metals (Figure 24k). PPC‐KFSI‐cellulose SPEs confirm reduction and oxidation peaks of −0.3 and 0.21 V versus K/K^+^ with ≈100% CEs suggests SPEs interface stability with K‐metal.^[^ ^469^ ^]^ The (PEO)_30_/KBPh_4_ SPEs with Prussian blue electrodes show reversible K^+^‐ion de‐/intercalation with a reversible 20 mAh g–1 capacity and lower‐voltage hysteresis.^[^ ^470^ ^]^ Rayung et al.^[^ ^471^ ^]^ reported polyurethane acrylate‐based SPE due to inspiration of coordination of characteristic of N─H, C═O, and C‐O‐C groups for K^+^ stabilization; however, it limits EW ≈ 2 V. PEO‐based SPEs with K‐salts (such as KCl, KFSI, KBr, CH_3_COOK), polyester‐based ([‐O‐ (C═O) –O‐]) and polyurethane (─[NH─(C═O)─O]n─) SPEs and others (PVP, PVA) have been reported for K^+^ interface kinetics; however, their poor ion‐conductivity (10^−5^–10^−8^ S cm^−1^) and operating temperatures, and EWs inhibits the chemical and electrochemical stabilities against K‐metals.

Zhang et al.^[^ ^472^ ^]^ reported poly(vinylidene fluoride‐hexafluoropropylene) potassium bis(fluorosulfonyl)imide polyacrylonitrile (PVDF‐HFP‐KFSI@PAN) GPEs (*σ_K_* ^+^  ≈0.36 mS cm^−1^) for K//GPEs interface with stable SEI and K^+^‐plating/stripping over 1200 h. It displays a maximum plating capacity of 300 mAh cm^−2^ for K/K^+^ cells relative to those of previous reports (K‐rGO@3D‐Cu, K‐ACM, and Sn@3D‐K of 125, 116.5, 50 mAh cm^−2^ in liquid electrolytes). Further, the cardanol‐based GPEs with 0.67–0.78 MPa modulus show *σ_K_* ^+^  ≈0.36 mS cm^−1^ and EW of −0.2–5 V versus K/K^+^.^[^ ^473^ ^]^ Overall, solid‐state KIBs are in the initial stages due to several limitations from SEs and K‐metal anodes regarding ion‐conductivity, EWs, and K‐metal compatibilities. Comprehensive understanding vindicates the rational design of advanced SEs with stable SEI, stable for high voltage cathodes, and a wide operating temperature range for KIBs will be the future research approach. Further stabilization of electrode/electrolyte interface for continuous K^+^‐plate/strip using artificial SEI or carbon‐based, metal‐alloys, or oxides‐based anodes is also necessary to consider as a major focus.

### 7.2 Multivalent‐Ions Battery Technologies

#### 7.2.1 Magnesium‐Ion Batteries (MIBs)

Multivalent ion batteries (Mg, Zn, Al, Ca) offer great interest due to high energy densities by multi‐electron reactions relative to those of monovalent Li‐, Na‐, and K‐ion batteries.^[^ ^474^, ^475^, ^476^, ^477^ ^]^ Among these, magnesium (Mg) batteries offer promising candidates for large‐scale energy storage owing to their intrinsic merits: 1) high volumetric capacity of 3830 mAh cm^−3^ (versus 2060 mAh cm^−3^ for Li), 2) insensitive dendrites formation behavior and low air‐sensitivity compared to those of Li, Na or K metals, 3) lower electrode potential (−2.37 V versus SHE) relative to those of Zn and Al, 4) high mechanical yields and low chemical reactivity, 5) light metal (density ≈1.74 g cm^−3^), abundant resources, and low cost.^[^ ^478^, ^479^ ^]^ However, MIBs are far from their commercial prospects due to several fundamental issues, such as sluggish solid‐state diffusion kinetics of Mg^2+^ ions, poor working voltage, and poor cycle life.^[^ ^480^, ^481^ ^]^ Formation of a non‐conductive passivation layer over Mg anode with polar aprotic solvents and magnesium salts (magnesium perchlorate, magnesium tetrafluoroborate, TFSI, imide, carbonate, and nitrile), poor EWs, CCs instability, and polysulfides formation prevents migration of Mg^2+^ ions during plate/strip processes.^[^ ^480^, ^481^ ^]^ Thus, obtaining reversible Mg^2+^‐depositions/strips with compatible electrolyte/electrode interfaces is critically essential. SEs or artificial protection layer with fast Mg^2+^ mobility is favorable for alleviating these challenges.^[^ ^482^ ^]^

**Figure 25:**
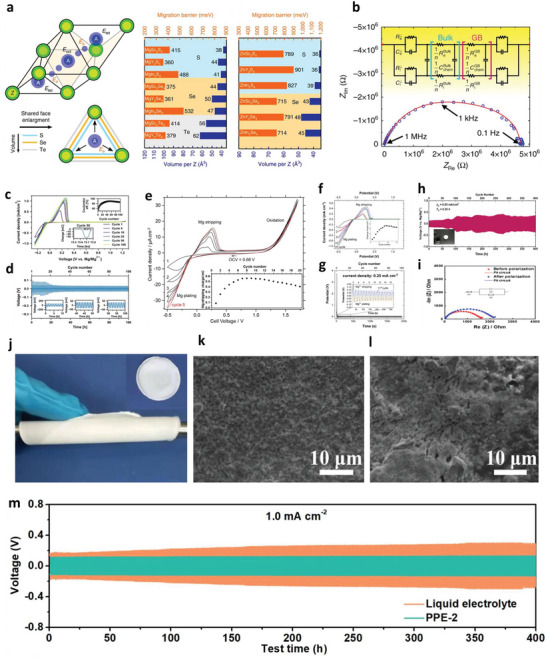
a) Theoretical calculations for Mg and Zn migration barriers in sulfides, selenides, and telluride's AX_2_Z_4_ spinel's (with A  =  Mg or Zn). b) EIS for the Ta/MgSc_2_Se_4_/Ta cell. Reproduced under the terms of a Creative Commons CC BY 4.0 license.^[^ ^143^ ^]^ Copyright 2017, Nature Publishing Group. c) CV and d) Plate/stripping cycles for Mg//Mg(BH_4_)_2_.1.5THF‐MgO(75wt%)//Mg cells at 55 °C. Reproduced with permission.^[^ ^490^ ^]^ Copyright 2022, Wiley VCH. e) CVs for t/Mg(en)_1_(BH_4_)_2_/Mg cell at 60  °C. Reproduced under the terms of a Creative Commons CC BY license.^[^ ^493^ ^]^ Copyright 2017, Nature Publishing Group. f) CV for Au|1.6NH_3_@MgO|Mg. g) Symmetric cell for Mg|1.6NH_3_@MgO|Mg system. Reproduced with permission.^[^ ^76^ ^]^ Copyright 2020, American Chemical Society. h) Symmetric cycles for Mg//CPS//Mg cells, inset CPE photo. i) EIS for Mg//CPS/Mg cells before/after polarizations. Reproduced with permission.^[^ ^498^ ^]^ Copyright 2019, American Chemical Society. j) PVDF‐HFP SPEs. k,l) SEM for Mg anodes with PEE (k) and liquid (l). m) Mg//SPEs//Mg cell cycling. Reproduced with permission.^[^ ^501^ ^]^ Copyright 2021, Elsevier.

Extensive efforts have been explored for Mg‐ion mobility in solids owing to their poor transport kinetics *σ_Mg_^2+^*, and sluggish diffusion kinetics.^[^ ^482^ ^]^ Canepa et al.^[^ ^143^ ^]^ reported the magnesium scandium selenide spinel (MgSc_2_Se_4_) SIEs with *σ_Mg_^2+^* of 0.01–0.1 mS cm^−1^. Spinel structures show Mg atoms undergo tet‐sites instead of favorable oct‐sites with reducing *E_a_* for Mg^2+^‐ions diffusion. Theoretical calculations explain migration barriers trend for different anions as O^2−^ > S^2−^ > Se^2−^ > Te^2^.^[^ ^483^ ^]^ Lower the migration barriers – higher the volume per anion; a higher volume of anions retain large electric polarizability with limiting cation mobility. In spinel structures of MgX_2_Z_4_ (Z = S, Se and X = In, Y, Sc), ion transport among two tet‐sites ensues by vacant oct‐sites shared with tet‐sites illustrates the migration topology tet–oct–tet. Migration barriers are evaluated by migration‐ion energy shared to triangular (tri) surfaces of oct‐ and tet‐sites subjective to dimensions and anions of tri‐surfaces.^[^ ^484^ ^]^ Several chalcogenides such as MgSc_2_S_4_, MgIn_2_S_4_, MgSc_2_Se_4_, MgY_2_Se_4_, ZnSc_2_S_4_, ZnY_2_S_4_, ZnIn_2_S_4_, ZnY_2_Se_4_, MgY_2_S_4_, MgIn_2_Se_4_, ZnSc_2_Se_4_, ZnIn_2_Se_4_, MgSc_2_Te_4_, and MgY_2_Te_4_ have been considered for investigation, in which MgY_2_S_4_ (≈360 meV), MgY_2_Se_4_ (≈361 meV), and MgSc_2_Se_4_ (≈375 meV) has superior conduction kinetics. Migration barriers of 361–375 meV (MgY_2_Se_4_ and MgSc_2_Se_4_) suggest greater Mg mobility equivalent to Li‐conductors such as LISICON‐like (≈ 200–500 meV) and Garnets (≈400–500 meV).^[^ ^483^, ^484^, ^485^, ^486^ ^]^ Zn migration barriers are larger for S/Se spinels (>700 meV) than those of Mg analogs, clarifying the less favorable coordination (**Figure** **25**a). EIS (Figure 25b) displays high mobility of Mg^2+^ for Ta//MgSc_2_Se_4_//Ta cells with mixed ion‐conduction behavior. EIS explains coupling of Jamnik–Maier circuit elements in series possesses the bulk and grain boundary contributions. Wang et al. reported 5 and 10 wt% Se‐rich MgSc_2_Se_4_ SEs to possess similar *σ_Mg_^2+^* with MgSc_2_Se_4_ SEs, whereas electronic conductivity is severely increased.^[^ ^141^ ^]^ Further, substituting Sc^3+^ with Ti^4+^ or Ce^4+^ confirms the decrease in electronic conductivity and increase in *σ_Mg_^2+^* that is favorable for reversible Mg^2+^‐plate/stripping.^[^ ^143^ ^]^ Koettgen et al.^[^ ^487^ ^]^ reported Mg^2+^ mobility and stability kinetics with MgLn_2_X_4_ (Ln = Lu, Tm, Er, Ho, Dy, Tb, Sm, Pm, Nd, Pr, La, and X = S, Se). The Mg^2+^ migration barriers were reduced with linear behavior for lanthanides except for MgLa_2_S_4,_ which ascribes the Mg destabilization for oct‐states.

Energy profiles display localized, stable sites for the spinel structure of Mg is tet‐sites in all materials. The tri has the smallest Mg–X distance and highest energy (transitions) states concerning oct and tet local minima. It suggests preferable excess tet‐ or small oct‐environments for these Mg^2+^‐chalcogenides. Typically, stable materials have E_hull_ = 0, and fabricated sulfides and selenides are metastable due to E_hull_ = 50 meV per atom, which ascribes low surface energy and preferential nucleation.^[^ ^488^ ^]^ MgLu_2_S_4_, MgLu_2_Se_4_, MgTm_2_Se_4_, MgEr_2_Se_4_ and MgTm_2_S_4_, MgEr_2_S_4_, MgHo_2_Se_4_ shows E_hull_ < 25 and < 50 meV per atom with lowest energy structures, respectively. Spinel structures confirm the vacant oct‐sites among the two tet‐sites is the Mg^2+^‐migration pathway. Imanaka et al.^[^ ^489^ ^]^ reported Mg^2+^‐conduction behavior for MgZr_4_P_6_O_24_, Zr_2_O(PO_4_)_2_, and Mg_1+x_Zr_4_P_6_O_24+x_ + xZr_2_O(PO_4_)_2_ composites (x = 0.4) for 800 °C, in which composite achieves 2.3 times higher *σ_Mg_^2+^* than Mg_1.15_Zr_4_P_5.7_Si_0.3_O_24_.

Since Mohtadi et al.^[^ ^153^ ^]^ displayed full inorganic and halide‐free SIEs enabling Mg reversible plating/stripping, Mg(BH_4_)_2_‐based several electrolytes such as Mg(BH_4_)(NH_2_), Mg(BH_4_)_2_(en), Mg(BH_4_)_2_(NH_3_), Mg(BH_4_)(BH_3_NH_3_)_2_, Mg(BH_4_)_2_.xNH_3_, and Mg(BH_4_)_2_.xNH_3_‐MgO have been investigated. The monoclinic composite [Mg(BH_4_)_2_.1.5THF‐MgO(75wt%)] SEs with σ_Mg_^2+^ of 10^−4^ S cm^−1^ for 70 °C, *t_Mg_^2+^* of 0.99, and EWs of ≈1.2 versus Mg/Mg^2+^ have been demonstrated.^[^ ^490^ ^]^ The analogs of amine magnesium borohydride composites are also reported for improving the *σ_Mg_^2+^* as Mg(BH_4_)_2_⋅xNH_3_ with MgO nanoparticles. The larger surface area of MgO confines the molten state and prevents crystallization with retaining *σ_Mg_^2+^*.^[^ ^76^, ^491^ ^]^ CV (Figure 25c) shows the initiation of anodic current movement at ≈1.2 V versus Mg/Mg^2+^, which illustrates oxidation SEs. It is less apparent for increased cycles signifying the stable interface formation for reversible Mg^2+^ plating/stripping. Figure 25d displays the electrochemical compatibility of Mg(BH_4_)_2_.1.5THF‐MgO (75 wt%) SEs with Mg with steady polarizations. Mg(BH_4_)_2_‐based SIEs consist of Mg‐ions at tet cages surrounded by four BH_4_^−^ anions ascribes the lower conductivity, whereas, for high temperatures, tet Mg^2+^ coordinates two BH_4_^−^ and two NH_2_^−^ anions increase the *σ_Mg_^2+^* and Mg//SIEs compatibility.^[^ ^492^ ^]^ Magnesium ethylenediamine borohydride (Mg(en)_1_(BH_4_)_2_) SIEs with coordination of Mg^2+^‐ions and neutral bidentate ethylenediamine ligands in the ratio of one bidentate ligand per metal atoms show *σ_Mg_^2+^*  ≈0.05 µS cm^−1^ at 30 °C that stems transport of Mg^2+^ ions. CV displays −0.2 V versus Mg/Mg^2+^ for Mg plating and 0.5 V versus Mg/Mg^2+^ for reverse stripping, inducing Mg‐interface capability (Figure 25e).^[^ ^493^ ^]^ Higashi et al.^[^ ^149^ ^]^ also verified the oxidative potential of Mg(BH_4_)(NH_2_) SEs is 3 V versus Mg/Mg^2+^. Further, Mg–S, Mg–FeS, and Mg–Ag_2_S‐based cells with Mg(BH_4_)(NH_2_) SEs show OCV of 1.4, 1.2, and 1.3 V at 150 °C, respectively. Theoretical calculations confirm the 2D Mg‐diffusion channels are perpendicular to the c‐axis. The Mg‐atoms transfer to the interstitial sites by forming Frenkel pairs for relatively smaller energies is the Mg‐migration precursor states.

CVs of Au//1.6NH_3_@MgO//Mg asymmetric cell display an increase in plate/strip current for the initial 10 cycles implying interface contact for electrode/SEs enhances upon initial cycles without electrolyte decompositions from 0.5–1.2 V. Upon 20 cycles, potential range increases for −0.5 to 2.5 V with irreversible oxidation for >1.2 V, which indicates stable interface layer that conducts Mg^2+^ ions with enhancing electrochemical stability (Figure 25f,g). Golub et al.^[^ ^494^ ^]^ stated diffraction of Mg(en)_2_(BH_4_)_2_ is achievable for the ratio of Mg(en)_3_(BH_4_)_2_ and Mg(BH_4_)_2_ (2:3) by using cryomilling. The formed Mg(en)_2_(BH_4_)_2_ phase is thermodynamically favorable, while Mg(en)1.2(BH_4_)_2_ is a meta‐stable intermediate. NASICON (Mg_0.5_Zr_2_(PO_4_)_3_) is also fabricated by sol‐gel method with *σ_Mg_^2+^*  ≈1 and 71 µS cm^−1^ for RT and 500 °C. Mg_0.5_Zr_2_(PO_4_)_3_ SEs have EWs of 2.50 V versus Mg/Mg^2+^ and a transfer number of 0.69.^[^ ^495^ ^]^ Magnesium bis(oxalate) borate‐based SEs also reported.^[^ ^496^ ^]^ Zheng et al.^[^ ^497^ ^]^ reported MG3@MOF‐199 and MG3@MOF‐5 based SEs as Mg‐ion conductors with *σ_Mg_^2+^* of 1.93 and 0.056 µS cm^−1^ with *E_a_* of 0.234 and 0.568 eV, respectively. Ion conduction is strongly influenced by the mole fraction of Mg‐salts and the pore size of frameworks.

Deivanayagam et al.^[^ ^498^ ^]^ reported CPEs with mixture of PVDF‐HFP, Mg(ClO_4_)_2_, 1‐butyl‐1‐methylpyrrolidinium bis(trifluoromethyl)sulfonyl imide, and TiO_2_ nanoparticles. It offers 0.16 mS cm^−1^ ion‐conductivity and stable Mg//Mg cycle operations for 400 h with lower overpotentials of 0.1–0.3 V comparable to liquid counterparts with stable electrolyte/electrode interfacial resistance (Figure 25h,i). Significant coordination for Mg^2+^ and TFSI^−^ with amorphization using TiO_2_ fillers describes the superior performance. Further, to enhance the *σ_Mg_^2+^* and mechanical properties of Mg‐SPEs, inserting inorganic fillers such as MgO, TiO, SiO_2_, Al_2_O_3_, and ZnO is a highly successful strategy.^[^ ^499^, ^500^ ^]^ Wang et al.^[^ ^501^ ^]^ reported PPE‐based SEs with *σ_Mg_^2+^* of 0.47 mS cm^−1^ and EW of 3.1 V (Figure 25j–m). Further, Mg//Mg plate/strip displays 0.13 V overpotential for 1700 cycles with uniform Mg^2+^ loading over the anode. Reversible Mg plating/stripping is observed for various SPEs or GPEs such as PEO/Mg(BH_4_)_2_, PMMA‐MgTr, oligo(ethylene oxide)‐grafted polymethacrylate‐Mg‐salt, P(VdF‐co‐HFP), Mg(ClO_4_)_2_‐SiO_2_, and Mg(AlCl_2_EtBu)_2_‐PVdF, however, poor ion transfer kinetics and interfacial instabilities constrains the MIBs performances.^[^ ^102^, ^298^, ^502^, ^503^, ^504^, ^505^, ^506^, ^507^ ^]^

Mg metal undergoes massive volume expansions of 300–500% with strong stress‐strain upon solid‐solid phase transformations; the Mg‐alloy type anode can be the alternate solution. Several Mg‐alloy‐based anodes, such as β‐Mg_3_‐Bi_2_, Mg_2_‐Ga_5_, Mg_2_Sn, Mg_2_‐Sb, and MgF_2_‐Mg, are also reported to increase the compatibility of Mg‐alloys anodes.^[^ ^508^, ^509^, ^510^, ^511^ ^]^ Su et al.^[^ ^229^ ^]^ proposes the uniform and conformal magnesium phosphorus oxynitride (MgPON) thin films over Si substrates (thickness ≈62 nm) by using ALD. MgPON SEs *σ_Mg_^2+^* is of 62 nS cm^−1^, 0.36 µS cm^−1^, and 1.2 µS cm^−1^ at 400, 450, and 500  °C, respectively. Despite extensive research stating the feasibility of MIBs, no anode and electrolyte materials are compatible and resolve the significant primary challenges of the passivation effect over Mg anodes, EWs, and ion‐conductors for SEs. The MIBs future pathways are a proper cathode‐electrolyte configuration with Mg anodes offering high capacity and voltage cells. Stronger electrostatic forces among the Mg and surrounding anions causes diffusion kinetics severely. For electrolytes, primary materials should be compatible with Mg anodes and cathodes by electrochemically, non‐corrosive liquids with reasonable safety and low cost. Artificial buffer layers such as carbon‐based, heavy metals (Au, Ag, Ta, La, Ce, W), or oxides over Mg anode can be suitable approaches to minimize the Mg passivation.

#### 7.2.2 Zinc‐Based Batteries (ZBs)

Zinc metal has been considered a promising anode due to its high gravimetric and volumetric capacities (820 mAh g^−1^ and 5855 mAh cm^−3^), small standard potential (−0.762 versus SHE), low cost and toxicity, abundant resources, environmental benignity, and intrinsic safety.^[^ ^512^, ^513^ ^]^ Zn chemistries insistently undergo irreversibility, poor CEs, dendritic growth, chemical instabilities for low/high current densities, low Zn utilization, and insufficient areal capacity.^[^ ^514^, ^515^ ^]^ Higher charge‐discharge rates are typically utilized to minimize the influence of irreversibility for cycle operation; however, it undergoes severe decomposition of electrolytes. Further, significant excess Zn is required to retain the supply due to consumption by parasitic reactions; however, it displays considerable underutilization of its theoretic capacities.^[^ ^516^, ^517^ ^]^

The construction of all‐solid‐state ZBs is a promising approach for these offensive concerns because of their high chemical stability. Thus far, inorganic Zn^2+^‐conducting SEs have been rarely reported owing to the stronger electrostatic binding among the Zn^2+^ ions and solid lattice that outcomes poor *σ_Zn_^2+^* (<10^−5^ S cm^−1^) and *t_Zn_^2+^*.^[^ ^518^ ^]^ Usually, previous reports comprise the addition of free water or liquid plasticizer (15–70 wt%; PEO, PVDF, poly(vinylidene fluoride‐co‐hexafluoropropylene); PVHF) blended with ILs or electrolyte‐soaked membranes; however, it illustrates hydrogen evolution or corrosion reactions.^[^ ^519^ ^]^ SIEs with high *σ_Zn_^2+^*, large EWs, and long‐life compatibility similar to SIEs for LIBs are highly challenging owing to the multivalent behavior of Zn^2+^.^[^ ^520^ ^]^ SPEs display considerable benefits of ion‐conductance over inorganic materials with high flexibility and interface computability with metal anodes.^[^ ^521^ ^]^ Inorganic fillers are critically essential to enhance ion conductance for SPEs. This approach enables the dissociation of Zn salts and ion‐transport channels over filler surfaces and functions as plasticizers for decreasing polymer crystallinity and improving segment migration.^[^ ^522^ ^]^ Thus fillers enriched surface chemistries with a high surface area are greatly anticipated for SPEs.

Theoretical calculations explain the doping of F^−^ with S^2−^ introduces numerous vacancies of Zn (Vac_Zn_) for mesoporous ZnS (MFZS) with reducing Zn^2+^ migration barriers for nearby Zn oct‐sites for Zn_y_S_1‐x_F_x_ crystal phase as super Zn^2+^‐ion conductors.^[^ ^518^ ^]^ Zn‐MFZS displays EW of −0.5–3 V, *E_a_* ≈0.3 eV, and *σ_Zn_^2+^* ≈0.66 mS cm^−1^ higher than other solid or hybrid Zn^2+^‐conductors. High electronic conductivity for Zn^2+^‐SIEs permits the reaction of Zn^2+^ with electrons to generate Zn‐dendrites in SEs at Zn‐plating potential, consistent with the Na‐β‐Al_2_O_3_ SIEs.^[^ ^523^ ^]^ The optimal fluorine doping concentration is 5.6 at%, beyond which it reduces ion conductivity due to enlarged surface adoption and decreased substitution of F^−^. The Zn^2+^‐conductors with Zn_2_SiO_4_ (0.028 mS cm^−1^), Bi_2_Zn_0.1_V_0.9_O_5.35_ (0.034 mS cm^−1^), and EMI‐TFSA@ZIF‐8 (0.019 mS cm^−1^) are also reported with low *E_a_* and high dielectric constants, which is favorable for Zn^2+^‐ions transport.^[^ ^524^, ^525^, ^526^ ^]^ Aliovalent anions substitution in ZnS lattice ensures the generation of anion substitutional defects with cation vacancy. Replacement of monovalent F to divalent S alters the charge distribution inside the ZnS by forming Vac_Zn_ and regulating electron densities for nearby anions and vacancies.^[^ ^527^ ^]^ Smaller ionic radius and strong zincophilicity of F trigger the lattice reduction and phase transitions from ZnS(111) to F‐ZnS(100). Vac_Zn_ strengthens the valence band maximum nearby the Fermi level.

**Figure 26:**
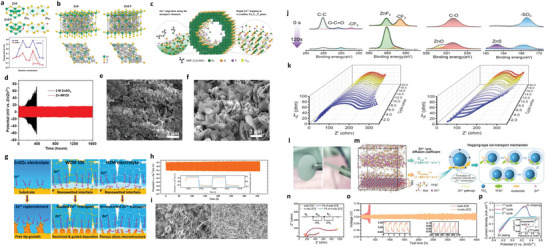
a) Diffusion path (top) and energy profiles (bottom) for Zn^2+^ in ZnS and ZnS‐F for neighboring octahedral Zn sites. b) Probability densities for the diffusion of Zn‐ions for ZnS (left) and ZnS‐F (right). c) Schematics for Zn^2+^ conduction in Zn‐MFZS SIEs. d) Zn//Zn plate/strip cycles. e,f) SEM images for Zn anodes after plate/strip cycles for Zn‐MFZS (e) and 2 m ZnSO_4_ (f). Reproduced with permission under the Creative Commons Attribution Non‐Commercial License 4.0 (CC BY‐NC).^[^ ^518^ ^]^ Copyright 2023, American Association for the Advancement of Science. g) Zn‐deposits kinetics. h) Zn/Zn cells under WZM SSEs. i) SEM images for Zn‐anodes after ZnSO_4_ (left) and WZM SSEs (right). Reproduced with permission.^[^ ^528^ ^]^ Copyright 2019, Elsevier. j) XPS spectra for different interphase elements after cycling. k) In situ EIS spectra for Zn//Zn cells for PH/MXene SPE (left) and PH SPE (right). Reproduced with permission.^[^ ^533^ ^]^ Copyright 2022, Wiley VCH. l) Bulk solid membrane. m) MD simulation with Zn^2+^ conduction pathways. n) EIS spectra. o) Zn//Zn plate/strip cycles. p) CVs. Reproduced with permission.^[^ ^534^ ^]^ Copyright 2022, Wiley‐VCH.

**Figure** **26**a–c shows Zn^2+^ diffusion kinetics among the oct‐sites (Zn_oct_) and tet‐S‐coordinated sites (Zn_tet_), in which Zn_tet_ exhibits an energy barrier of 0.27 eV and Zn^2+^ diffusion is trailed by Zn_oct_ barrier (0.38 eV).^[^ ^518^ ^]^ F‐doping for S‐sites obtains a lower energy barrier for Zn_oct_ of 0.31 eV with favorable ZnS lattice illustrating zincophilicity of F_S_ and improved steric effect of Vac_Zn_ with numerous Zn^2+^‐transport channels. Zn//Zn‐MFZS//Zn cells display stable electrochemical voltage polarizations for 1600 h with a cumulative capacity of 4000 mAh cm^−2^ and overpotential of 36 mV (Figure 26d). SEM images for Zn‐anode display compact surface with obvious ZnO and dendrites even after 1000 h under Zn‐MFZS SIEs, whereas under aqueous electrolyte severe formation of ZnO, Zn(HSO_4_)_2_ and Zn dendrites (Figure 26e,f). Wang et al.^[^ ^528^ ^]^ reported ZnMOF‐808 Zn^2+^‐solid conductor (WZM SEs) with *t_Zn_^2+^* ≈0.93 and *σ_Zn_^2+^* ≈0.21 mS cm^−1^ with Zn^2+^‐electrodeposition kinetics. In liquid, rapid refilling of depleted Zn^2+^‐ions occurs nearby tips due to faster long‐range movability for bulk phases that suppresses Zn‐growth of Cu host with enlarged vertical tip‐growth (uneven Zn deposits). But, WZM SEs exhibit sub‐nanochannels inside the MOF‐hosts with controlled transport of Zn(H_2_O)_6_^2+^ ions illustrating interfacial wetting and uniform/dense Zn^2+^‐depositions (Figure 26g). Zn//WZM//Zn cells display the low overpotential of 0.1–0.12 V with stable dendrite‐free Zn deposits/stripping over 350 h demonstrating interfacial compatibility of WZM SEs with Zn‐anodes (Figure 26h,i). Chen et al.^[^ ^529^ ^]^ presents the PVHF/MXene‐g‐PMA SPEs with *σ_Zn_^2+^* ≈0.269 mS cm^−1^ at RT and dendrite‐free stable Zn plate/strip cycle operations for 1200 h with 98.9% CEs. XRD confirms the no obvious ZnO or hydroxides after Zn plating. PVdF‐HFP/Zn(Tf)_2_ SPEs exhibit the formation of an interfacial layer, including the Zn and SPEs reaction products illustrating the rise of interface resistance and polarization, similar to the Li‐SPEs with suppression of dendrites.^[^ ^530^ ^]^ Carboxymethyl cellulose (CMC)/poly(N‐isopropylacrylamide) (PNiPAM) SPEs show large tensile strength and modulus, porous structure, which supports Zn^2+^ ions transfer in SPEs with *t_Zn_^2+^*  ≈0.56, *σ_Zn_^2+^* ≈0.168 mS cm^−1^, and excellent compatibility of SPEs and Zn‐electrodes over 150 h.^[^ ^531^, ^532^ ^]^ Theoretical calculations verify the influence of MXene nanofiller refrain local current density distributions and concentration of Zn ions.^[^ ^533^ ^]^ Local current density is more uniformly distributed with PH/MXene SPEs, causing lower polarization and homogeneous ion transport regulated by MXene. In situ stable organic/inorganic interface realizes the feasible interface transport kinetics and dense Zn deposits. Figure 26j displays interphase compositions, including C‐C (284.8 eV), C‐O (531.9 eV), and O═C‐O (289.2 eV) signals for C 1s, ‐CF_3_ (292.8 eV), ‐SO_3_ (169.2 eV), ZnS (162.3 eV), and ZnF_2_ (685.1 eV) for F 1s and S 2p spectra, which illustrates existence of organic (dominant) and inorganic (less) components. Ar^+^ etching (120s) weakens the C‐C, C‐O, and ‐SO_3_ and emerging ZnF_2_, ZnS, and ZnO species due to reductive decomposition of Zn(OTf)_2_/PVDF‐HFP complex benefiting the interface stability and ion‐transport. In situ EIS under PH/MXene SPE displays an initial decrease of interface resistance and is later stable for more cycles. In contrast, PH SPE shows a gradual increase in resistance, verifying the stable Zn and PH/MXene interfaces Figure 26k.^[^ ^533^ ^]^

High‐entropy eutectic networks or preferred adsorption of TFSI^−^ anions deteriorates the association of ions with forming Zn^2+^ permeated pathways over the nucleating TiO_2_ surfaces ensuring solid crystals with superior *t_Zn_^2+^* ≈0.57 and *σ_Zn_^2+^* ≈0.0378 mS cm^−1^ for ZCE SEs (Figure 26l,m).^[^ ^534^ ^]^ TFSI^−^ tethered regions provide the surplus interfacial ion‐conduction channels due to Lewis acid‐base interactions. Low energy shifting of Zn K‐edge after crystallization occurs owing to reduced electron transfer among the Zn and O (TFSI^−^), providing easier transport of Zn^2+^ towards the space‐charge region. Zn^2+^ diffusion kinetics for TiO_2_ interface is larger relative to the bulk crystalline phase (*D*_interface_ = 1.196 × 10^−5^ m^2^ s^−1^ > *D*_bulk_ = 7.980 × 10^−7^ m^2^ s^−1^) verifying the fast interface hopping of Zn^2+^. ZCEs tolerate small interface resistance (288 Ω) upon cycling, validating the exceptional solid‐solid interface compatibility (Figure 26n). Zn//Zn reversibility under in situ ZCE outperforms 4000 cycles (4000 h) with stable polarization voltage of 30 mV with dense and uniform Zn depositions with unchanged crystal structures (Figure 26o). This confirms the formation of stable SEI (components ZnF_2_ and organic S/N) with relieving accompanying strains. CVs vindicate the first voltage hysteresis of −0.077 V for Zn nucleation, implying the minimal energy loss for phase transitions among Zn^2+^‐ions and Zn‐metal under ZCE (Figure 26p). For feasible interface among the electrolyte/electrodes, construction of several in situ polymer electrolytes (PEO, poly(*N*‐methyl‐malonic amide, triethyl phosphate, poly(1,3‐dioxolane), PAM, TEP‐PC) have been demonstrated using photon‐ or ultraviolet‐assisted printing or methods, however, *σ_Zn_^2+^* and *t_Zn_^2+^*‐transfer kinetics is extremely poor.^[^ ^535^, ^536^, ^537^ ^]^ Extensive efforts have been devoted to the fabrication of SPEs and their interface chemistries with different polymeric hosts (PEO, PPO, PAM, CMCs, PVDF‐HFP, PAN, gelatin, PVA, Xanthan gum, PAA, hydroxyethylcellulose, PANa, TEGDA), Zn‐salts (Zn(Tf)_2_, Zn(CF_3_SO_2_)_2_, ZnSO_4_, ZnCl_2_, Zn(CH_3_COO)_2_, Zn‐TFSI_2_, Zn acrylate, Zn‐Otf_2_, Zn(BF_4_)_2_), ILs ([Emim]OTF, EMITf, EMIMTFSI, EMIM]BF_4_), and inorganic fillers (ZnO, Al_2_O_3_, Ti_3_C_2_T_x_, MOF, ZIF, MXene, SiO_2_).^[^ ^538^, ^539^, ^540^, ^541^, ^542^, ^543^, ^544^, ^545^, ^546^ ^]^

**Figure 27:**
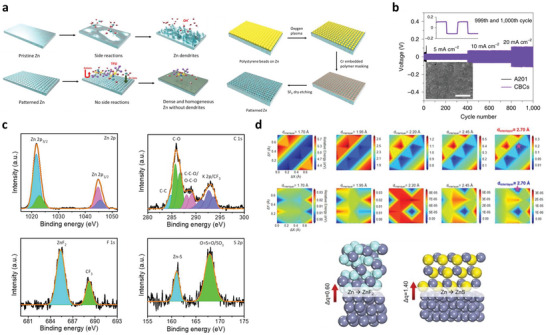
a) Schematics for Zn‐anode surface structures (left) and synthesis processes of patterned Zn‐anodes (right). b) Zn/Zn symmetric cells for different current densities and electrolytes. c) XPS patterns of Zn 2p, C 1s, F 1s, and S 2p elements for SEI layer over patterned Zn anode. d) Theoretical demonstration for interfacial reactions (top) and electron charge transfers (bottom; ZnF_2_@Zn, left and ZnS@Zn, right). Reproduced with permission.^[^ ^191^ ^]^ Copyright 2021, Nature Publishing Group.

**Figure** **27**a displays the interfacial reaction kinetics and design processes for patterned Zn‐anodes using SF_6_ plasma etching with sulfurized or fluorinated surfaces with ZnF_2_ and ZnS polar bindings with preferred 101 crystal orientation.^[^ ^191^ ^]^ Patterned Zn anode implied superior mechanical stability under stress‐strain and stable polarizations for ≥1000 cycles at current densities of 5–20 mA cm^−2^ without dendritic growth under chitosan‐biocellulosic SEs (CBCs), which illustrates the greater electrochemical compatibility of patterned Zn‐anode (Figure 27b). It shows robust SEI with electrostatic interactions with Zn^2+^‐ions and CBCs components. XPS spectra of C, O, Zn, F, and S reveal the presence of inorganic ZnF_2_ and ZnS species plays a key role in the stabilization of the Zn‐anode with even and compact nucleation process (Figure 27c). CBCs effectively accommodate the volume changes due to their excellent mechanical properties. EIS spectra also confirm the formation of the SEI layer for high/medium frequency region and minor Warburg impedance having ion diffusion kinetics. Theoretical results show the unbalanced charge distributions for interphase regions due to S and F bindings with Zn, which boosts Zn^2+^‐diffusion with enhanced mechanical reliance to suppress the corrosion reactions (Figure 27d).

**Figure 28:**
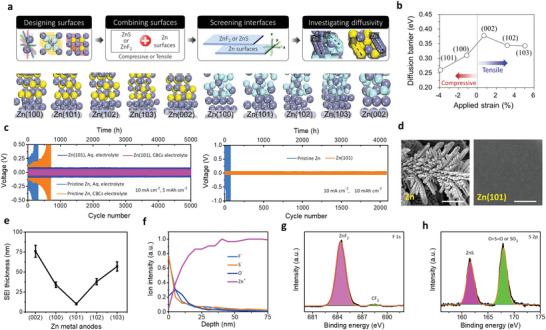
a) Theoretical demonstrations for the design of preferred Zn anodes from (100), (101), (102), (103), (002) crystal planes. Yellow and blue balls denote the S and F, respectively. b) Surface diffusion barriers with applied strains. c) Zn/Zn symmetric cells for different areal capacities of 5 mAh cm^−2^ (left) and 10 mAh cm^−2^ (right). d) SEM images of pristine (left) and Zn(101) (right) after 50 plate/strip cycles. e) SEI thickness versus Zn‐anodes. f) TOF‐SIMS depth profiles. g,h) XPS spectra of F 1s and S 2p over Zn(101) SEI layer compositions. Reproduced under the terms of a Creative Commons CC BY license.^[^ ^9^ ^]^ Copyright 2023, Wiley VCH.

Theoretical calculations reveal the strategies for projecting SEI formation, diffusion behaviors, and dendritic growth suppression using S and F‐chemistries (**Figure** **28**a).^[^ ^9^ ^]^ According to the F‐ and S‐loading levels from 5–30%, the Zn anode forms their preferred crystal structures from (002), (100), (101), (102), and (103). Zn(101) confirms the lowest surface diffusion barriers and high surface energies for F and S with minimum compressive strain relative to those of (002), (102), (103), and pristine Zn facilitating impulsive reorientation of Zn crystals and which is epitaxial with Zn surfaces and favorable for SEI (Figure 28b). Further, it shows 3D diffusion kinetics and absorption of Zn^2+^‐ions over Zn surfaces with a localized decrease to Zn^0^. Preferential crystal orientations are confirmed in the order of Zn(101) > Zn(100) > Zn(102) > Zn(103) > Zn(002) > Zn owing to the strength of ionic bonds and steric interactions inside the lattices. Zn(101)//Zn(101) cells exhibit the smallest polarization of 52 mV for 5000 cycles for a plating capacity of 5 mAh cm^−2^ and 10 mA cm^−2^, 2.5‐fold larger than commercial targets. It also demonstrates the commercial‐scale plating capacity operations (10 mAh cm^−2^ and 10 mA cm^−2^) for >2000 cycles, which illustrates the exceptional electrochemical interface stability (Figure 28c). EIS verifies the minimal interfacial resistance and stable operations required for depth of discharge of Zn‐based anodes. SEM images display a dense and compact surface for Zn(101). In contrast, the pristine Zn has severe dendritic growth and cracks (Figure 28d). Besides, the thinnest SEI of 10.2 nm was demonstrated for Zn(101) relative to those of other crystal planes, implying good structural stability, as comparable to commercial LIBs kinetics.^[^ ^266^, ^547^ ^]^ TOF‐SIMS and XPS validates the ZnF_2_/ZnS‐enriched SEI with presence of ≈684.4 (F 1s), ≈161.6 (S 2p), ≈1022.2 (Zn 2p), and ≈1045.2 eV (Zn 2p) peaks. Lower electronic conductance and high ZnF_2_/ZnS‐Zn interface energies justify a thin SEI (Figure 28e–h).^[^ ^548^ ^]^ Recently, several approaches for the development of Zn anodes, such as 3D structural design (sponge, MOF) and protection layers for Zn anodes also been reported to minimize the dendrite growth such as CNTs, MXene, carbon‐allotropes, graphene, metal alloys such as Hg, Au, Ag, Mg, Si, Sn, Al or metal oxides; however, such artificial buffer layers rigorously degrade the cell capacity and rate performances.^[^ ^549^, ^550^ ^]^

A comprehensive understanding of Zn‐interface chemistry reveals that poor ion‐conductance and electrode/SIEs interface are critical for practical applications for all‐solid‐state‐ZIBs. Insertion of ceramic fillers is the most common approach utilized to decrease the crystallinity of SIEs for improving Zn^2+^ migration or conductance. Ceramic fillers interact with a polymer matrix to construct the Zn^2+^‐conduction pathways, a higher dielectric constant improves the dissociation of Zn‐salts and forms numerous free Zn^2+^‐ions, and partial amorphous/crystalline structures mainly contribute overall conductivity. Lastly, the recently reported cellulose‐based composite SEs and preferentially oriented Zn‐anodes Zn(101) with chemical passivation of S, F, or others are the most effective strategies to reach commercial standards, including plating (10 mAh cm^−2^) and cumulative capacity (25 Ah cm^−2^) with standard current density (10 mA cm^−2^) for future Zn‐based battery technologies.

#### 7.2.3 Aluminum‐Ion Batteries (AIBs)

Aluminum metal offers the highest theoretical specific (2980 mAh g^−1^) and volumetric (8046 mAh cm^−3^) capacities, abundant resources, intrinsic safety, and low cost.^[^ ^551^, ^552^ ^]^ The major challenges include Irreversibility, undesirable parasitic reactions, dissolution of cathodes or anodes, and low CEs with inferior cycle life. AIBs research is primarily dedicated to liquid electrolytes. Solid‐state AIBs are just under the embryonic stage due to a lack of materials and resources for designing SEs with high Al^3+^‐conductivity with compatible anodes or cathodes. Wang et al.^[^ ^553^ ^]^ reported Al^3+^‐ion conduction pathways for NASICON‐type (Al_0.2_Zr_0.8_)_20/19_Nb(PO_4_)_3_ SEs using neutron diffraction and aberration‐corrected STEM spectroscopy. (Al_0.2_Zr_0.8_)_20/19_Nb(PO_4_)_3_ displays rhombohedral crystal structures including (Zr,Nb)O_6_ for oct‐sites sharing corners with (PO_4_) for tct‐sites and Al occupies interstitial trigonal anti‐prisms revealing larger displacements, which illustrates the random distribution of Al^3+^‐ions for c‐axis and vacancies promoting Al^3+^‐ion transport channels. EIS results confirm the feasibility of SEs with Al anodes with *σ_Al_^3+^* of 10^−4^–10^−6^ S cm^−1^ for 300–600 °C and *E_a_* of 3.1 eV. Al^3+^‐ion conductance ascribes the weak chemical interactions among the Al and O_2_ in the lattices with Al–O_2_ distances of 2.64–2.65 Å. The Sn_0.92_Sb_0.08_P_2_O_7_ SEs for anhydrous hydroxide‐ion conductor (*σ_hydroxide_* of 0.01 S cm^−1^) or Al–air batteries has been reported, in which upon discharge Al is oxidized to aluminate species, and it reduced to Al upon charge, verifying the SEs compatibility with Al‐metal.^[^ ^554^ ^]^ Poor intercalation barrier of electrolytes and lack of SEI are the major failures kinetics for the AIBs.^[^ ^555^ ^]^ Literature shows the much effort for ILs (50–90 wt%) encapsulated polymer electrolytes such as PVDF, PEO, PAM, PEA, or derived MOFs as GPEs for AIBs; however, poor charge‐/mass‐transport kinetics for polymers or MOFs, sluggish Al^3+^‐ion diffusion, and high interfacial resistance for Al//SEs limits their use for AIBs.^[^ ^556^, ^557^, ^558^ ^]^ Shen et al.^[^ ^559^ ^]^ reported that soft GPEs provide excess nucleation sites for Al deposits, illustrating the better Al^3+^‐plate/strip for soft GPE than rigid GPEs with fractal branched Al‐dendrites. PEA‐GPE displays *σ_Al_^3+^* ≈1.46 mS cm^−1^ and EWs ≈3 V versus Al/Al^3+^ with minimal charge‐transfer resistance implying the stable Al//Al interface polarizations.^[^ ^193^ ^]^ Further, photo‐curable PTHF‐Epoxy SPEs have been reported with *σ_Al_^3+^* ≈0.02 mS cm^−1^.^[^ ^560^ ^]^

Besides, to alleviate the passivation effect of native oxide and electrode disintegration that undergoes severe parasitic reactions with continuous depletion of electrolyte even for lower overpotentials, several approaches have been proposed, such as uniform protective Al oxide layer over Al‐metal, interface engineering, specifically artificial SEI or buffer layers. However, the formation of ionically insulated native oxides over Al renders unfeasible Al plate/strip, the high bandgap of passivating oxides hinders electrons and ions‐transfer across the interface, thick artificial SEI increase the interface resistance and voltage polarizations with extremely poor Al^3+^‐plate/strip, CEs severely degrades the cell performances. It is highly desirable to fabricate new Al‐anodes with high reversibility, new SEs with better interface compatibility, and Al‐alloyed anodes. In this context, several modified Al‐anodes have been reported, such as Au‐Al, Al_82_Cu_18_, ACNI/Al‐15, Al_0.265_TiO_2_, MoO_3_, TiO_2_, Al_97_Ce_3_, MXene/E‐ Al_97_Ce_3_, organic or hybrid materials.^[^ ^561^, ^562^, ^563^ ^]^ Theoretically AIBs are promising candidates, however, reported practical results are significantly out of scope for large‐scale energy applications. Extensive efforts are critically required to develop new compatible cathodes, anodes, and electrolyte materials. The knowledge and in‐depth investigation are critically needed for future AIBs in terms of: for SEs, high Al^3+^‐ion conductivity with superior de‐/intercalation of ions for both electrodes and broader EWs; for cathodes, tight interactions among the Al^3+^‐ions and structures shows sluggish diffusion kinetics, numerous active surfaces, and wide surface area or porosity; for Al anodes, highly reversible without formation of oxide passivation and strong corrosion reactions. Theoretical evaluations of complicated electrochemical reaction kinetics of AIBs regarding structural and electrochemical properties for SEs, Al‐anodes, and cathodes are crucial to guide experimental research. We described various mono‐/multivalent battery chemistries under SEs for their anode/SEs interface compatibilities.

#### 7.2.4 Calcium‐Ion Batteries (CIBs)

**Figure 29:**
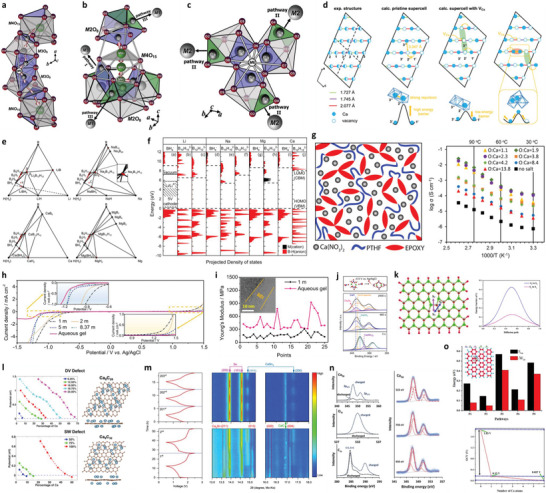
a‐c) Ca^2+^‐migration pathways I (a), II (b), III (c) in β‐Ca_3_(PO_4_)_2_ and Ca_10.5‐x_Pb_x_(VO_4_)_7_, respectively. Reproduced with permission.^[^ ^572^ ^]^ Copyright 2018, Elsevier. d) Schematics for Ca‐ordering and occupied sites in Ca_1.5_Ba_0.5_Si_5_O_3_N_6_ along (010) orientations. Reproduced with permission.^[^ ^573^ ^]^ Copyright 2022, American Chemical Society. e) Phase diagrams of Li‐B‐H (top left), Na‐B‐H (top right), Ca‐B‐H (down left), and Mg‐B‐H (down right) systems. Red squares indicate metastable phases (ground state energy <80 meV/atom above the hull). α is the LiB_10_H_9_, β the Li_2_B_10_H_10_, γ the LiB_3_H_8_, δ the Na_2_B_10_H_10_, ε the Na_2_B_6_H_6_, and ζ the NaB_3_H_8_. f) Partial density of states for Li, Na, Mg and Ca with B‐H anions relative to stable/metastable phases. Dashed lines are VBMs and CBMs. Reproduced with permission.^[^ ^575^ ^]^ Copyright 2017, American Chemical Society. g) PTHF‐Epoxy cross‐linked structures (left) and ion conductivity of PTHF‐Epoxy SPEs (right). Reproduced with permission.^[^ ^578^ ^]^ Copyright 2019, American Chemical Society. h) EWs of electrolytes. i) Young's modulus of S/C anodes in aqueous gel electrolytes. Inset STEM image showing SEI. j) DFT calculations and in‐depth Ca 2p XPS spectra. Reproduced under the terms of a Creative Commons CC BY 4.0 license.^[^ ^583^ ^]^ Copyright 2021, Nature Publishing Group. k) Energy barriers for Ca‐diffusion over SW‐BN anodes. Reproduced with permission.^[^ ^589^ ^]^ Copyright 2023, Elsevier. l) Calcination potential for adsorbed Ca percentages for DV and SW effects. Reproduced with permission.^[^ ^591^ ^]^ Copyright 2014, American Chemical Society. m) In‐situ XRD for Ca‐Sn alloy anode phase analysis upon electrochemical process. Reproduced under the terms of a Creative Commons CC BY 4.0 license.^[^ ^592^ ^]^ Copyright 2022, Nature Publishing Group. n) XPS spectra charge‐discharged graphite for −2.9 and 0.2 V (left) and synchrotron XPS for various photon energies (right). Reproduced under the terms of a Creative Commons CC BY license.^[^ ^593^ ^]^ Copyright 2019, Wiley VCH. o) Migration pathways (top) and OCVs of BC_3_ with Ca concentrations. Reproduced with permission.^[^ ^594^ ^]^ Copyright 2022, Elsevier.

CIBs offer significant attention due to their lower redox potential for Ca/Ca^2+^ (−2.87 V vs SHE closer to Li/Li^+^) and smaller charge density and polarization strength with high power and diffusion kinetics compared to those of other multivalent ions and high gravimetric (1337 mAh g^−1^) and volumetric (2073 mAh cm^−3^) capacities. However, sluggish ion diffusion‐kinetics, the irreversible Ca^2+^‐metal plating/stripping, operating at high temperatures only, and undesired parasitic reactions with forming CaF_2_, CaCl_2_, CaCO_3_, and CaH_2_ lead to continuous growth of passivated film over Ca‐anodes and poor CEs. Limited development for high‐performance cathodes, SEs, and anode materials is another challenge for CIBs.^[^ ^564^, ^565^, ^566^, ^567^ ^]^ Thus SEs received critical attention for CIBs. Seevers et al.^[^ ^568^ ^]^ developed the Ca^2+^‐β″‐Al_2_O_3_ SEs with *σ_Ca_^2+^* ≈36 mS cm^−1^ for 300 °C. Katsuhiro et al.^[^ ^569^ ^]^ displays the M^II^Zr_4_(PO_4_)_6_ (M^II^ = Mg, Ca, Sr, Ba, Mn, Co, Ni, Zn, Cd, Pb) NASICON SEs with *σ_Ca_^2+^* ≈1.4 µS cm^−1^ and migration barrier of 146 kJ mol^−1^ for 800 °C. The Ni, Co, Mg, and Zn–based materials display order‐disorder transitions among 600 and 720 °C with β‐Fe_2_(SO_4_)_3_‐type structure, whereas Ca, Cd, Ba, Sr, and Pb possess NASICON‐type structures without phase transitions among RT and 1000 °C. Mn‐based SEs display transitions at 560 °C. Deyneko et al.^[^ ^570^ ^]^ reports the whitlockite‐type Ca_10.5−x_Pb_x_(VO_4_)_7_ (x = 1.9, 3.5, 4.9) Ca^2+^‐ion conductors with *σ_Ca_^2+^* ≈0.1 mS cm^−1^ for 800 K, and *E_a_* ≈ 1.3–1.5 eV. ACa_9_(VO_4_)_7_ (A = Gd, Ho, Lu, Er, Eu, Pr, Sm, Bi, La, Nd, Tb, Yb, Y, and Sc), Ca_7_MgPbBi(VO_4_)_7_, Ca_7.5_ZnPb_0.5_Bi(VO_4_)_7_, Ca_7.5_CdPb_0.5_Bi(VO_4_)_7_, Ca_8_PbBi(VO_4_)_7_, Ca_3_(VO_4_)_2_, Ca_7.5_Pb_3_(VO_4_)_7_, Ca_6.5_Pb_4_(VO_4_)_7_, Ca_6.5_Pb_4.5_(VO_4_)_7_ Ca^2+^‐conductor materials with *σ_Ca_^2+^* ≈10^−11^–10^−2^ S cm^−1^ for 300–1700 °C have been also reported. *σ_Ca_^2+^* increased by ten times with insertion of Pb from 0–4.5 in Ca_10.5‐x_Pb_x_(VO_4_)_7_ with ≈7% volume expansion of unit cells. The presence of immobile atoms (Bi, Pb, Mg, Zn, or others) in the lattices negatively influences Ca^2+^‐conductivity owing to obstacles for Ca^2+^‐pathways.^[^ ^571^, ^572^ ^]^ The Ca^2+^‐migration pathways are of (I) … →M4→M3→M6→M3“→M4”→ …, (II) … →M2→M4→M2′, and Pb^2+^ based materials follow the (III) … →M2→M43→M2“→M1→M6→M1”→M2“”→ …. (**Figure** **29**a–c).^[^ ^572^ ^]^ Chen et al.^[^ ^573^ ^]^ reported the Ca_1.5_Ba_0.5_Si_5_O_3_N_6_ SEs to determine the Ca^2+^‐migration kinetics using neutron diffraction and AIMD simulations. The Ca^2+^ partially occupies three distinct crystallographic positions with smaller distances among the adjacent Ca sites (1.727–2.077 Å) along the same plane, manifesting determined 1D Ca^2+^‐migration (Figure 29d). AIMD verifies the ≈400 meV Ca^2+^‐migration barrier and “vacancy‐adjacent” concentrated ion‐transport kinetics. Lei et al.^[^ ^574^ ^]^ reports the GO_[Ca]_ SEs with *σ_Ca_^2+^* ≈1.08 mS cm^−1^ and ion diffusion accelerated from 0.6 to 9.8 µS cm^−1^. Fluorinated alkoxyaluminates Ca[Al(HFIP)_4_]_2_ electrolytes with high anodic stability (>3–5 V vs Ca/Ca^2+^) are also reported.

Theoretic calculations show ternary phase diagrams by constructing A‐B‐H (A = Li, Na, Mg, Ca; Figure 29e) to fabricate metal borohydride SEs. ABH_4_ and A_2_B_12_H_12_ (A = Li, Na) are thermodynamically stable, whereas B_10_H_10_^2−^ are metastable phases.^[^ ^575^ ^]^ Anions in Li‐hydrides are cubic close‐packed/fcc, while hcp is for Na‐counterparts. Few metastable phases are observed for Ca and Mg. BH_4_^−^ and B_12_H_12_^2−^ based disordered phases are thermodynamically stable in Ca and Mg. B_12_H_12_^2−^ in CaB_12_H_12_ rotates for >1500 K. At large potentials, Ca and Mg show good stability against electrochemical oxidation >5 V with low diffusion constants (Figure 29f). Koettgen et al.^[^ ^576^ ^]^ also reported CaB_12_H_12_ Ca^2+^‐conductor with an activation barrier of 650 meV, relatively larger than Li/Mg (<400 meV). Lee et al.^[^ ^577^ ^]^ shows the insertion of Ca^2+^‐ions in HfNb(PO_4_)_3_ lattice forms the NASICON‐type Ca^2+^‐SEs. The high‐valence cations (Hf^4+^, Nb^5+^, and P^5+^) realize the severe decrease in electrostatic interactions for Ca^2+^‐ions inside the structures. (Ca_0.05_Hf_0.95_)_4/3.9_Nb(PO_4_)_3_ SEs display *σ_Ca_^2+^* ≈10^−6^–10^−2^ S cm^−1^ from 600–1000 K with smaller *E_a_*. Figure 29g shows the photo‐cross‐linked SPEs with polytetrahydrofuran (PTHF) and 3,4‐epoxycyclohexylmethyl‐3′,4′‐epoxycyclohexane carboxylate with *σ_Ca_^2+^* ≈0.126 mS cm^−1^ (O:Ca ratio of 1.9:1) for RT, *E_a_* ≈0.328 eV, and *t_Ca_^2+^* ≈0.359 at 70 °C.^[^ ^578^ ^]^ Raman peak of 1050 cm^−1^ manifests the dissociation of nitrate anions owing to increased salt concentration, which illustrates the Ca^2+^ complexation to oxygen with appropriate solvation of calcium nitrate in SPEs with compatible Ca^2+^‐ion transports.^[^ ^579^ ^]^ Genier et al.^[^ ^580^ ^]^ reports the ethylene oxide to calcium ratios EO/Ca from 5 to 52 to obtain 3D cross‐linked PEGDA‐Ca SPEs. The EO/Ca ratio of 5 displays *σ_Ca_^2+^* ≈3 µS cm^−1^, *E_a_* ≈0.25 for RT and 0.34 mS cm^−1^ for 110 °C. *T_g_* of PEGDA‐Ca increases from −13.81 to 78.61 °C for pristine PEGDA to EO/Ca of 52–5. Biria et al.^[^ ^581^ ^]^ displays the PEGDA‐1‐Ethyl‐3‐methylimidazolium trifluoromethanesulfonate‐GPEs with *σ_Ca_^2+^* ≈10^−4^–10^−3^ S cm^−1^ for RT to 110 °C, EW of ≈4 V versus Ca/Ca^2+^, *t_Ca_^2+^* ≈0.17, and thermal stability of 300 °C with discharge capacity of 140 mAh g^−1^. Further, the Ca_x_M_2_(ZO_4_)_3_ (where M = Ti, V, Cr, Mn, Fe, Co, or Ni and Z = Si, P, or S) NASICON‐based Ca‐cathodes also reported.^[^ ^582^ ^]^ Theoretical calculations reveal the Ca_x_V_2_(PO_4_)_3_, Ca_x_Mn_2_(SO_4_)_3_, and Ca_x_Fe_2_(SO_4_)_3_ Ca‐NaSICONs are favorable Ca‐cathodes.

The polyvinyl alcohol (PVA) and Ca(NO_3_)_2_‐based aqueous‐gel‐electrolytes (AGEs) show stable EWs ≈2.6 V to fulfill redox couples of S/C‐anode and Ca_0.4_MnO_2_ cathodes compared to liquid 1m Ca(NO_3_)_2_ electrolyte (Figure 29h).^[^ ^583^ ^]^ With PVA, Ca^2+^‐H_2_O complexes display polymer‐like accumulation illustrating numerous H_2_O molecules immobilization via PVA chains and highly‐concentrated‐Ca‐salts with suppressing diffusion of polysulfides.^[^ ^584^ ^]^ Amorphous SEI with ≈10 nm thickness in AGEs is obtained due to participation of PVA‐matrix, SEI‐S/C anode exhibits Young's modulus of ≈445 MPa larger than liquid 165 MPa, which illustrates the structural integrity against electrode deformations (Figure 29i). DFT and XPS verifies the coordination of three NO_3_^−^‐anions with a Ca^2+^ solvation sheath and preferential reduction of NO_3_^−^‐anions facilitates construction of SEI in AGEs. Ca 2p XPS spectra demonstrates presence of CaO, Ca_3_N_2_, Ca–S (CaS or Ca(HS)_2_), CaCO_3_, and Ca(NO_3_)_2_ and enhancement of intensity for CaO, Ca_3_N_2_, and Ca–S species and reduction for CaCO_3_ with etching time manifesting CaCO_3_ is in outer‐layer of SEI and CaO, Ca_3_N_2_ are major components of SEI inner‐layer (Figure 29j). This inorganic SEI inhibits the polysulfide dissolution and HER.^[^ ^585^ ^]^

Initially, the electroplating of Ca and Mg failed owing to poor diffusion of divalent cations through passivation layers (M‐SOCl_2_, M = Ca or Mg) with typical formation of Ca^2+^‐surface‐blocking films restricting the Ca^2+^‐deposition.^[^ ^586^ ^]^ Several efforts have been performed to reversible Ca^2+^‐plating/stripping from developing Ca‐based anodes, electrolytes, and proper solvation chemistries. Tran and Obrovac show metal‐alloy anodes for various A_x_M batteries (A = Li, Na, K, Mg, Ca, and Al) in terms of volumetric energies and electrode expansion kinetics and revealed the Ca_2_Si and Ca_2_Sn alloys have larger volumetric energies of 5690 and 4317 Wh L^−1^ than LIBs anodes, which manifests Ca‐Si or Ca‐Sn is favorable anodes for CIBs.^[^ ^587^ ^]^ Ponrouch et al.^[^ ^588^ ^]^ reports the electrochemical feasibility for Ca^2+^‐de‐calciation of Ca^2+^ for Ca–Si intermetallic alloy with 306% volume expansion by inserting Ca^2+^ in the fcc‐Si causes structural failure. The Ca‐diffusion in Stone‐Wales boron nitride (SW‐BN) surfaces possess two feasible directions along a‐ and b‐axis (Path‐I and II), in which Ca diffuses among the two adjacent more stable hollows (S1 → S1, Path‐I) and another hollow (S1 → S2, Path‐II). Diffusion barriers for path‐I and II are 0.11 and 0.49 eV, respectively, which indicates the Ca‐transport for SW‐BN is superior in path‐I (Figure 29k).^[^ ^589^ ^]^ Woodcox and Smeu report the elastic properties DFT calculations for Ca_x_Sn_1‐x_ alloys anode with four stable (x = 0.25, 0.5, 0.625, 0.75) and three metastable (x = 0.5 (two), 0.75 (one)) phases. It shows shear, Young's, and bulk modulus in the range of 21.6–25.3, 56.1–59.1, and 25.7–46.3 GPa, respectively.^[^ ^590^ ^]^ Ca‐atoms are mainly positioned closer to O places (i.e., adatom clusters nearby defective zone) for divacancy, and adsorption percentage enhances with a rise in defect density for SW defects (Figure 29l).^[^ ^591^ ^]^ Figure 29m demonstrates the phase evolution of Ca_x_Sn anodes under 0.5–2.5 V. For the first cycle, the Ca_2_Sn peak decreases upon onset of decalciation and then continuously diminishes intensity with signals of β‐Sn upon discharge, whereas, CaSn_3_ peaks newly emerged upon charge. Further, the reversible de‐/calciation of CaSn_3_ is verified for repetitive charge‐discharge cycles. SEM displays pristine Ca_x_Sn aggregates particles of 1–30 µm, while after the 3000^th^ cycle's porous and rod‐shaped crystalline solids (100–300 nm).^[^ ^592^ ^]^ XPS clarifies the existence of Ca^2+^ in the graphite surface by surface adsorption and SEI. Synchrotron XPS displays two Voigt functions of surface Ca^2+^ and intercalated Ca‐G_4_ peaks for 346.6 and 347.5 eV (Figure 29n). By changing photon energy from 523–950 eV, gradual decrease for surface Ca^2+^ signals is observed implying the discharged‐state Ca‐peaks correlates for surface Ca^2+^ in XPS. EDS and XPS also justify the higher quantity of Ca in charged graphite compared to discharged graphite and verify the simultaneous increase/decrease for O‐contents facilitating reversible de‐/intercalation of Ca^2+^ with G_4_.^[^ ^593^ ^]^ Energy barriers and adsorption energies of P1, P2, P3, P4, and P4 are 0.214, 0.147, 0.571, 0.247, 0.458 eV and 0.08, 0.05, 0.41, 0.11, 0.37 eV, respectively for Ca adsorbed boron carbide (BC_3_) monolayer. The charge distribution, lattice distortion, electronic, and steric hindrance severely influence the migration pathways. P1 (P4) shows small adsorption energy and high migration barrier due to considerable changes in distributed charges and lattice structures. OCV of BC_3_ is 1.85 V and gradually decreases with Ca‐concentration from 1.85 to 0.027 for 0 to 6 atoms (Figure 29o).^[^ ^594^ ^]^ Numerous anodes including Sn‐In_2_O_3_, BC_3_, tetracarboxylic diimide (PTCDI), Ca‐Mg, Ca‐Sn, Ca‐Si, Ca‐Bi, 3,4,9,10‐perylenetetracarboxylic dianhydride (PTCDA), [Ca‐(DMAc)_4_]C_50_ ternary graphite‐intercalation compounds, Ca_3_Zn, CaLi_2_, Ti_3_C_2_, MCMB, and highly oriented pyrolytic graphite (HOPG) have been investigated, however, Ca^2+^‐diffusion, irreversibility for plate/strip, de‐/solvation kinetics, and passivated interfaces are major challenges of CIBs anode materials.^[^ ^595^, ^596^, ^597^, ^598^, ^599^, ^600^ ^]^ In summary, the rational design of SEs allows stable SEI formation on Ca‐surface with high‐ion conductivity and wide EWs. Comprehensively, CIBs are in an early stage of development due to a lack of electrode/electrolyte materials that can operate at RT. The attractive ways for Ca‐anodes are alloying or intercalating carbon‐based materials with lower stable potentials. Ca‐Sn alloy suggests promising anodes compared to Mg‐/Na‐ions. To obtain high‐energy CIBs, compatible alloy anodes must be coupled with high redox‐voltage/capacity cathodes. Close‐packed structures typically deliver high energies, up to now reported materials inhibit the reversibility of Ca^2+^‐ions upon de‐/calciation owing to thermodynamic instabilities of Ca^2+^‐ions in the lattice structures. Performance metrics for these battery chemistries and spectroscopic techniques for interfaces are outlined in **Tables** **4** and **5**.

**Table 4:** State‐of‐the‐art performances for different SSBs chemistries.

Battery systems	Gravimetric energy densities based on active materials mass (Wh kg^−1^)	Cell voltage (V)	Cycle life (number of cycles)	Cycle retention (%)
Li‐metal	300–800	3–4.5	100–400	50–60
Lithium‐sulfur	1500–1800	2–2.3	100–300	20–40
Na‐metal	200–300	3–4	100–250	20–40
K‐ion	250–400	3–3.5	100–300	20–40
Mg‐ion	50–120	0.8–1.2	50–100	20–30
Zn‐metal	300–500	1–1.2	100–250	30–40
Al‐ion	100–200	1.7–2.2	50–100	20–30
Ca‐ion	<20	0.8–1.2	<20	20–30

**Table 5:** State‐of‐the‐art spectroscopy techniques for interface analysis of SSBs.

Spectroscopy techniques	Capability	Sample requirements	Limitations
Time of flight secondary ion mass spectroscopy (ToF‐SIMS)	Elemental and molecular ion quantification	Size‐ 1–1.2 cm^2^, Thickness‐ <10 cm, Insulator or conductor	Lower quantitative sensitivity
Ar^+^‐X‐ray photoelectron spectroscopy (XPS)	Element quantification, chemical states	Thickness‐ <1 mm	Weak lateral resolution ≈3 µm
Synchrotron XPS	Chemical core‐states and elemental quantification	Special cell design	Inert for Li
X‐ray absorption spectroscopy (XAS)	Element quantification, Chemical and structural kinetics	Special cell design	Inert for Li
X‐ray reflectivity (XRR)	Thickness, interface layers, density	Smooth surface	No element detection
Neutron‐depth profiling (NDP)	Quantification of interlayer formation, Active finding for Li‐ions	Thickness‐ 4.5 µm Roughness‐ <10 nm Proper stoichiometric	Depth of detection limits for <150 µm
Rutherford backscattering spectroscopy (RBS)	Film density, impurity profiles, the interaction of inter‐diffusion	Smooth surface, good for heavy elements, insulator or conductor	Expensive, Inter‐element interface, depth of detection < 1 mm
Auger electron spectroscopy (AES)	Elements or ions, chemical and atomic structure quantification	Thickness‐ <12 cm, Size‐ 1–3 cm^2^, only conductors,	Atomic detection limit −1%, Inert for chemical valence
Nuclear reactions analysis (NRA)	Elements, isotope, and concentrations quantification	Good for light elements in heavy matrix, Insulator or conductors	Expensive, detection limit <150 µm
Glow discharge optical emission spectrometry (GD‐OES)	Elemental quantification, Faster sputtering rates, Lower cost	Diameter‐ >4 mm No size limit, Thermal and mechanical stability, Both insulator or conductors	Poor lateral resolution ≈1 mm, Inter‐elements interferences
Laser ablation inductively coupled plasma mass spectrometry (LA‐ICP‐MS)	Elemental quantification, Faster sputtering rates	Diameter‐ <40 mm Thickness‐ <20 mm, Insulator or conductors	Poor lateral resolution ≈10 mm, Inter‐elements interferences
Nuclear magnetic resonance (NMR) with magnetic resonance imaging (MRI)	Quantify structures of SEI, chemical shifts, Microstructure of dendrites or deposits	Thermal and mechanical stability, Both insulator or conductors	Limited for a few elements

## 8 Comparison of Anode Chemistries in Liquid Counterparts

Unlike conventional graphite or Si, Li‐metal anodes are highly reactive with organic liquid electrolytes. Therefore, the salts and solvents from electrolytes promptly reduce with Li‐metal contact by forming an SEI layer over the surface of Li‐metal. Typically, the SEI layer comprises primarily inorganic species (Li_2_CO_3_, Li_2_O, and LiF) for the inner layer, whereas organic species (ROLi, ROCO_2_Li, and RCOO_2_Li) are in the outer layer, which significantly depends on the electrolyte compositions.^[^ ^601^ ^]^ Ideally, SEI features ion‐conduction and electronic‐insulation behavior that prevents straight interaction among the Li‐metal (or others) and electrolytes; however, it permits migration of Li^+^‐ions (or others) without decomposition of electrolyte and offers uniform Li^+^‐ions (or others) distribution with homogeneous metal‐depositions. In practice, the SEI layer is brittle, uneven, and their components did not accommodate the volume expansion of M^+^‐metals upon M^+^‐plate/stripping. Hence, the SEI layer is certainly ruined upon volume changes triggering severe cracks for SEI. Li^+^‐ions display preferential plating, and current density localizes over the cracks due to lower interfacial resistance of newly open Li‐surfaces at cracks than Li‐surfaces shielded with intact SEI. Thus, Li‐metal protrusions rise inhomogeneously from crack regions implying heterogeneous Li^+^‐plating. Besides, freshly plated Li‐metal is highly reactive with electrolyte depletion, forming additional SEI over new Li^+^‐depositions. This repetitive demolition and restructuring for SEI initiated from uneven Li‐plate/stripping causes constant depletion of Li‐metals and electrolytes, manifesting poor CEs and larger interfacial resistances.^[^ ^602^, ^603^, ^604^ ^]^

**Figure 30:**
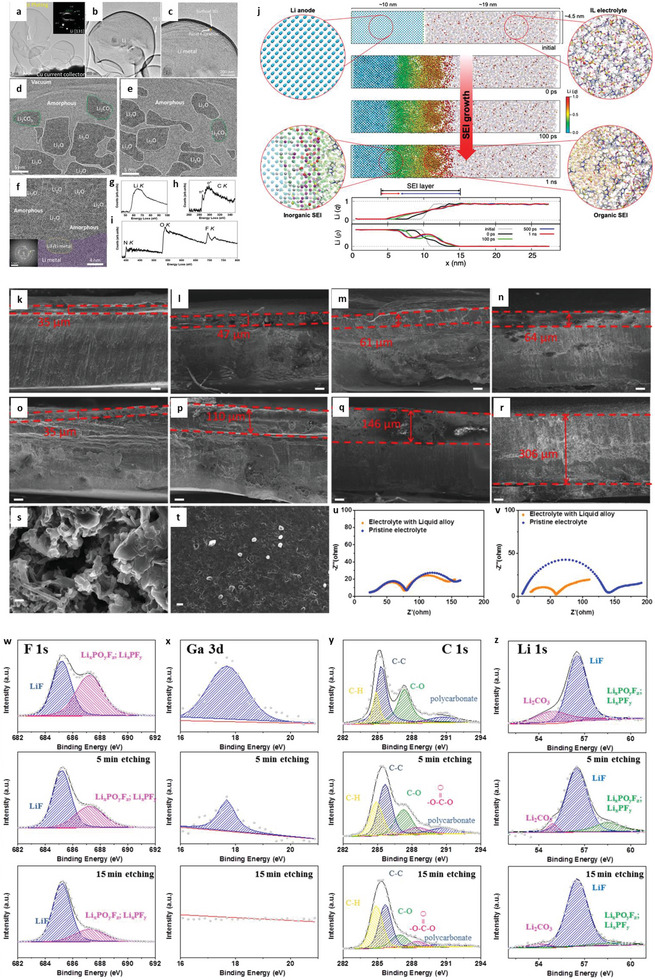
a‐f) TEM images and g‐i) Li, (g) C (h), N, O, and F (i) K‐edges from SEI. Reproduced with permission.^[^ ^605^ ^]^ Copyright 2022, Wiley VCH. j) SEI formation by MD simulations in [TFSI][BMIM] IL electrolyte at 300 K. Reproduced with permission.^[^ ^608^ ^]^ Copyright 2023, Wiley VCH. k‐r) SEM images with (k‐n) and without (o‐r) GaSnIn additives in convectional electrolyte. s,t) SEM images of Li‐plate in pristine (s) and GaSnIn additives (t) electrolytes. u,v) EIS epctra before/after cycles. w‐z) XPS depth profiles of F 1s (w), Ga 3d (x), C 1s (y) and Li 1s(z). Reproduced with permission.^[^ ^609^ ^]^ Copyright 2021, Elsevier.

Han et al.^[^ ^605^ ^]^ reports the electroplating for lithium bis‐trifluoromethanesulfonylimide (LiTFSI)−1,3‐dioxolane/1,2‐dimethoxyethane (DOLDME) electrolytes (1.0 m LiTFSI with 1:1 v/v DOL/DME and 1% LiNO_3_), which displays the generation of Li_metal_ spherical particles with Li_bcc_ single‐crystals along (131) and Li_2_O (111) diffractions. TEM images show dark exoskeleton and light‐contrast Li_metal_ with a 20 nm transition zone from SEI exoskeleton to inner crystalline Li_bcc_ and amorphous Li‐matrix (Li_amorphous_) consisting of nanocrystalline Li_2_O, LiF, or other amorphous phases. Inorganic Li_2_O and Li_2_CO_3_ phases are in the SEI exoskeleton inside the organic polymer matrices (**Figure** **30**a–f). The F, O, N, and C participate from electrolytes parasitic reactions with half‐cell reactions (e^−^(metal) + Li^+^(SEI) = Li‐atoms in Li_metal_), illustrating amorphous Li_metal_ nearby SEI exoskeletons. Polymeric elements for SEI show partial decomposition for LiC_x_ and Li_2_O and C, N, F, O for LiF and Li_2_O. Li, C, N, O, and F K‐edge spectra feature mixed valence states for SEI (LiC_x_, Li_2_O, LiF, Li‐polymer, π*(C═C) and σ*(C‐N, C‐O, C‐C), Figure 30g–i).^[^ ^606^, ^607^ ^]^ Theoretical calculations report ≈10 nm SEI consists of densely ordered inorganic layer (≈2.5 nm enriched with C and F) closer to Li‐metal anodes and porous organic layers (≈7.5 nm enriched with O and F) closer to electrolytes in [bis(trifluoromethyl‐sulfonyl) imide][1‐butyl‐3‐methylimidazolium] ([TFSI][BMIM]) ionic liquid electrolytes (Figure 30j).^[^ ^608^ ^]^

Figure 30k–r displays the corrosion depths of Li‐metal with/without GaSnIn additives in conventional LiPF_6_‐EC/DMC electrolytes.^[^ ^609^ ^]^ Upon first cycle, the corrosion layer thickness is similar in both electrolytes. In contrast, the 10 h cycle's thickness in conventional electrolytes is slightly larger and much higher after 100 and 250 h (582 µm) cycling, which causes cell failure. Massy dendrites, porous, and loosely bound structures with increased contact areas are clearly seen for LiPF_6_‐EC/DMC after 50 cycles, implying exacerbated side‐reactions with electrolyte depletion (Figure 30s). Whereas, the compact and smooth morphology without dendrites are observed for GaSnIn‐based liquid electrolytes, manifesting suppression of Li dendritic‐growth (Figure 30t). EIS displays stable interfacial impedance (charge‐transfer and Li‐ion diffusion resistances) for GaSnIn‐based electrolyte after long‐operations (Figure 30u,v).^[^ ^610^ ^]^ XPS spectra reveal C‐H, C‐C, C‐O, CO_3_^2−^, and polycarbonate groups, implying the presence of organic and inorganic species. SEI displays surface layer is enriched with Li_x_PF_y_, Li_2_CO_3,_ and the inner layer with LiF, illustrating the importance of the depletion of LiPF_6_ (Figure 30w–z). XPS confirms the formation of gradient SEI layers with GaSnIn additive with flexible polycarbonate surface‐rich and inorganic LiF‐rich cores with lower Li^+^‐partial molar volume. The weaker binding for LiF‐rich inner SEI to Li effectively facilitates lateral diffusion of Li with the prevention of Li‐dendrites from cracking SEI layers, whereas the outer polycarbonate‐rich flexible layer controls the integrity of other ionic‐conductive SEI elements and adhesion for bulk Li‐anodes.

**Figure 31:**
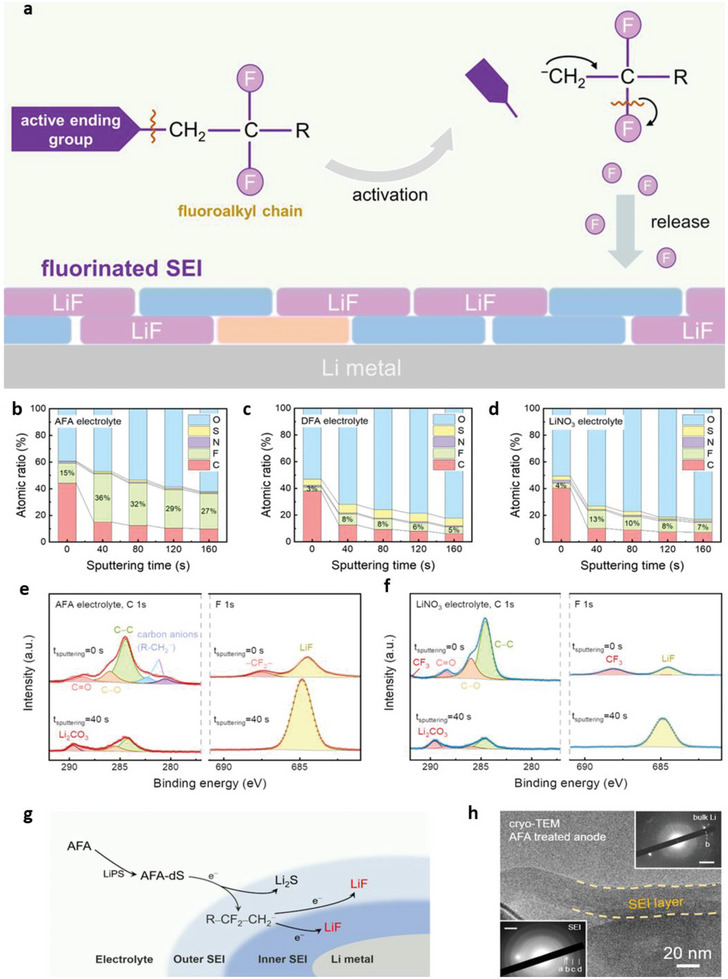
a) Fluorinated molecule synthesis scheme. Atomic concentrations of F,O,C,N, and S in formed SEI layer with b) AFA, c) DFA, and d) LiNO_3_ electrolytes for various puttering times. C 1s and F 1s XPS spectra for SEI with e) AFA and f) LiNO_3_ electrolytes. g) Reaction mechanism. h) Cryo‐TEM image for SEI and Li‐anodes in AFA electrolytes. Insets are SAED images of SEI and bulk Li. Reproduced with permission.^[^ ^611^ ^]^ Copyright 2022, Wiley VCH.

Xie et al.^[^ ^611^ ^]^ reports design strategies for fluorinated molecules with fluoroalkyl (−CF_2_CF_2_−) as F‐resource. The C−F bond defluorination is improved by leaving species on β‐sites, illustrating the faster kinetics of LiF‐forming reactions (**Figure** **31**a). The 2,2,3,3‐tetrafluorobutane‐1,4‐diol dinitrate (AFA), 1,4‐butanediol dinitrate (BDN), and 2,2,3,3‐tetrafluoro‐1,4‐dimethoxybutane (DFA) activated fluoroalkyls are proposed. The active ending group of −NO_3_ is considered due to the robust parting affinity of −NO_3_ in nucleophilic environments and electrochemical reductions; larger reduction potentials provide key degradation on anodes for SEI generation; 3) corrosion products for AFA degradations, NO_x_^−^ offers compatible additives.^[^ ^612^, ^613^ ^]^ 30.2% mass ratio for F with faster kinetics for release of fluoride awarded by −NO_3_ are united to concentrate robust fluorinated SEI. Fluorinated SEI generation with AFA in Li‐S batteries is examined in Figure 31b–h. F content in SEI with AFA electrolytes is 15% for the surface and 36, 32, 29, and 27 % in‐depth areas for etching time of 40, 80, 120, and 160 s, respectively, whereas with LiNO_3_ or DFA electrolytes is <10% (Figure 31b–d). Such significant F content in SEI for AFA illustrates the role of active ending species in endorsing the release of F with effective building fluorinated SEI. XPS displays fluorocarbon anions obtained from the partial degradation of AFA‐dS after C−S bond cleavage (−CF_2_− peak in F 1s) and minor for LiTFSI; however, it is absent in LiNO_3_ electrolytes. C and F signals of fluorocarbon anions reduce SEI inner layer, illustrating C‐F bond breaking and formation of LiF with multistep kinetics as (detachment of active −S− from AFA‐dS for constructing R−CF_2_CH_2_− residues, and then C−F elimination for generating LiF as SEI inner layer, Figure 31e–g). Uniform and denser SEI (25–30 nm thickness) for AFS on Li‐anodes with crystalline LiF, whereas inhomogeneous 40–50 nm SEI with LiNO_3_, which manifests the uniform Li plate/stripping and superior Li‐ion mobility.^[^ ^614^ ^]^

**Figure 32:**
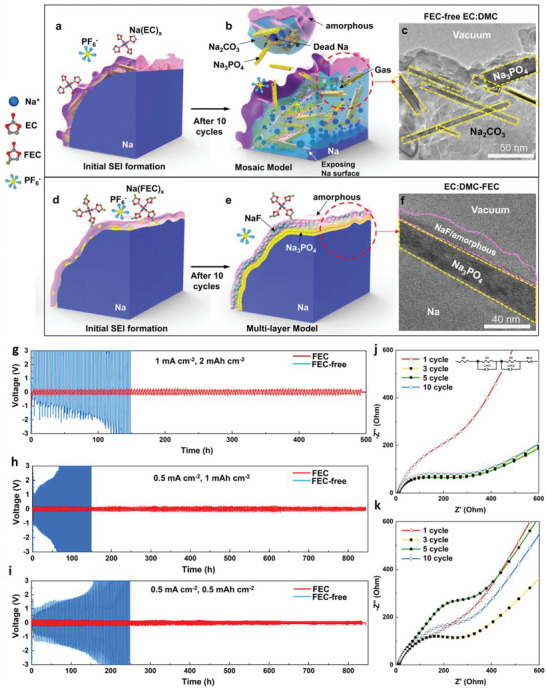
a‐c) SEI nucleation at 1^st^ cycle (a) and final structures after 10 cycle (b) and cryo‐TEM image for FEC‐free EC:DMC electrolytes after 10 cycles (c). d‐f) SEI nucleation at 1^st^ cycle (d) and final structures after 10^th^ cycle (e) and cryo‐TEM image for FEC‐EC:DMC electrolytes after 10^th^ cycles (f). g‐i) Na//Na cell performance and j,k) EIS plots for FEC‐EC:DMC (j) and FEC‐free EC:DMC (k). Reproduced under the terms of a Creative Commons CC BY license.^[^ ^616^ ^]^ Copyright 2021, Nature Publishing Group.

As we know, the electron affinity of EC > DMC; thus, EC is favorably reduced in EC:DMC over the Na‐metal surface.^[^ ^615^ ^]^ Initially, Na^+^‐ions are solvated by EC molecules with reduction of EC (1e^−^) ensuing sodium ethylene decarbonate NaO_2_CO–C_2_H_4_–OCO_2_Na (NEDC) that collects and changes in amorphous organic/polymer layer (**Figure** **32**a–c).^[^ ^616^ ^]^ Poor hydrolytic stability of NEDC further generates Na_2_CO_3_, ethylene, and CO_2_ in the presence of traces of acid or water in electrolytes by SEI destabilization.^[^ ^617^ ^]^ Upon cycling, Na_2_CO_3_ of SEI decomposes to CO_2_ gas, terminating intact SEI. Lucht et al.^[^ ^617^ ^]^ present reactions among the LiPF_6_‐salt and various lithium carbonates that yields Li_x_PF_y_O_z_ and F_2_PO_2_Li. The Na_3_PO_4_ formation also follows similar reactions among the NaPF_6_ and sodium carbonates in the electrolytes. NEDC decomposes larger CO_2_, and the formed Na_3_PO_4_ undergoes disruptions and follows random distributions in the SEI. Na‐dendrites are isolated from hosts owing to inhomogeneous dissolution rates at various regions during stripping, which manifests thicker SEI and Na‐depletion with confined Na metal, needle‐shaped Na_3_PO_4_, larger pieces of Na_2_CO_3_, and mosaic of NEDC polymers (Figure 32b,c). FEC‐EC:DMC forms thinner SEI due to the reaction among the FEC and Na‐metal, forming a uniform film of NaF over the Na_3_PO_4_ surface for the initial stage of SEI.^[^ ^618^ ^]^ The Na_3_PO_4_ and NaF phases impede electrolyte reduction with severe NEDC and CO2 formation decrease. The NEDC and Na_2_CO_3_ are not protected by covering layers and rapidly decompose; thus, the resultant SEI comprises dense inorganic Na_3_PO_4_ and NaF/amorphous composite bilayer structures (Figure 32d–f). Amorphous organic/polymer phases for SEI function significantly due to the protection of SEI by intense volume changes upon cycling, which is intensely associated with battery cycle‐life failure. Na//Na cells with FEC‐EC:DMC displays stable operations for 500–800 h for all current densities; however, FEC‐free EC:DMC deteriorated after 50 cycles for 0.5 mA cm^−2^ smaller current density with catastrophic cell failures for higher currents (Figure 32g–i). Further, FEC‐EC:DMC displays reliable impedance and stable SEI for 1^st^ cycle, whereas FEC‐free EC:DMC induces dynamic changes in SEI, resulting in higher impedance (Figure 32j,k). Ji et al.^[^ ^619^ ^]^ reports FEC additive displays a uniform and thinner SEI for SnSb‐carbon anodes in Na‐ion cells. Besides, Fondard et al.^[^ ^620^ ^]^ proposes the FEC insertion displays reduction of Na_2_CO_3_ formation and decrease in SEI thickness with increasing NaF phase. Many researchers determined complete NaF generation pathways by reduction of FEC for Na‐metals by Monte Carlo/molecular dynamics to predict NaF formation over the SEI top surfaces and limitations of solubility issues for amorphous NEDC.^[^ ^618^, ^621^, ^622^ ^]^

**Figure 33:**
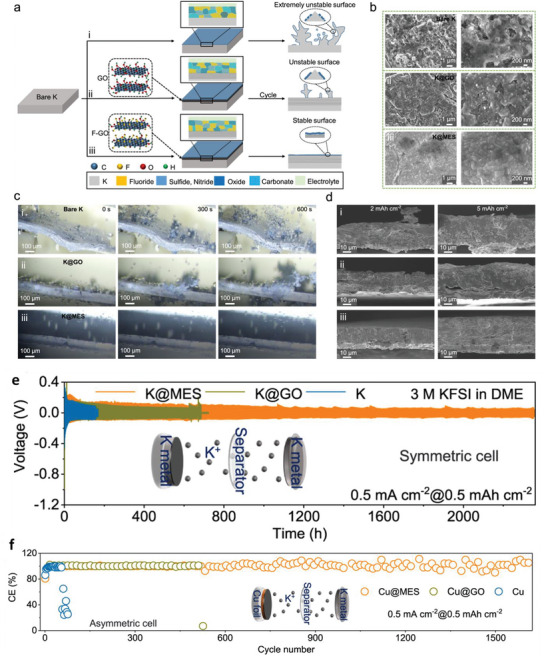
a) Scheme for interface evaluation. b) SEM images for various K‐metal surfaces after cycling. c) In situ optical images for K‐metal depositions. d) Cross‐SEM images of K‐metal deposited for various CCs, (i) bare K, (ii) K@GO, and (iii) K@MES. e) K‐plate/stripping for KFSI electrolyte. f) CEs for Cu//K asymmetric cells. Reproduced under the terms of a Creative Commons CC BY license.^[^ ^623^ ^]^ Copyright 2023, Nature Publishing Group.

Ding et al.^[^ ^623^ ^]^ report the strategies for stabilization of K‐metal interfaces using metal electrode skin (MES) by mimicking human skins. MES consists of GO that can improve in situ SEI generation. GO is fluorinated with partial replacement of oxygen‐functional groups to form F‐GO MES and then extracted over Cu and K‐foils. Wrinkled microstructures with poor flatness and larger undulations for Cu (or K) surfaces are observed, whereas with MES flat and uniform materials coverage in both kinds of electrolytes (3 m KFSI in DME and 0.8 m KPF_6_ in EC/DMC, EC/ DMC  =  1:1), implying superior wettability of electrolytes. Cu@MES//K maintains a stable voltage of ≈4 V with zero current, which implies MES well protects the Cu and the electrolyte did not penetrate F‐GO, whereas bare Cu failed to retain 4 V, which suggests strong corrosion for bare Cu. K@MES displays minimal interface impedance and faster K^+^‐ions kinetics, resulting in stable K‐deposition and SEI without dendrites upon cycling under symmetric cells, whereas K@GO or bare K possesses large unstable impedance, larger overpotentials, and fluctuations. The irregular K plate/stripping shows unstable SEI initially. Upon several cycles, interfaces become more inferior, exacerbating the dendritic growth (i of **Figure** **33**a). GO‐protection for K‐metals temporarily alleviates the stress changes due to volume expansions; however, not able to work for more extended operations, and SEI will not protect interface effectively (ii of Figure 33a). F‐GO interface alternations enhance the surface smoothness of K‐anodes by realizing ion concentrations and uniform electric fields. F‐GO alleviates volume expansion and promotes C‐F bonds, and releases larger F for the construction of F‐rich SEI (interface stability for battery operation life and dendrite‐free plate/strip; iii of Figure 33a). MES for K‐metal deposition evolves smoother surfaces without loose and porous materials compared to GO and bare K. In situ optical images display that a large amount of mossy K and dendrites emerged even for 300–600s for K@GO and bare K, whereas K@MES remains dendrite‐free (Figure 33b,c). Further, Cu@MES shows denser dendrite‐free flat surface loading, while Cu@GO and bare Cu surfaces provide larger volume expansions with severe dendrites for K‐metal deposits (Figure 33d). K@MES displays 2300 h (3 m KFSI in DME) plate/stripping lifetime, while K@GO fails for 670 h. Similarly, Cu@MES//K achieves 1600 cycles; however, Cu@GO//K and Cu//K give 300 and 60 cycles only (Figure 33e,f).

**Figure 34:**
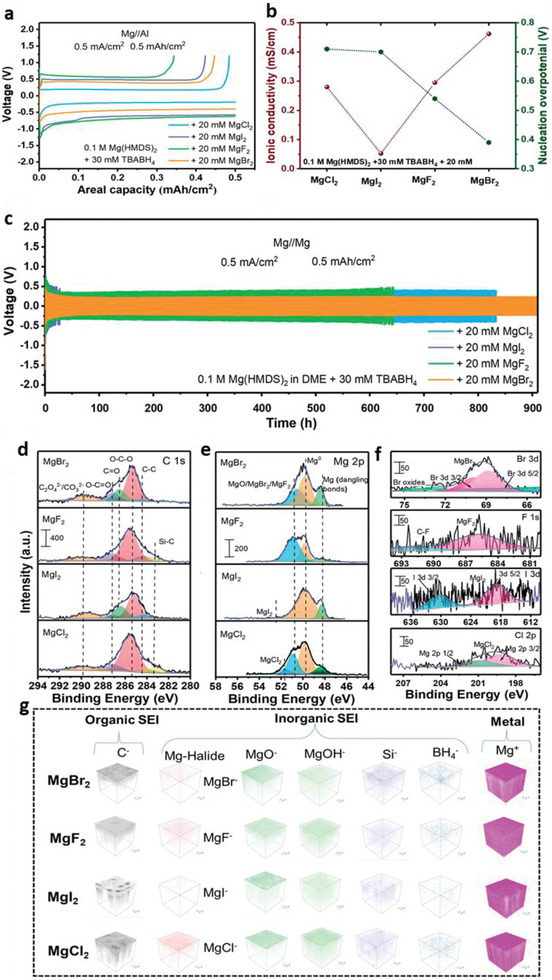
a) Mg plate/strip voltage profiles. b) ion conductivity and nucleation potential. c) Mg//Mg symmetric cells. d‐f) XPS spectra for C 1s, Mg 2p, and halides. g) TOF‐SIMS 3D images after 20 Mg//Mg cycles for Mg anodes. Reproduced with permission.^[^ ^624^ ^]^ Copyright 2023, American Chemical Society.

Chinnadurai et al.^[^ ^624^ ^]^ reports the utilization of a series of Mg halides (MgX_2_, X = Cl, I, F, and Br; **Figure** **34**) as additives for conventional magnesium bis(hexamethyldisilazide) (Mg(HMDS)_2_; 0.1 m) in 1,2‐dimethoxyethane (DME) with a small amount of tetrabutylammonium borohydride (TBABH_4_; 30 mm). The MgBr_2_ has nucleation overpotential (Δn) of 0.39 V lower than those of MgCl_2_ (0.71 V), MgI_2_ (0.70 V), and MgF_2_ (0.54 V), vindicating decreased energy barriers of Mg nucleation with MgBr_2_ additives (*σ_Mg_* ^2+^ ≈0.462 mS cm^−1^). However, MgF2 displays the lowest Ea suffers with poor Mg plating/stripping reversibility. Mg//Mg cells show longer Mg plate/stripping for 460 cycles with MgBr_2_; however, for MgI_2_ (few tens), MgF_2_ (310), and MgCl_2_ (415). XPS for Mg anode‐electrolyte interface exhibits C–C (284.4 eV), O–C–O (285.5 eV), C═O (286.52 eV), O–C═O (287.09 eV), C_2_O_4_–/CO_3_^2−^ (289.68 eV), and Si–C (283.25 eV) in C 1s,^[^ ^625^ ^]^ Mg metal (49.8 eV), MgO/MgBr_2_/MgF_2_ (51.0 eV), MgCl_2_ (51.8 eV), MgI_2_ (52.5 eV), and Mg dangling bonds (48.4 eV) in Mg 2p,^[^ ^624^, ^626^, ^627^ ^]^ MgCl_2_ (199.1 eV), MgI_2_ (618.5 eV), MgF_2_ (685.7 eV), MgBr_2_ (68.75 eV), Si^0^ (99.2–99.5 eV) in halide spectra,^[^ ^624^, ^628^, ^629^, ^630^ ^]^ Si–C (101–104 eV) in Si 2p, and Mg–BH_4_ (188.7 eV) and B‐oxides (192 eV) in B 1s spectra.^[^ ^624^, ^631^ ^]^ TOF‐SIMS images display Mg‐anodes possessing organic‐rich SEI with native Mg oxides (MgO, Mg(OH)_2_) under MgBr_2_ electrolytes. Below organic‐rich SEI, the inorganic SEI with MgBr_2_, Mg(BH_4_)_2_, Mg oxides, and Si components were also observed. MgCl_2_ and MgF_2_ display lower reliability for dispersal organic‐rich SEI, while MgI_2_ shows uneven dispersal of organic species with large agglomeration. MgBr_2_ shows uniform deposition of Mg with crystalline nature due to lower nucleation overpotential. Typically, Mg(HMDS)_2_‐based electrolyte suffers anode passivation due to the moisture contents of electrolytes and interactions with anodes. Halide additives possess a minimal amount of BH_4_^−^‐ions as moisture scavengers and in situ SEI, which implies reversible Mg‐ion transfers. Zhang et al.^[^ ^632^ ^]^ reports Mg(CF_3_SO_3_)_2_‐based electrolyte, in which cycled Mg‐anode surface mainly consists of MgF_2_, MgCl_2_, MgS, MgO. According to the structures, the interphase layers with lower diffusion energy barriers contain MgS‐rich and hybridized polymer frameworks with an effective increase in Mg^2+^‐migrations with obtaining robust interfaces.^[^ ^633^ ^]^

**Figure 35:**
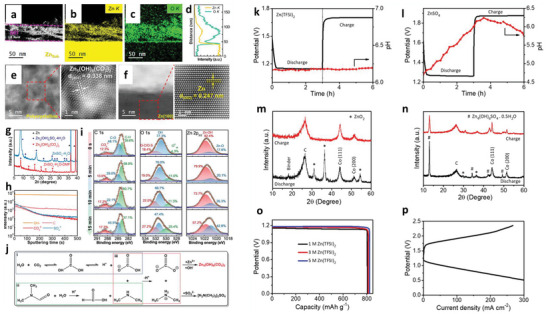
a) TEM image of Zn‐anode under ZnSO_4_‐H_2_O‐DMF. b,c) EDS maps. d) EELS spectra across interfaces. e,f) ABF‐TEM images for polycrystalline SEI and single‐crystalline Zn‐anode, respectively. g) XRD patterns for Zn under ZnSO_4_–H_2_O and ZnSO_4_–H_2_O–DMF. h) TOF‐SIMS, and i) XPS in‐depth spectra in ZnSO_4_–H_2_O–DMF. j) Formation mechanism for Zn_5_(OH)_6_(CO_3_)_2_‐contained SEI. Reproduced with permission.^[^ ^634^ ^]^ Copyright 2023, American Chemical Society. k,l) Charge‐discharge plots for Zn(TFSI)_2_ (k) and ZnSO_4_ (l). m,n) XRD patterns. o) Specific capacity. p) C‐D profiles for Zn(OTf)_2_ electrolyte. Reproduced with permission.^[^ ^204^ ^]^ Copyright 2023, American Chemical Society.

Xiong et al. report controlling strategies for interfacial chemistry using hybrid electrolytes of water and a polar aprotic N,N‐dimethylformamide for altering Zn^2+^‐solvated structures and formation of in situ Zn^2+^‐conducting Zn_5_(CO_3_)_2_(OH)_6_ SEI over the Zn surface.^[^ ^634^ ^]^ In conventional ZnSO_4_ electrolytes, the formation of hydrated Zn‐ions of [Zn(H_2_O)_6_]^2+^ occurs due to reactions of free water molecules with Zn^2+^‐ions. Series of 2 m ZnSO_4_–H_2_O–DMF electrolytes in volume ratios of water to DMF (6:0, 5:1, 4:2, 3:3, 2:4, 1:5, and 0:6) has been reported, in which homogeneous solutions are observed for 5:1 and 4:2 ratios of H_2_O/DMF and recrystallization of ZnSO_4_ occurs for H_2_O/DMF ratio up to 3:3, which suggests DMF alterations for Zn^2+^‐solvation structures that can be destroyed for excess DMF. Theoretical calculations report the pronounced ion‐solvation clusters for Zn^2+^ matched with SO_4_^2−^, H_2_O, and DMF. Radial distribution functions of ZnSO_4_‐H_2_O show primary solvation shell of Zn^2+^ is of 2.5 Å distance, whereas a distinct Zn–O (DMF) at ≈1.88 Å with DMF insertion implies DMF critically integrates Zn^2+^‐solvated structures. Zn^2+^‐BEs has relations as Zn^2+^–SO_4_^2−^ > Zn^2+^–DMF > Zn^2+^–H_2_O, manifests the preferential Zn^2+^ coordinations with DMF instead of H_2_O. Thus lowered coordinating H_2_O and free H_2_O illustrate minimal probability for parasitic reactions during Zn‐plate/strip. Zn//Zn cells display plate/strip process for 1780 and 2500 h under ZnSO_4_–H_2_O–DMF (H_2_O:DMF = 5:1 and 4:2) electrolytes, whereas, ZnSO_4_–H_2_O fails in 92 h with cell‐failure with dendrites. ZnSO_4_–H_2_O has a lower overpotential than ZnSO_4_–H_2_O–DMF due to poor ion conductance and strong solvation effects.^[^ ^635^ ^]^ XRD displays Zn_4_SO_4_(OH)_6_·3H_2_O corrosion byproduct under ZnSO_4_–H_2_O, whereas pure Zn‐phase for ZnSO_4_–H_2_O–DMF. In situ SEI and improved wettability for Zn‐anodes show benefits for R_ct_ and Gibb's free energy reduction for nucleation upon reversible Zn^2+^‐plate/strip.^[^ ^636^ ^]^ Zn surface exhibits numerous protrusions and scattered flakes with a 48.4° contact angle under ZnSO_4_–H_2_O, whereas smooth dendrite‐free surface (contact angle ≈ 0^o^) for ZnSO_4_–H_2_O–DMF electrolyte. **Figure** **35**a displays 60–70 nm thick SEI for Zn‐surface under ZnSO_4_–H_2_O–DMF. EELS (E_p,SEI_ = 19.2 eV)/Zn (E_p,Zn_ = 13.5 eV) heterostructures) and EDS maps reveal weaker Zn signal than pristine Zn, and stronger O reflections suggest less dense SEI and Zn phases (Figure 35b,c). TEM demonstrates Zn_5_(OH)_6_(CO_3_)_2_‐based composite SEI with d‐spacing of 0.338 nm corresponds to (400) crystal‐reflection of Zn_5_(OH)_6_(CO_3_)_2_ phase, whereas Zn‐anode displays crystal plane of (002). Zn‐anodes under ZnSO_4_–H_2_O electrolytes possess Zn_4_SO_4_(OH)_6_·4H_2_O byproduct without SEI (Figure 35e–g). TOF‐SIMS and XPS display OH^−^ (m/e = 17), CO_3_^2−^ (m/e = 60), and C^−^ (m/e = 12, originated from the decomposed CO_3_^2−^) signals for various depths of etching, indicating the formation of Zn_5_(OH)_6_(CO_3_)_2_ for SEI. Dissolved CO_2_ and H_2_O in ZnSO_4_–H_2_O–DMF system obtains equilibrium for step‐i, then DMF hydrolysis occurs for stage‐ii due to acidic environments with the formation of dimethylamine ((CH_3_)_2_NH) and formic acid (HCOOH), later ((CH_3_)_2_NH) responds with HCO_3_^−^ with creation of CO_3_^2−^ species by bicarbonate deprotonation, and then CO_3_^2−^reacts with Zn^2+^ and OH^−^ with forming solidified Zn_5_(OH)_6_(CO_3_)_2_ (Figure 35h–j). Overall reactions are (2HCON(CH_3_)_2_ + 2CO_2_ + 2H_2_O + ZnSO_4_ + 4Zn(OH)_2_ → Zn_5_(OH)_6_(CO_3_)_2_↓ + [H_2_N(CH_3_)_2_]_2_SO_4_ + 2HCOOH). DFT calculations predict a reaction energy of −7.78 eV/Zn_5_(OH)_6_(CO_3_)_2_.^[^ ^637^, ^638^, ^639^ ^]^

Cell operating under Zn(TFSI)_2_ retains stable pH during charge‐discharge, whereas ZnSO_4_ shows an increase in pH (upon discharge) and decrease upon charge, suggesting OH^−^ formation/consumption for discharge/charge, respectively (Figure 35k,l).^[^ ^204^ ^]^ Zn‐anode displays insulating zinc sulfate hydroxide [Zn_4_(OH)_6_SO_4_·0.5H_2_O (ZHS)] formation under ZnSO_4_, confirming poor reversibility (Figure 35m,n) Gradual increase of ZHS upon long operations leads volume expansions with electrolyte depletion, illustrating challenges for cell operations. Faraday's and ideal gas laws determine the number of transferred electrons towards O_2_ through ORR/OER by analyzing O_2_ consumption/evolution and charge transfers. Zn(TFSI)_2_ displayed the reversible changes under pressure relative to original values. Multiple C‐D processes correspond to ZnO_2_ formation with inferior decomposition of H_2_O by 4e^−^ oxygen pathways, whereas ZnSO_4_ has irreversible features. Zn(TFSI)_2_ and Zn(CF_3_SO_3_)_2_ electrochemical properties correspond for bulky TFSI^−^ and CF_3_SO_3_^−^ anions (versus SO_4_^2−^ with double charges, Figure 35o,p), which diminishes the number of water molecules surrounded by Zn^2+^ cations. Typically, Zn(TFSI)_2_ or Zn(OTf)_2_ has an anhydrous form, but ZnSO_4_ is always hydrated. The molecular polarity index of TFSI^−^ (3.8 eV) and OTf^−^ (4.68 eV) is lower relative to SO_4_^2−^ (10.47 eV), implying superior hydrophobicity. The lower electrostatic potential of SO_4_^2−^ than that of TFSI^−^ and OTf^−^ displays the favorable hydrogen bonding by water. Sun et al.^[^ ^640^ ^]^ reports generation of Zn^2+^‐rich and H_2_O‐poor environments with Zn(OTf)_2_ or Zn(TFSI)_2_‐based electrolytes, whereas H_2_O‐rich environments by ZnSO_4_ for inner Helmholtz layers, clarifying faster kinetics for Zn ions. Reactions among the ZnSO_4_ and OH^−^ form the ZHS intermediates limiting the availability of H_2_O or ZnSO_4_ for electrolytes, whereas Zn(TFSI)_2_ did not show consumption of electrolytes, which suggests reversible ZnO_2_ phase formations (Figure 35m,n) well consistent to non‐aqueous Li‐O_2_ cells. Overall reaction kinetics for Zn(TFSI)_2_:^[^ ^640^, ^641^, ^642^ ^]^

(19) $$\begin{array}{c} \mathrm{Anode}:\;\mathrm{Zn}\left(s\right)↔{\mathrm{Zn}}^{2+}\left(\mathrm{aq}\right)+2e^{-}\\ \left[\mathrm{Discharge}:\;\mathrm{Zn}\left(s\right)\to {\mathrm{Zn}}^{2+}\left(\mathrm{aq}\right)+2e^{-}\;\mathrm{and}\;\mathrm{Charge}:\;{\mathrm{Zn}}^{2+}\left(\mathrm{aq}\right)+2e^{-}\to \mathrm{Zn}\left(s\right)\right]\\ \mathrm{Cathode}:O_{2}+2e^{-}↔O_{2}^{2-}\\ \mathrm{Overall}:\;\mathrm{Zn}+O_{2}↔{\mathrm{ZnO}}_{2} \end{array}$$

Overall reaction kinetics for ZnSO_4_:

(20) $$\begin{array}{c} \mathrm{Anode}:\;2\mathrm{Zn}↔2{\mathrm{Zn}}^{2+}+4e^{-}\\ \mathrm{Cathode}:\;O_{2}+2H_{2}O+4e^{-}↔4{\mathrm{OH}}^{-}\\ 4{\mathrm{OH}}^{-}+8/3\;{\mathrm{Zn}}^{2+}+2/3\;{\mathrm{SO}}_{4}^{2-}+1/3\;H_{2}O↔2/3\;{\mathrm{Zn}}_{4}{\left(\mathrm{OH}\right)}_{6}{\mathrm{SO}}_{4}\cdot 0.5H_{2}O↓\\ \mathrm{Overall}:\;2\;\mathrm{Zn}+O_{2}+2/3\;{\mathrm{ZnSO}}_{4}+7/3\;H_{2}O↔2/3\;{\mathrm{Zn}}_{4}{\left(\mathrm{OH}\right)}_{6}{\mathrm{SO}}_{4}\cdot 0.5H_{2}O↓ \end{array}$$

**Figure 36:**
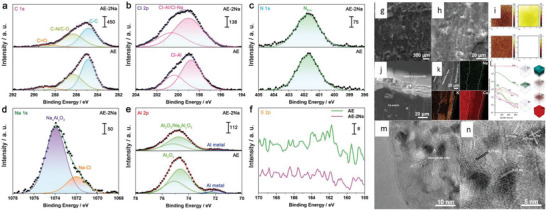
a) XPS spectra for Al electrodes from Al‐S batteries a) C 1s, b) Cl 2p, c) N 1s, d) Na 1s, e) Al 2p, and f) S 2p. Reproduced with permission under the Creative Commons CC BY 4.0 license.^[^ ^643^ ^]^ Copyright 2023, Wiley‐VCH. g‐n) Microstructures and compositions for AHSEL‐Ca anodes. g,h) SEM, i) TOF‐SIMS 2D maps, j,k) SEM and EDS maps. l) TOF‐SIMS 3D maps. m,n) TEM images of SEI. Reproduced with permission.^[^ ^650^ ^]^ Copyright 2023, Wiley‐VCH.

XPS spectra of Al‐metal anode displays C‐C, C‐N/C‐O, and C═O in C 1s, Al‐Cl and/or Na‐Cl in Cl 2p and Na_x_Al_y_O_2_ species in Al‐anode and Al‐anode‐2Na electrolytes (**Figure** **36**a–f).^[^ ^643^ ^]^ Al 2p shows Al‐O bindings of Al_2_O_3_ and/or Na_x_Al_y_O_2_ (74.7 eV for Al 2p_3/2_), whereas metallic Al with small intensity (71.6 eV for Al 2p_3/2_).^[^ ^644^, ^645^ ^]^ Metallic Al has a higher reflection for Al‐anode than Al‐anode‐2Na, implying thicker SEI. Further, smaller intensity for Al_2_O_3_ and/or Na_x_Al_y_O_2_ for Al‐anode‐2Na is due to NaCl additives. S 2p shows stronger polysulfides for Al‐anodes due to larger peaks than Al‐anode‐2Na. Ran et al.^[^ ^562^ ^]^ reports MXene/E‐Al_97_Ce_3_ hybrid anodes stable Al plate/stripping >1000 h with a slightly increased overpotential of 49–54 mV outperforms the pure E‐Al_97_Ce_3_ alloy (280 mV in 210 h) and monometallic Al (1400 mV in 64 h) under aqueous 2 M Al(OTF)_3_ electrolyte. Grafted MXene layer efficiently enables Al^3+^ transport by mitigating native oxide passivation influence for Al plate/strip for E‐Al_97_Ce_3_, boosting localized galvanic intermetallic pairs for Al_11_Ce_3_ and α‐Al nanolamellas corresponding to the monometallic Al.^[^ ^646^ ^]^ Guo et al.^[^ ^647^ ^]^ reports 1‐hexadecyl‐3‐methylimidazolium hexafluorophosphate (HMIH) ILs as anti‐corrosive additives, in which EIS displays adsorption intermediates with Al(OH)_x,ads_ for Al/electrolyte interface under HMIM additives, while H_2_, OH^−^, and Al(OH)_x,ads_) for conventional Al‐surfaces. XPS spectra clarify the α‐AlF_3_, Al‐O, P‐O, C═O, C‐C, C‐O, and C═N bindings with stronger lower BEs shifting after HMIH addition, while pristine Al has α‐Al_2_O_3_, Al(OH_3_), γ‐Al_2_O_3_.^[^ ^648^, ^649^ ^]^

Artificial hybrid solid electrolyte layer (AHSEL, Figure 36g–n) with Na/Ca carbonate and calcium hydride nitride NPs (<10 nm) compressed by C and N moieties is reported for Ca^2+^ with *σ_Ca_* ^2+^ ≈ 0.01 mS cm^−1^ and thickness of 20 µm.^[^ ^650^ ^]^ AHSEL transforms to Na/K/Ca hybrid SEIs consisting of monodispersed NPs of Ca_2_NH and smaller amorphous regions with KPF_6_ electrolyte, implying a reduction of fluoridation for Ca‐depositions. Ca plate/strip over bare Ca under 1 M Ca(PF_6_)_2_ or Ca(BF_4_)_2_ EC/DEC (1:1, v/v) electrolytes displays poor stabilities (<12 h) owing to anion polarizations and corrosions. Further, deposition voltage drops for −5.0 V owing to larger intrinsic resistances by thicker CaF_2_ insulating mass formation.^[^ ^651^ ^]^ Whereas, under KPF_6_ EC/DMC/EMC (1:1:1, v/v/v) electrolyte Ca^2+^‐plate/strip for 800 and 250 h comparable to Ca with NaPF_6_ due to parallel K/Ca‐based SEIs consists of KCa(PO_3_)_3_ (JCPDS:39‐1408), Ca(H_2_PO_4_)_2_ (JCPDS:70‐1381), and CaF_2_ (JCPDS:77‐2093) NPs (<20 nm) over Ca^2+^‐loadings. However, Ca//Ca cell fails after 258 h, implying K/Ca‐based SEIs are still not satisfying due to the lack of organic carbon layers for complete protection of Ca. AHSEL‐Ca rapidly suppresses corrosion and polarizations for 1400 h Ca^2+^‐plate/strip with uniform Ca^2+^‐depositions and Na/K/Ca‐based hybrid SEIs that cover K/Ca mixture followed Ca matrix below. SEI consists of the major crystalline phase of Ca_2_NH (AHSEL–electrolytes interface) and minor phases of hexagonal KCa(PO_3_)_3_, orthorhombic Na_3_(PO_3_)_3_·H_2_O (JSPDS:15‐0740), and cubic KCaF_3_ (JCPDS:03‐0567). TOF‐SIMS and EDS display uniform N, Ca, K, Na distribution in SEI. The 0.25 and 0.29 nm d‐spacing confirms Ca_2_NH (222) and (400) reflections. TOF‐SIMS depth profiles and 3D maps display Ca_2_NH‐rich organic–inorganic Na/K/Ca hybrid SEIs with N‐/C‐rich elements distributions, unlike AHSEL N‐rich Ca_2_NH and K/Ca SEIs with minor organic materials.^[^ ^652^ ^]^ Theoretical calculations verify Ca^2+^‐ions in propylene carbonate have weaker solvation structures with H_2_O hybridization, implying Ca^2+^‐transports under electrolyte and desolvation kinetics for CEI.^[^ ^653^ ^]^ Li et al.^[^ ^654^ ^]^ verifies the enolization redox processes (O═C↔C‐O^−^) of perylene tetra‐carboxylic diimide with 1D Ca^2+^‐diffusion kinetics and lack of covalent‐bond cleavages or reformations are the keys for higher Ca^2+^‐reversibility and rate‐capabilities. Ca‐Se with Ca(B(hfip)_4_)_2_‐DME and Ca_0.25_V_2_O_5_·nH_2_O with Ca(ClO_4_)_2_‐acetonitrile‐H_2_O electrolytes displays reversible CIBs with 180 and 158.2 mAh g^−1^ capacities.^[^ ^655^, ^656^ ^]^

## 9 Conclusions and Future Perspectives

In general, all types of batteries involve positive cathodes, solid/liquid electrolytes, and negative anodes; these components should be synchronized for operating chemistries. Failure of batteries occurs due to the interactions among these constituents. SSBs demonstrate promising outlooks regarding reliability, safety operations, operating temperature windows, and energy densities relative to commercial liquid‐based batteries. However, the ionic conductivity of SEs, volume expansion for electrodes, interfacial resistances, poor cycle life, and flexibility issues are critical challenges for SSBs. Extensive efforts have been considered to overcome these challenges, but the poor interfaces among the electrodes and SEs, EWs, and ion diffusion kinetics impedes the development of SSBs. For well‐functioning SSBs, remarkable electrochemical stability, mechanical integrity, and interfaces with minimal resistance are essential.

Physical or chemical contacts, lattice‐mismatch, space‐charge layers, interdiffusion of elements, chemical reactivity of SEI, and metal dendrites are the key interface issues that severely degrade the cell performances in terms of cell capacity, internal resistances, and cell failures. Multivalent batteries offer substantial attention; however, it critically undergoes challenges for mechanistic understandings of metals (Li, Na, K, Mg, Zn, Al, Ca) nucleation/growth and strategy of interphases and electrodes. There are no standard benchmarks for using electrolytes or electrodes and their design strategies. The overestimated performances based on the gravimetric capacities of particular active or consumed materials did not fully translate under realistic platforms; therefore, careful evaluation for electrode optimization and cell design is essential. Rational design for robust and functional SEI for multivalent metals is also discussed critically.

The employment of high‐energy metals as anode materials realizes that SEs can promise future battery technologies. Ion conductance for monovalent metal‐ions transport is prolonged for application inception; however, multivalent metal‐ion SEs are in the early stages. High conductance and modulus of SIEs compared to SPEs; however, their inferior interfaces towards cathode/anodes limit the required targets of batteries under high current density, rate/areal capacity, and utilizations. Insertion of polymers, gels, or liquids among the electrodes and SIEs can be suitable for developing conformal interface layers to endure great volume changes. The composition of electrolytes describes the metal plate/strip behavior, EWs, and kinetics for M‐intercalations. For SPEs, the focus should be on RT ion conductivity, SEs/anode and SEs/cathodes compatibility and structures, crystallinity, tg, and ion‐pair dissociation. Crosslinking of segments and electron‐removing species for backbones promises electrode‐interface stability. Besides, higher storage modulus and partial amorphous structuring confirm the stable metal deposits for metal anodes. For commercial‐scale SEs, the compatibility of SEs and anode/cathodes is essential because numerous electrons, ions, heat, and pressure are severely reallocated with different features. The fabrication of SEs with abundant resources can be rewarding for the environment and cost.

**Figure 37:**
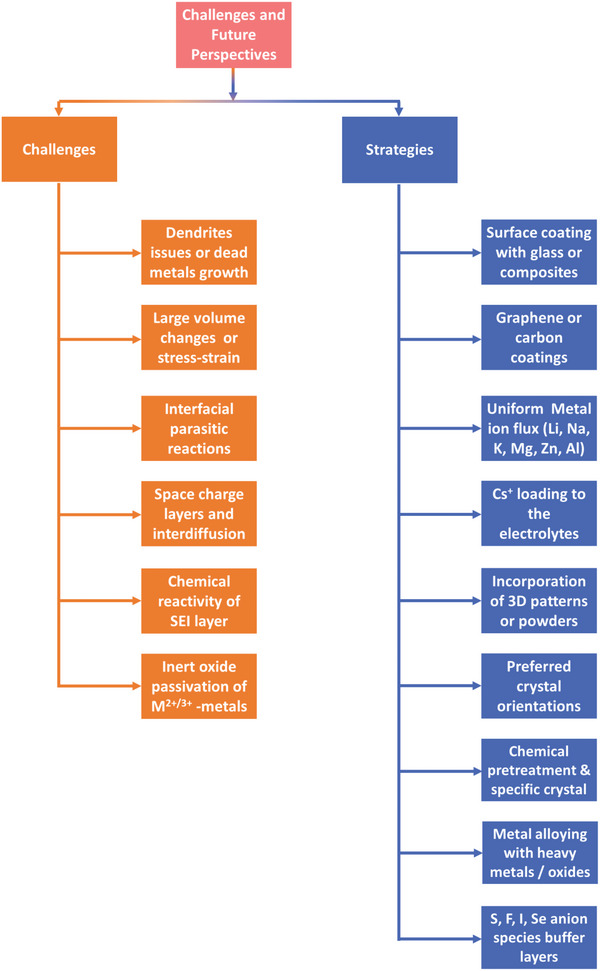
Failure mechanisms and perspectives for the anode interface chemistries.

For the anode, stable metals such as Li, Na, K, Mg, Zn, Al, and Ca can be employed; however, lower utilization, inert oxide passivation, self‐diffusion coefficients, and irreversibility shows a significant loss for energy density. Loading 2D or 3D porous frameworks can enhance mass/charge‐transfer kinetics. Depth of discharge or state of charge outbreaks the metal dendrites, poor plate/strip efficiency, decomposition of SEs with parasitic reactions, surface passivation, or gaseous formation. Measuring protocols, including current densities, temperatures, plate/strip capability, and cell configurations critically modify the metal anode's overall performances and have been considered for evaluation, which are the standard benchmarks for practical mono‐/multivalent‐batteries. A comprehensive understanding of formation kinetics and regulation of flexible SEI layer during cycle operations enable the practical metal batteries. Information on the dynamic structure of SEI needs to investigate on priority, which is the primary approach for SEI modeling. Several elements from SEI will be dissolved for high voltages, and some elements will be reorganized after initial SEI formation deprived of Faraday processes. We proposed several strategies for addressing the present issues of metal‐anodes‐kinetics regardless of battery chemistries (**Figure** **37**): 1) metal surface coating with glasses or composites that exert pressure in contrast to the surfaces and blocks the open spaces, 2) loading of carbon, graphene, or carbon allotropes that restrict depletion of electrolytes and enhances the charge‐discharge capability, 3) uniform metal‐ion flux that can prevent local population of metal‐ions, for instance, better wetting materials coating over separator or additives, 4) Cs^+^‐ions loading for electrolytes that remain positive in charge and repels arriving Li or metal ions from tips causing spherical surface topography, 5) Insertion of 3D patterns, frameworks or metal in powder forms that can improve active surface area by dissipating electron densities for respective current densities, 6) preferred crystal orientation of metals (formation of single crystalline or dominating crystal planes) that decreases nucleation density and improves the diffusion kinetics from 2D to 3D with excess ion‐transport channels, 7) chemical pretreatment with preferred crystal orientations that improves the SEI layer stability and localized current density distribution with lowering diffusion energy barriers, 8) metal‐alloying with heavy metals or oxides, and 9) artificial buffer layer such as S, F, I, or Se anion species that can generate numerous metal vacancies to increase mobile metal ions. For practical approach post‐LIBs, the interface kinetics and electrochemical stability of both electrodes upon charge‐discharge with developing innovative materials is the scope for future research.

## Conflict of Interest

The authors declare no conflict of interest.
